# Isoreticular Expansion and Linker-Enabled Control of Interpenetration in Titanium–Organic Frameworks

**DOI:** 10.1021/jacs.3c06590

**Published:** 2023-09-21

**Authors:** Natalia M. Padial, Clara Chinchilla-Garzón, Neyvis Almora-Barrios, Javier Castells-Gil, Javier González-Platas, Sergio Tatay, Carlos Martí-Gastaldo

**Affiliations:** † Functional Inorganic Materials Team, Instituto de Ciencia Molecular (ICMol), Universitat de València, València 46980, Spain; ‡ School of Chemistry,University of Birmingham, Edgbaston, Birmingham B15 2TT, U.K.; § Departamento de Física, Universitario de Estudios Avanzados en Física Atómica, Molecular y Fotónica (IUDEA). MALTA Consolider Team, Universidad de La Laguna, Avda. Astrofísico Fco. Sánchez s/n, La Laguna, Tenerife E-38204, Spain

## Abstract

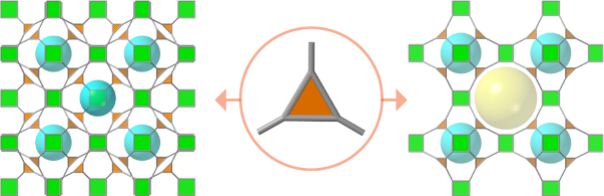

Titanium–organic frameworks offer distinctive opportunities in the realm of metal–organic frameworks (MOFs) due to the integration of intrinsic photoactivity or redox versatility in porous architectures with ultrahigh stability. Unfortunately, the high polarizing power of Ti^4+^ cations makes them prone to hydrolysis, thus preventing the systematic design of these types of frameworks. We illustrate the use of heterobimetallic cluster Ti_2_Ca_2_ as a persistent building unit compatible with the isoreticular design of titanium frameworks. The MUV-12(X) and MUV-12(Y) series can be all synthesized as single crystals by using linkers of varying functionalization and size for the formation of the nets with tailorable porosity and degree of interpenetration. Following the generalization of this approach, we also gain rational control over interpenetration in these nets by designing linkers with varying degrees of steric hindrance to eliminate stacking interactions and access the highest gravimetric surface area reported for titanium(IV) MOFs (3000 m^2^ g^–1^).

## Introduction

Among many other fascinating features, the modular nature of reticular solids is one of the most attractive features for the design of porous materials. In the case of metal–organic frameworks (MOFs), this possibility is enabled by using inorganic secondary building units (SBUs) as nodes with predefined symmetry and connectivity. The structural information encoded in these units can be used to target the assembly of binary frameworks with “default” topologies by reaction with organic linkers, provided that they do not alter SBU formation. This provides a versatile playground for the rational design of isoreticular frameworks with blueprint topologies that are also amenable to controlled expansion and tailorable porosity metrics by using elongated linkers for ultralarge channels (IRMOF-74-XI),^1^ record surface areas (DUT-60, NU-1501),^2,3^ or functionalized linkers for rich pore chemistries.^4^

**Figure 1:**
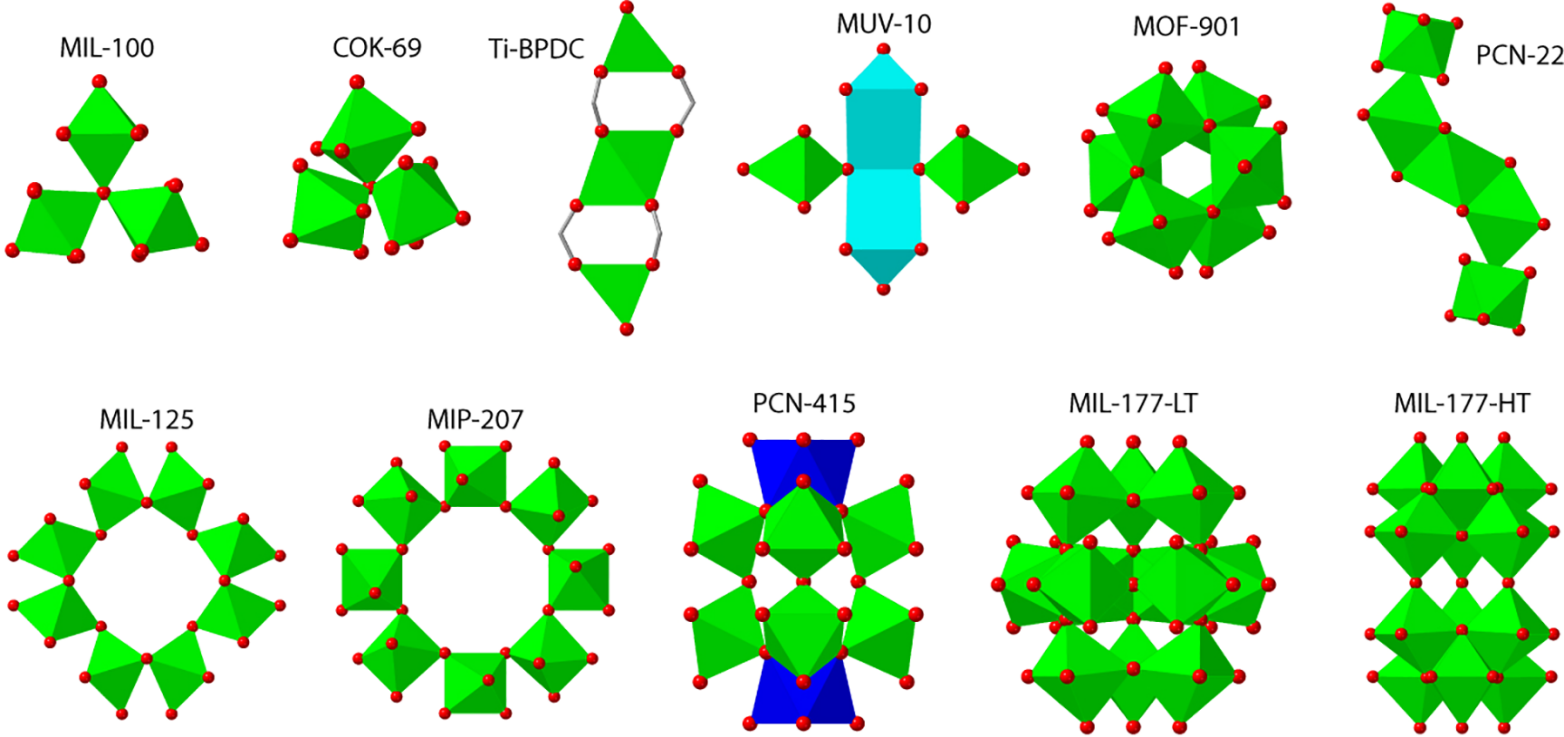
Representative homo- and heterometallic Ti-oxo clusters used in the assembly of titanium(IV) frameworks ordered by increasing nuclearity. Ti (green), Ca (magenta), Zr (navy blue). Despite this diversity, none of them has proven to date to be a persistent SBU compatible with the reticular design of frameworks by direct methods.

Unfortunately, this possibility is still limited to a small number of polynuclear clusters that can be persistently formed under a broad range of synthetic conditions. Archetypical SBU examples such as the 4-connected (4-c) tetrahedral [Zn_4_O(RCO_2_)_6_] in MOF-5,^5^ or planar paddlewheel dimer [Cu_2_(H_2_O)_2_(RCO_2_)_4_] in HKUST-1,^6^ 6-c [Fe_3_O(RCO_2_)_6_] trimers in MIL-100 and MIL-101,^7,8^ 12-c cuboctahedral [Zr_6_O_4_(OH)_4_(RCO_2_)_12_] in UiO-66,^9^ or even clusters with higher connectivity such as 18-c [Re_9_(μ_3_–OH)_8_(μ_2_–OH)_3_(RCO_2_)_18_] in gea-MOF-1^10^ have all demonstrated the value of the SBU as one of the main assets for the rapid development of the field.^11^ The difficulties in controlling the assembly of targeted titanium–organic carboxylate MOFs are good examples of this problem. Though the number of crystalline titanium frameworks prepared by direct synthesis has increased since the report of MIL-125 in 2009,^12^ the difficulties in controlling the chemistry of titanium in solution leads to the unpredictable assembly of targeted Ti-SBUs. Compared to the predictability of Zr_6_ clusters and resulting architectures,^13^ titanium nodes display a richer structural diversity from homo- and heterobimetallic Ti-oxo clusters of multiple nuclearity and symmetry (Figure 1)^14−22^ to rod-type infinite chains.^23−27^ As a result, reticular control is currently only accessible by postsynthetic modification of other MOFs by titanium-exchange reactions^28−30^ or the interlinking of preformed Ti clusters by dynamic covalent bond formation^16^ and linker exchange reactions.^22^

We recently reported the possibility of using the heterometallic Ti cluster [Ti_2_Ca_2_(μ_*3*_-O)_2_(RCO_2_)_8_(H_2_O)_4_] (Ti_2_Ca_2_) to assemble two isoreticular *the* nets: MUV-10^17^ and MUV-12.^31^ Both can be prepared by reaction of the metal salts in acid conditions with either 1,3,5-benzene tricarboxylic acid (H_3_btc) or its expanded version 4′,4″,4”’-benzene-1,3,5-tribenzoic acid (H_3_btb). These results encouraged us to explore whether this SBU might be used as a persistent node amenable to the reticular design of titanium frameworks.

Our results confirm that this cluster is fully compatible with the synthesis of isoreticular MUV-12(X) crystals with tailorable porosities and versatile pore chemistries. We also demonstrate how the interpenetration associated with linker expansion can be overcame by introducing steric restraints to the peripheral 4-carboxyphenyl units of the linker for the assembly of noninterpenetrated structures with surface areas near to 3.000 m^2^ g^–1^, the highest reported for titanium-based MOFs thus far.

## Results and Discussion

### Linker Design and Framework Synthesis

**Figure 2:**
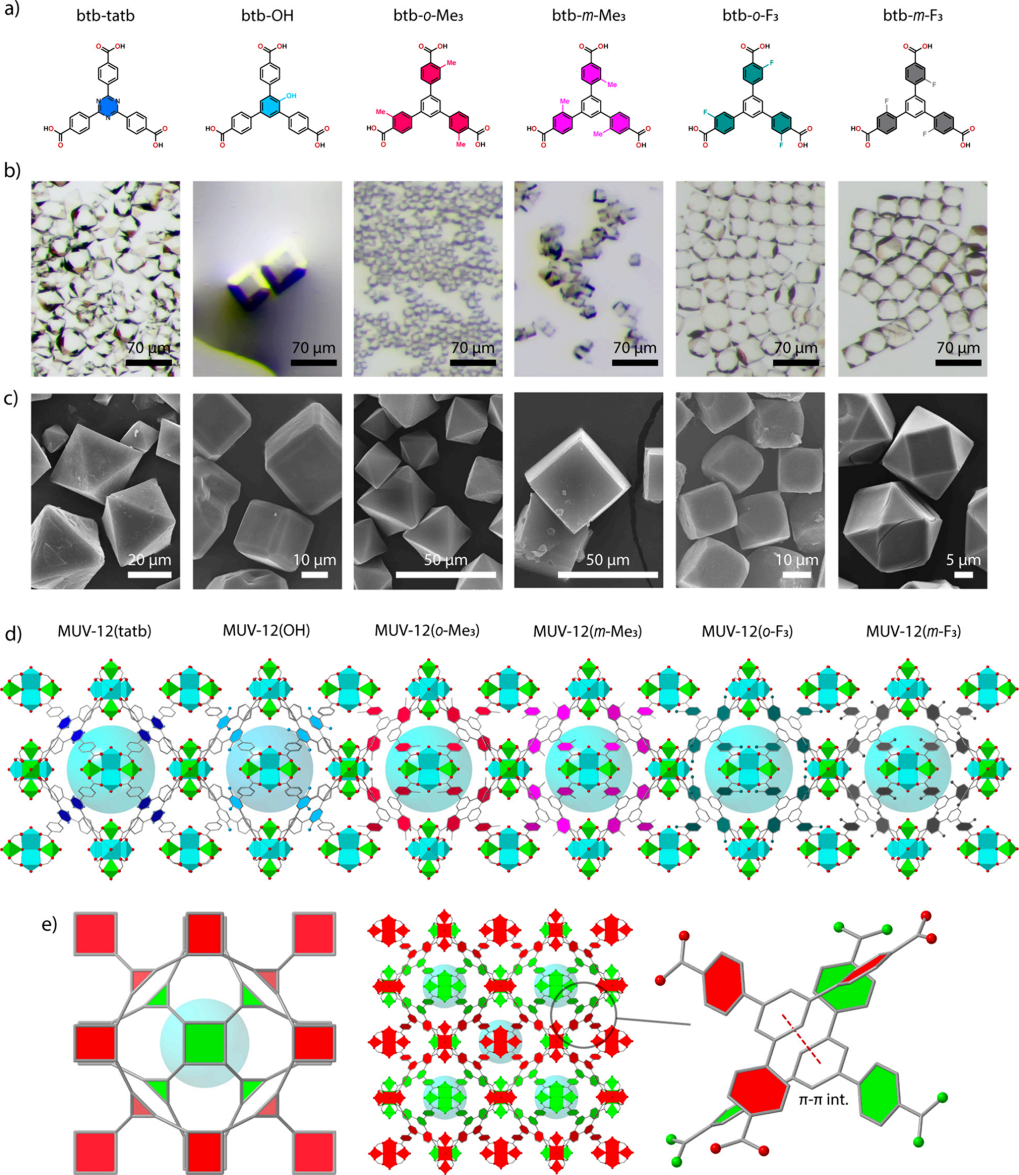
(a) Functionalized tricarboxylic H_3_btb-X linkers used for the assembly of MUV-12(X) MOFs. (b) Optical pictures of the crystals isolated showing their variability in size. (c) SEM pictures showing the changes in size and morphology due to linker functionalization. Focal plane has been adjusted for a clearer view of the crystal morphology. (d) Structure of the MUV-12(X) family showing the changes in pore functionalization and the sodalite-type octahedral cavities accessible (blue sphere). (e) Two-color scheme for clearer visualization of the doubly interpenetrated structure of the frameworks and their corresponding *the-c* topology. The highlight shows the π–π interactions between neighboring nets that favor catenation.

Although our recent results with MUV-12 preliminarily confirmed the possibility of using expanded linkers to direct MOF assembly,^31^ whether the Ti_2_Ca_2_ cluster might be robust enough to enable linker functionalization for systematic framework design remained an open question. Here, we opted to use or design linkers with different substituents in the central and peripheral aromatic rings (Figure 2a). 4,4′,4″-*s*-Triazine-2,4,6-triyl-tribenzoic acid (H_3_tatb) and 5′-(4-carboxyphenyl)-2′-hydroxy-[1,1′:3′,1″-terphenyl]-4,4″-dicarboxylic acid (H_3_btb–OH) are commercially available. As for peripheral aromatic substitution, we synthesized two sets of methylated and fluorinated linkers in 4-carboxyphenyl *ortho* and *meta* positions: 4,4″-dimethyl-5′-(4-carboxy-2-fluorophenyl)-3,3″-difluoro[1,1′:3′,1″-terphenyl]-4,4″-dicarboxylic acid (H_3_btb(*o*-F_3_)), 4,4″-dimethyl-3′,3″-difluoro-5′-[3-fluoro-4(methoxycarbonyl)phenyl)-[1,1′:3′,1″-terphenyl]-4,4″-dicarboxylic acid (H_3_btb(*m*-F_3_)), 5′-(4-carboxy-3-methylphenyl)-3,3″-dimethyl-[1,1′:3′,1″-terphenyl]-4,4″-dicarboxylic acid (H_3_btb(*o*-Me_3_)), and 5′-(4-carboxy-2-methylphenyl)-2,2″-dimethyl-[1,1′:3′,1″-terphenyl]-4,4″-dicarboxylic acid (H_3_btb(*m*-Me_3_)). They were synthesized by Suzuki–Miyaura cross-coupling reactions of the corresponding methyl esters with 1,3,5-tris(3,3,4,4-tetramethylborolan-1-yl)benzene followed by saponification of the ester. All synthetic details and associated characterization are summarized in the Supplementary Section S2. This functionalization scheme was expected to generalize the synthetic value of our approach by introducing minimum steric restraint and modifying the acidity of the linker with substituents of varying electron-donating and electron-withdrawing nature.

MUV-12(X) (X = tatb, OH, *o*-F_3_, *m*-F_3_, *o*-Me_3_, and *m*-Me_3_) were synthesized in PTFE bottles by reaction of titanium(IV) isopropoxide (65 μmol) with a mixture of calcium chloride (65 μmol), acetic acid (0.5–1 mL), and the corresponding linker in *N,N*-dimethylformamide (DMF) at 120 °C for 48 h. These sets of general conditions led to the formation of micrometric crystals in all cases except for the *o*-F_3_ derivative (Figure 2b). We used an automated high-throughput (HT) platform to accelerate the discovery of appropriate synthetic variables in this case. MUV-12(*o*-F_3_) crystals could be finally isolated by using comparatively milder temperatures (115 °C) and longer reaction times (72 h). This suggests that the problematics often associated with the synthesis of MOFs with functionalized linkers are not necessarily conceptual but synthetic in nature^32^ and can be overcome by systematic survey of the chemical space.

### Compositional and Structural Analysis

Analysis of all the crystals with scanning electron microscopy (SEM) shows varying sizes between 10 and 50 μm with several morphologies that include octahedras, slightly chamfered cubes, cubes, truncated octahedras, and cuboctahedras (Figure 2c). Inductively coupled plasma mass spectroscopy (ICP-MS) was used to confirm minimum deviations from the theoretical Ti:Ca ratios expected for the formation of Ti_2_Ca_2_ clusters (Supplementary Section S6.4). Single-crystal X-ray diffraction data (SCXR) collected with Synchrotron radiation (ALBA, BL13-XALOC) displayed very weak diffraction at high angles in all cases. Diffraction data was used to identify the space group and cell parameters required to guide the corresponding Rietveld refinements by using the reported structure of MUV-12 (CCDC 2018540) as a starting model.^31^ All refinements converged with excellent statistics and residual values for cubic *Im*–3 space groups with the cell axis oscillating between 26.13 and 26.53 Å (Supplementary Section S4.2). As shown in Figure 2d, all solids are isostructural and display a doubly interpenetrated structure built from the assembly of 8-c TiCa clusters with the corresponding tricarboxylic linkers that act as 3-c nodes for (3,8)-connected *the-c* topologies.^33^ Compared to the isoreticular MUV-10 analogue, linker expansion results in the generation of larger octahedral cavities despite catenation, with crystallographic diameters between 1.7 and 1.9 nm depending on the linker functionality and the corresponding pore occupation. Translational entanglement is favored by π–π interactions of variable strength (from 3.20 to 3.61 Å) between the central aromatic rings of linkers belonging to neighboring nets (Figure 2e).

### Effect of Substitution in Framework Properties and Porosity

**Figure 3:**
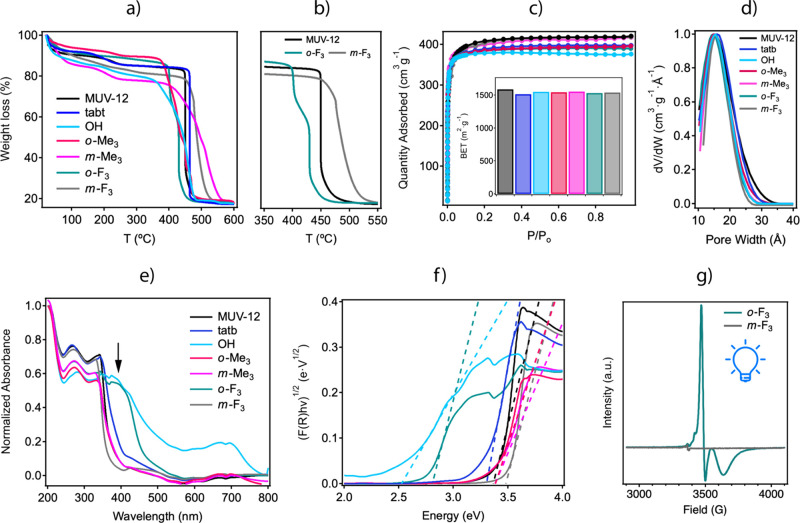
(a) TGA plots in air for the MUV-12(X) family. (b) Zoom showing the differences in thermal stability for the *o*-F_3_ and *m*-F_3_ derivatives. (c) N_2_ adsorption isotherms and the corresponding multipoint BET surface areas. (d) PSD plots calculated with nonlinear DFT methods confirming the presence of a narrow micropore distribution in all cases. (e) Diffuse reflectance spectra showing the onset of visible light absorption for the OH and *o*-F_3_ derivatives. (f) Linear fits to the Tauc plot approximation showing the corresponding optical band gaps. (g) EPR spectra after irradiation with a blue led light (440 nm) of the *o*-F_3_ and *m*-F_3_ frameworks confirming the photoinduced generation of Ti^3+^ radicals only for the *ortho* isomer.

Besides the impact of linker functionalization on challenging the formation of isoreticular frameworks due to changes in the acidity of the carboxylate groups, we were also interested in understanding the effect of this substitution on the thermal and chemical stabilities of the resulting MOFs. Figure 3a shows the thermogravimetric decomposition profiles of all solids in air. Compared to MUV-12 that decomposes at 450 °C, substitution of the central ring shifts the decomposition temperature to a broader temperature interval between 430 and 490 °C depending on the functional group. The most interesting feature is observed for the *o*-F_3_ and *m*-F_3_ derivatives that decompose at the minimum and maximum temperatures, suggesting a different effect of fluorination on the strength of the coordination linkage depending on the substituent position (Figure 3b). To test the chemical stability of these frameworks, we analyzed with ICP-MS the supernatants after incubation under static conditions of MUV-12(X) in water for 24 h at pH = 7. Our data indicates negligible leaching indicative of high chemical stability in water (Supplementary Section S7). The concentration of titanium leached remains below 0.015% in all cases, although it is true that central ring substitution is negative from this point of view since MUV-12 displays the minimum leaching of the series (0.005%).

Based on the chemical stability of the MUV-12(X) family, we did not take special precautions for the evacuation of the crystals. All solids were exchanged with acetone and evacuated for 12 h at 60 °C and 10^–6^ Torr. All N_2_ adsorption isotherms show nonhysteretic type-I curves characteristic of microporous materials (Figure 3c). Compared to the gas uptake displayed by pristine MUV-12, we observed minor differences for the functionalized frameworks. The corresponding experimental BET surface area values oscillate between 1500 and 1600 m^2^ g^–1^ in all. According to the geometrical surface areas calculated from the structural models available by using Molovol,^34^ the effects of steric crowding imposed by the linker functionalization to the pore volume are quite small in all cases (Supplementary Section S6.1 and S6.2). This agrees well with the minimum variations revealed by the experimental pore size distribution (PSD), which shows sharp distributions centered at micropores with diameters between 1.6 and 1.4 nm (Figure 3d).

### Effect of Constitutional Isomerism over Light Absorption

The combination of high surface areas with the possibility of tailoring the light absorption and effective band gap in these molecular frameworks is one of the main reasons behind the interest attracted by titanium MOFs in photocatalysis.^35^ Compared with TiO_2_, these solids can be optically engineered by adequate modification of the inorganic cluster or the organic linker to promote effective charge transfer for the photogeneration of catalytically active Ti^3+^ sites. Regarding linker functionalization, the engineering of the optical response of MIL-125 with mono- and disubstituted amino terephtalic acids is possibly the most paradigmatic case.^36^ All MUV-12(X) frameworks are colorless with the exception of the btb–OH and *o*-F_3_ derivatives that are yellow. We collected the UV–vis of all solids using an integrating sphere to analyze in more detail the effect of functionalization on their optical properties. As anticipated by their colors, the absorption is centered in the UV region in all cases, except for MUV-12(OH) and MUV-12(*o*-F_3_), which show an additional absorption band centered at 400 nm (Figure 3e). These correspond to optical band gaps of 2.5 and 2.8 eV, estimated from the diffuse reflectance spectra by the linear fit of the Tauc plot (Figure 3f). These values are very close to the 2.6 eV reported for MIL-125-NH_2_,^36^ and they are narrower than the band gaps between 3.3 and 3.5 eV displayed by bare MUV-12 and the other functionalized MUV-12(X) frameworks.

Compared to the substitution of terephthalic acid that is symmetrically equivalent, the use of 5′-phenyl-terphenyl grants access to constitutional (also called structural) isomers by substitution in *ortho* or *meta* positions of the peripheral aromatic ring. The distinctive behavior of MUV-12(*o*-F_3_) was quite surprising as both linkers, H_3_btb-*m*-F_3_ and H_3_btb-*o*-F_3_, are colorless in solution, and their UV–vis spectrum does not show any absorption band in the visible region, suggesting that the onset of visible absorption for the last is induced by its reticulation. Based on this, we argued that charge separation with visible light involving photoinduced ligand-to-metal charge (LMCT) transfer would be only effective for the *ortho* isomer. This was confirmed by irradiating a suspension of MUV-12(*m*-F_3_) and MUV-12(*o*-F_3_) in acetonitrile with a blue light (440 nm). Only the *ortho* isomer displayed a change in color from yellow to dark brown, which can be associated with the photogeneration of Ti^3+^ species consistent with the appearance of a paramagnetic signal at 0.35 T in the EPR spectra only for this isomer (Figure 3g).

### Linker-Enabled Control of Interpenetration in MUV-12 Frameworks

When long organic linkers are used to expand a certain net, particularly those based on a cubic symmetry as MUV-12, this often results in the formation of two independent frameworks that are mechanically entangled to occupy what otherwise would be larger pores.^37^ Though detrimental to porosity, interpenetration can be useful to improve the mechanical properties and stability of the framework, controlling its structural response^38^ or improving gas selectivity.^39,40^ But how can interpenetration be controlled? The factors governing interpenetration are not easy to identify and still lack a systematic understanding. There are precedents that confirm the importance of high temperatures and concentrations in favoring the formation of thermodynamic interpenetrated networks compared to kinetically favored noninterpenetrated forms obtained at lower temperatures and concentrations.^41,42^ Combined with the use of bulky solvents, this same principle can be translated into frameworks with variable levels of interpenetration.^43^ The use of bulky substituents^44,45^ or hindered linkers^46,47^ can be also effective in favoring the formation of noninterpenetrated crystals by crowding the pores available to prevent catenation or constraining linker conformations required to form the interpenetrated form.

**Figure 4:**
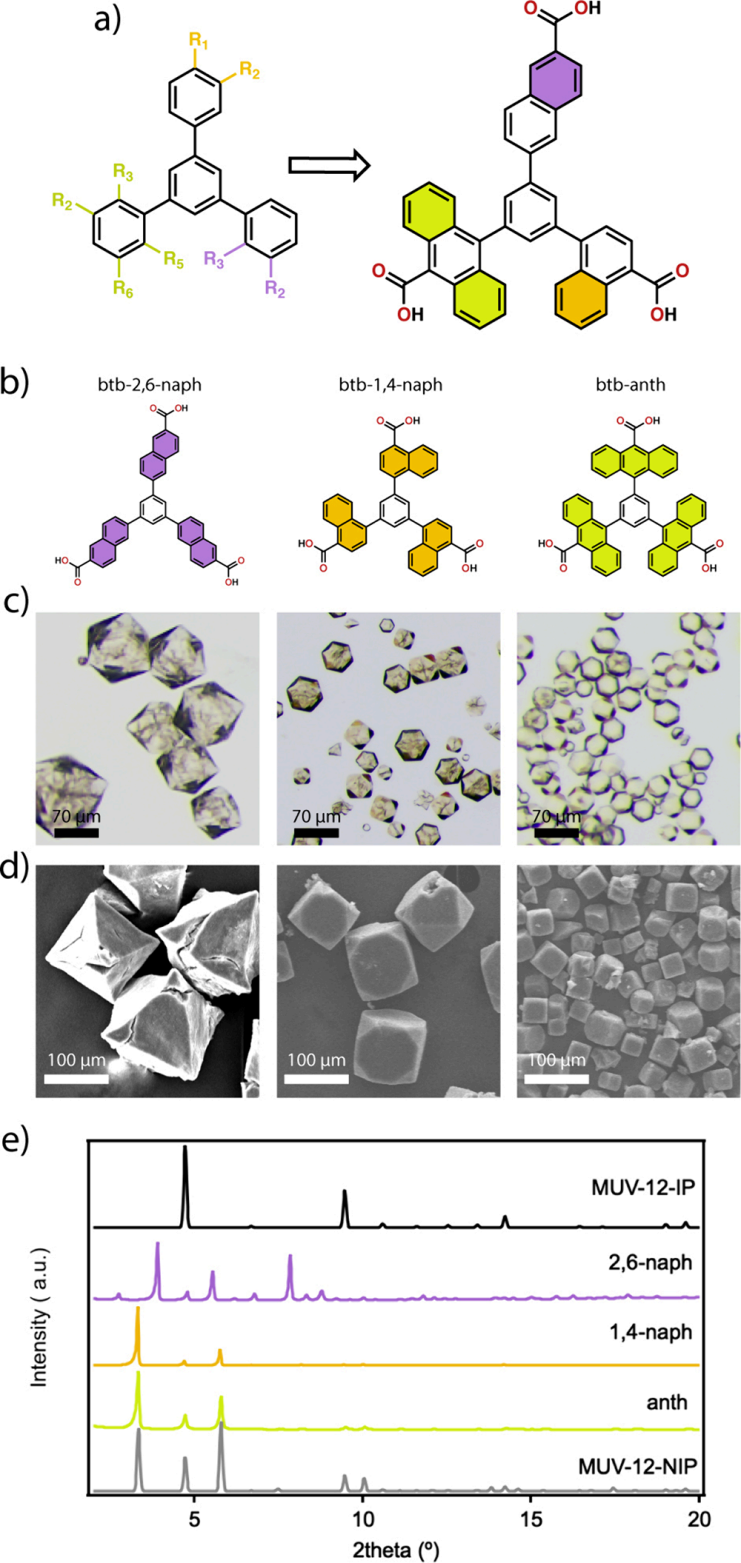
(a) Modification of the btb linker backbone to access higher steric congestion at variable positions in peripheral benzoate units. (b) btb-Y linkers with variable steric congestion used for the assembly of MUV-12(Y) MOFs. (c) Optical and (d) SEM pictures of the crystals isolated. (e) PXRD patterns of MUV-12(Y) frameworks and the simulated patterns for the interpenetrated (IP) and noninterpenetrated (NIP) structures of MUV-12.

The chemistry of titanium in solution imposes severe limitations on the formation of crystalline phases under kinetic control at low temperatures or concentrations. Also, our experiments with bulkier methoxy or trifluoromethyl substituents (-OMe and -OBu_3_) did not enable formation of crystalline solids with the conditions used for the other MUV-12(X) systems. This pushed us to look for alternative linkers that imposed a high level of steric congestion without the influence of other inductive effects that might affect the assembly of the framework. As shown in Figure 4a, we chose to incorporate additional aromatic rings in different positions to 4-carboxyphenyl for variable levels of constraint.

Figure 4b shows the family of btb-Y linkers used: 6,6′,6″-(1,3,5-benzenetriyl)tris[2-naphthalenecarboxylic acid] (2,6-naph), 4,4′,4″-(1,3,5-benzenetriyl)tris[1-napthalencarboxylic acid] (1,4-naph), and dimethyl 10,10′-(5-(3-(methoxycarbonyl)anthracene-1-yl)-1,3-phenylene)bis(anthracene-9-carboxylic acid) (anth) were prepared according to the general procedures followed for the rest of linkers (Supplementary Section S2). MUV-12(Y) (Y = 1,4-naph and anth) was synthesized from the same metal precursors and modulator concentrations used for the MUV-12(X) series described before. In turn, MUV-12(2,6-naph) crystals could only be isolated at the same concentrations by replacing DMF with fresh *N,N’*-diethylformamide (DEF). DMF or aged DEF solvents led to the formation of polycrystalline materials, suggesting a direct influence of the solvent in the assembly of this framework. Optical microscopy and SEM confirm the formation of yellowish micrometric crystals with an impact of the linker in their size and morphology (Figure 4c,d). EDX analysis was used to confirm the 1:1 Ca:Ti ratio, indicative of the presence of Ti_2_Ca_2_ heterobimetallic clusters. Bulk purity was first evaluated with TGA analysis, which agrees with the formation of solid residues consistent with the decomposition of [Ti_3_Ca_3_(μ_*3*_-O)_3_(Y)_4_(H_2_O)_6_] frameworks; Y= 1,4-naph and anth (Supplementary Section S6.5).

**Figure 5:**
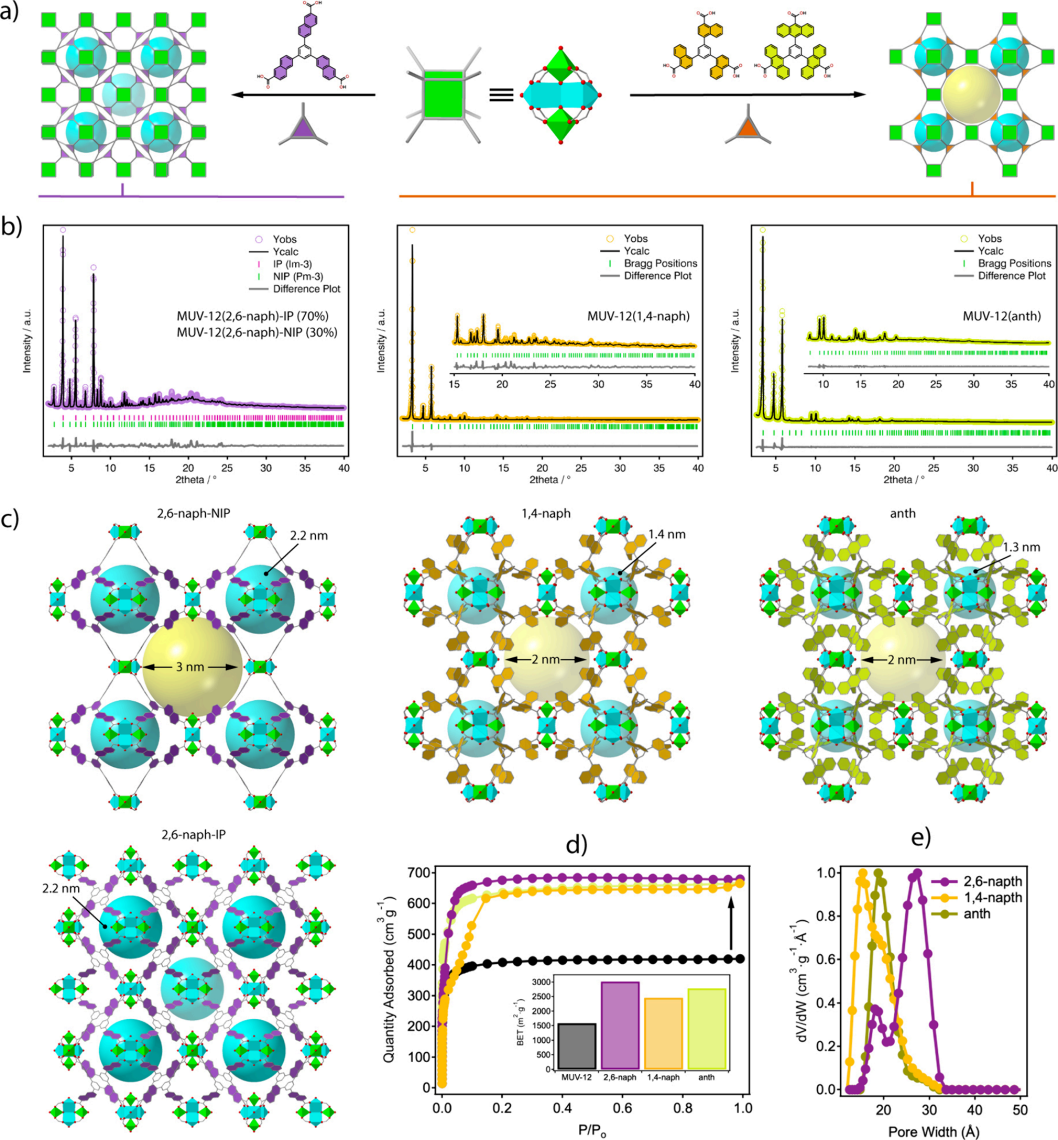
(a) Use of heterometallic Ti_2_Ca_2_ SBU for the assembly of interpenetrated (left) and not-interpenetrated (right) *the* frameworks controlled by the steric restrain imposed by the linkers. (b) Experimental (linker color), calculated (black line), difference plot [(*I*_obs_ – *I*_calc_)] (gray line, bottom panel), and Bragg positions (green ticks) for the Rietveld refinements of the powder diffraction data of MUV-12(Y) frameworks after exchange with acetone. See Supplementary Section S4 for more details. (c) Structure of the MUV-12(Y) family showing how interpenetration constrains the porosity to octahedral cavities (blue spheres), whereas these are combined with comparatively bigger cuboctahedral cavities (yellow spheres) in the noninterpenetrated frameworks. The size of the cavities is controlled by the linker length and its degree of substitution. (d) N_2_ adsorption isotherms and multipoint BET surface areas and the (e) corresponding PSD plots calculated with nonlinear DFT methods.

Figure 4e shows a comparison of the powder diffraction of the as-made solids with the simulated patterns for the interpenetrated (IP) and noninterpenetrated (NIP) structures of MUV-12. The last one was generated computationally for this analysis. Comparison of the diffraction of MUV-12(1,4-naph) and MUV-12(anth) suggests the formation of NIP frameworks under the default MUV-10 *the* topology with a shift to lower angular values of the [100], [110], and [111] diffraction lines, indicative of the expansion of the cubic cell associated with the expansion of the linker length. The pattern of MUV-12(2,6-naph) on the other hand does not fully correspond to the diffraction expected for an NIP phase as it shows additional diffraction lines that could be indicative of interpenetration or the formation of an additional phase. In any case, the larger size of the ligand in this case makes the comparative analysis even more difficult. As summarized in Figure 5a, this family of linkers can be subdivided in two types depending on their internal length and steric constrain for the assembly of IP (*the-c*) or NIP (*the*) cubic frameworks from the assembly of 3-c linkers with 8-c Ti_2_Ca_2_ nodes.

For a clearer understanding of the structure of these frameworks, we attempted single-crystal X-ray diffraction in all cases. Unfortunately, the intertwined nature of the crystals formed only enabled us to determine the structure of MUV-12(anth) with SCXR diffraction data, although at a limited resolution (CCDC 2270773). We collected high-resolution diffraction and Rietveld refined the corresponding patterns by using the corresponding structural models generated with Materials Studio MS (2017) R2 for all MUV-12(Y) frameworks (Figure 5b,c). As anticipated by its diffraction pattern, MUV-12(2,6-naph) could not be converged in any of the single IP or NIP models generated for this framework. The experimental data correspond instead to a combination of both phases with relative weights near to IP (*Im*–3):NIP (*Pm*–3) 69.0(8):31.0(8)% with cell parameters of *a* = 31.771(3) and 31.760(3) Å, respectively. Although the model converged for the IP phase reveals weaker π–π interactions (3.85 Å), compared with those encountered in MUV-12(X) frameworks, this change does not seem sufficient to prevent catenation entirely. Although the relative weights of both phases extracted from the Rietveld refinement are similar to the relative occupancy factors reported for the partially interpenetrated (PIP) sublattices of NOTT-202^39^ and MUF-9,^43^ the absence of a single crystal structural model does not allow to confirm if partial interpenetration takes place at the crystal level or it corresponds instead to a combination of IP and NIP phases in the bulk. MUV-12(1,4-naph) and MUV-12-(anth) in turn correspond to cubic noninterpenetrated (*Pm*–3) frameworks with unit cell parameters of 26.448(1) and 26.253(1) Å, thus confirming the effect of peripheral benzoate substitution in preventing interpenetration.

As evidenced by their topological representation (Figure 5a), interpenetration will result in only one type of octahedral cavity available in the underlying net just like those available for the isoreticular MUV-12(X) series, compared to the combination of octahedral and cuboctahedral cavities that are instead present in the NIP *the* frameworks. However, these considerations are exclusively based on the underlying connectivity of these nets and do not include the potential effect of pore occupation associated with the linker substitution. The characteristics of this family of linkers and the corresponding IP and NIP frameworks were ideal to directly compare the effects in the porosity of these solids of either linker expansion at expense of interpenetration (2,6-naph) or avoiding interpenetration by steric constraint for partial pore occupation (1,4-naph and anth). The analysis of the porous structures of the frameworks reveals interesting features (Figure 5c). In the case of MUV-12(2,6-naph), the pore structure of the IP and NIP networks must be analyzed separately. The former clearly shows the effect of ligand expansion on the size of a single available pore. Compared to the MUV-12(X) series cavities that reach near 1.6 nm, the incorporation of an additional aromatic ring doubles this pore to 2.2 nm. This same cavity remains intact in the NIP phase, but the removal of one of the sublattices generates a cuboctahedral mesopore of near 3 nm. This cavity is comparatively smaller in the 1,4-naph and anth networks, where it is reduced to 2 nm because of the reduction in the size of the linker. These frameworks also reveal a direct effect of single (1,4-naph) and double (anth) ligand substitution on the size of the octahedral cavities, which are reduced by about 40% compared with the 2,6-naph case. These relative changes agree well with the experimental N_2_ gas adsorption isotherms (Figure 5d). Compared to MUV-12, we observe an increase in the saturated N_2_ uptakes, total BET surface areas, and pore volumes that result from tailoring the porosity metrics. These values oscillate between 3020 to 2460 m^2^ g^–1^ and 0.94 and 0.90 cm^3^ g^–1^ throughout the series. MUV-12(2,6-naph) and MUV-12(1,4-naph) also show type-IV isotherm characteristic of the coexistence of micro- and mesopores. Both show inflection points at very low relative pressures indicative of the filling of mesopores, which can be more clearly seen for the second. Despite interpenetration, MUV-12(2,6-naph) displays the highest surface area reported for any titanium(IV)-based MOF by almost doubling the 1550 m^2^ g^–1^ of MIL-125^12^ or PCN-415,^18^ confirming the value of isoreticular expansion to size up porosity also in these frameworks. It is worth noting that the stabilization of Ti^III^ metal centers for the assembly of MIL-100 and MIL-101 analogues has proven useful for the assembly of titanium(III) frameworks with record gravimetric surface areas,^48,49^ in particular when using electrochemical methods as exemplified by the near to 6.000 m^2^ g^–1^ reported for Ti^III^-MIL-100-tatb.^49^ PSD plots were calculated by nonlinear DFT methods with the same kernel used for analyzing the MUV-12(X) series (Figure 5e). MUV-12(2,6-naph) shows two distinct pores at 2.8 and 1.9 nm, confirming the coexistence of the aforementioned NIP and IP phases in this material. The PSD plot of the 1,4-naph MOF is also consistent with the structure of the material but shows a wider distribution encompassing a microporous contribution centered at 1.5 nm and a shoulder near 2.0 nm that agrees with the size of its cuboctahedral cavity. In contrast, the PSD of MUV-12(anth) is dominated exclusively by this type of cavity, suggesting that the steric congestion resulting from double substitution is sufficient to prevent the octahedral cavities from contributing to the gas uptake.

### Computational Analysis of Interpenetration Preference

For a clearer understanding of the tendency to catenation of the MUV-12(X) and MUV-12(Y) families, we performed DFT computational calculations for the guest free structural models corresponding to the interpenetrated and noninterpenetrated phases. For the sake of comparison, all structures were generated with Materials Studio and minimized in energy with the program VASP.^50,51^ The simulated unit cell parameters showed good agreement with the experimental values in the cases for which these were available (Table S11).

**Figure 6:**
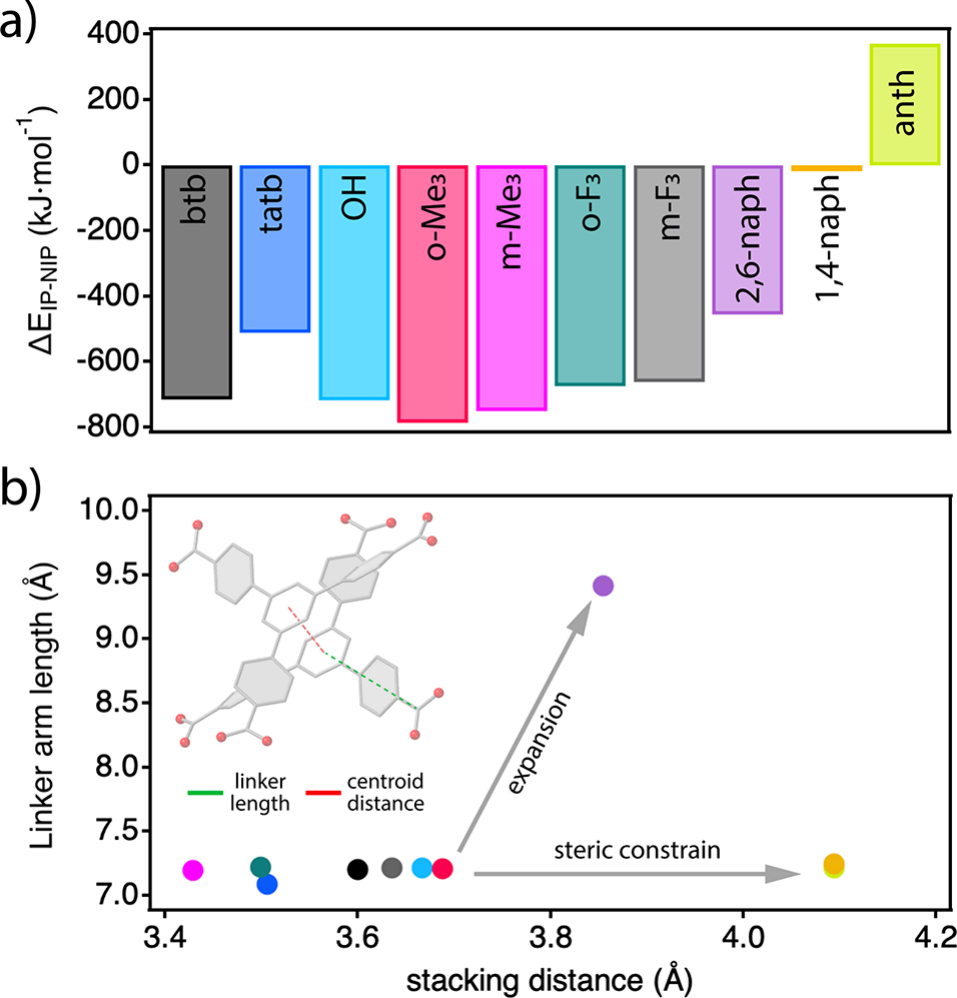
(a) Thermodynamic preference toward interpenetration for MUV-12 frameworks with btb-X and Y linkers. (b) Effect of linker substitution in modifying π–π stacking interactions in the interpenetrated structure of the corresponding MOF.

Our calculations confirm a strong thermodynamic preference toward the formation of IP phases in all MUV-12(X) frameworks with very large Δ*E*_IP-NIP_ energy differences oscillating between −713 and −511 kJ mol^–1^ (Figure 6a). This “interpenetration enthalpy” is much larger than the values near 250 kJ mol^–1^ reported for MUF-10^43^ or NU-1200^52^ by using this same methodology. This is likely due to the absence of π–π interactions in their catenated structures compared to their prevalence in our case. As for the MUV-12(Y) subfamily, we observe a decrease in the tendency for catenation of the 2,6-naph MOF, which becomes negligible or even disfavored (370 kJ mol^–1^) for the NIP 1,4-naph and anth analogues, in good agreement with our experimental results.

To correlate these energy differences with the geometrical and structural parameters related to the linker modification, we represented the length of the linker arm, defined as the distance between the centroid of the central aromatic ring and the carboxylate group, versus the stacking distance, defined as the centroid-to-centroid distances separating the catenated nets (Figure 6b). All of these distances were extracted from the simulated frameworks generated specifically for the analysis, which were validated with the experimental values for all of the catenated frameworks available (Table S11). The data suggest that the effect of the substituents in IP MUV-12(X) frameworks is not sufficient to prevent π–π interactions for only small fluctuations in the stacking distances within 0.25 Å. The effects of linker modification are much more acute for the MUV-12(Y) series. The steric constrain imposed by the presence in 1,4-naph and anth of additional aromatic rings in the 4-carboxymethyl unit, despite being compliant with MUV-12(X) linker lengths, is much more effective in neglecting π–π stacking interactions in the computationally generated IP structures, resulting in the experimental observation of NIP MUV-12(1,4-naph) and MUV-12(anth) frameworks. The substitution of the 4-carboxyphenyl groups by longer 6-carboxynaphtyl units in 2,6-naph expands the linker arm near 2 Å for a longer stacking distance (less effective π–π interactions), that is probably the reason for which this MOF cannot be experimentally isolated as a pure IP phase.

## Conclusions

Gaining control over the design and assembly of titanium frameworks is crucial to generalize their use and access their full potential without the restrictions imposed by the handful of examples currently available. In this sense, the possibility of using principles central to the design of reticular solids, such as their isoreticular expansion for the systematic control of pore size, is arguably one of the main milestones.

We demonstrate this possibility by using Ti_2_Ca_2_ nodes not only to assemble the first isoreticular family of titanium MOFs but also to gain control over their degree of interpenetration. Among the MUV-12(X) and Y series, MUV-12(2,6-naph) combines meso- and microporosity for a record surface area of near to 3000 m^2^ g^–1^, doubling that of other reference titanium frameworks widely used.

The versatility of this cluster as a secondary building unit is probably not limited to the systematic design of the nets, and we are currently exploring its use in the assembly of other topologies compatible with its symmetry and connectivity index. Combined with the ability of these clusters to accommodate other metal ions for alternative heterobimetallic units and tailorable function,^17,53,54^ enlarging the topological diversity of this family of materials might offer appealing opportunities not only in capture, storage, or separation technologies but in catalysis and photoredox chemistry.

## Supporting Information Available

Synthetic and experimental details; physical characterization and supporting tables and figures (synthesis schemes; crystallographic information; experimental, calculated, difference plot, and Bragg positions for the Rietveld refinement; optical images; N_2_ adsorption/desorption isotherm, pore size distribution, multipoint BET analysis and main parameters calculated from the multipoint BET analysis; comparisons of N_2_ isotherm and pore size distribution results; summary of the experimental adsorption data; summary of computational analysis; SEM images; mapping and EDX analysis; experimental metal content determined by ICP-MS; TGA analysis results; UV–vis spectra; Tauc plot and summary of experimental optical band-gap values calculated by the Tauc method; metal concentrations in solution; percentage of Ti leached into the supernatants determined by ICP-MS; EPR measurements; cell parameters from Rietveld refinements and calculated DFT, Δ*E*_IP-NIP_ energy differences, and the centroid-to-centroid distances; ^1^H NMR, ^13^C NMR, DEPT-135 NMR, and ^19^F NMR spectra) (PDF)
