# Supplementary material for: Isoreticular Expansion and Linker-Enabled Control of Interpenetration in Titanium–Organic Frameworks
Source: J Am Chem Soc. 2023 Sep 21;145(39):21397–407. doi: 10.1021/jacs.3c06590 (PMC10853965; doi:10.1021/jacs.3c06590)
Supplement: Supplementary file 1 — ja3c06590_si_001.pdf [file ja3c06590_si_001.pdf]

Supporting Information

Isoreticular Expansion and Linker Enabled Control of Interpenetration in Titanium-Organic Frameworks

Natalia M. Padial,*,‡ Clara Chinchilla-Garzón,‡ Neyvis Almora-Barrios, Javier Castells-Gil, Javier González-Platas, Sergio Tatay and Carlos Martí-Gastaldo.*

‡ These authors contributed equally

*natalia.munoz@uv.es

*carlos.marti@uv.es

TABLE OF CONTENTS

S.1. GENERAL CONSIDERATIONS: STARTING MATERIALS AND CHARACTERIZATION *3*

S1.1. Materials and reagents 3

S.1.2. Physical and chemical characterization 4

S.2. SYNTHESES OF ORGANIC LINKERS *6*

S.2.1. Synthesis of 1,3,5-tris(3,3,4,4-tetramethylborolan-1-yl)benzene (1) 6

S.2.2. General Procedure A: synthesis of the esters 6

S.2.3. General Procedure B: Saponification 9

S.3. SYNTHESIS OF THE MATERIALS *12*

S.3.1. Synthesis of MUV-12 12

S.3.2. Synthesis of MUV-12(tatb) 12

S.3.3. Synthesis of MUV-12(OH) 12

S.3.4. Synthesis of MUV-12(*o*-F)$_3$ 12

S.3.4. Synthesis of MUV-12(*m*-F)$_3$ 13

S.3.5. Synthesis of MUV-12(*o*-Me)$_3$ 13

S.3.6. Synthesis of MUV-12(*m*-Me)$_3$ 13

S.3.7. Synthesis of MUV-12(1,4-napth) 13

S.3.8. Synthesis of MUV-12(2,6-napth) 13

S.3.9. Synthesis of MUV-12(anth) 13

S.4. X- RAY DIFFRACTION (XRD) *14*

S.4.1. Single Crystal X-Ray Diffraction 14

S.4.2. Le Bail & Rietveld Refinements 16

S.5. OPTICAL IMAGES *25*

S.6. CHARACTERIZATION OF THE MATERIALS *27*

S.6.1. Analysis of N_2 Adsorption/Desorption Isotherms at 77 K 27

S.6.2. Pore Volume and Surface Area calculations 41

S.6.3. Scanning Electron Microscopy (SEM-EDX) 42

S.6.4. Inductively Coupled Plasma Mass Spectrometry (ICP-MS) 53

S.6.5. Thermogravimetric Analysis (TG-SDTA) 54

S.6.6. UV-Vis Diffuse Reflectance Spectroscopy 60

S.7. CHEMICAL STABILITY *62*

S.8. ELECTRON PARAMAGNETIC RESONANCE (EPR) *63*

S.9. COMPUTATIONAL METHODS *64*

S.10. NMR SPECTRA *65*

S.11. REFERENCES *98*

S.1. GENERAL CONSIDERATIONS: STARTING MATERIALS AND CHARACTERIZATION

S1.1. Materials and reagents

All purchased reagents and solvents were used without any previous purification. Commercially available chemicals were obtained from BLD pharm, Fluorochem, Merk, TCI, Alfa Aesar and Scharlab. Methyl-4-bromonaphtalene-1-carboxylate (97.0%) Methyl-6-bromo-2-naphthoate (99.89%) Methyl 4-bromo-2-fluorobenzoate (99.51%) Methyl 4-bromo-3-methylbenzoate (98.0%), Ethyl 10-bromoanthracene-9-carboxylate (97.0%), Methyl 4-bromo-3-fluorobenzoate (98.0%), (4-(tert-Butoxycarbonyl)phenyl)boronic acid (98.0%), 4-Methoxycarbonylphenylboronic acid (98.0%), [1,1':3',1''-Terphenyl]-4,4''-dicarboxylic acid, 5'-(4-carboxyphenyl)-2'-hydroxy-[1,1':3'.1-terphenyl]-4,4''-dicarboxylic acid (H_3btb-OH, 98.0%) and methyl-4-bromo-3-methylbenzoate (98%) were purchased from BLDpharm. 1,1'-Bis(diphenylphosphino)ferrocenepalladium (II) dichloride (98.0%) were purchased from Fluorochem. Potassium carbonate (K_2CO_3), 3,5-Tribromobenzene (98%), triethylamine (Et_3N, ≥ 95.0%), sodium hydroxide (NaOH, ≥ 98.0%), sodium sulfate (Na_2SO_4, anhydrous granular, ≥ 99.0%), Calcium (II) chloride anhydrous granular ($CaCl_2$, ≥ 93.0%), titanium (IV) isopropoxide ($Ti(O^iPr)_4$, 97.0%), 1,4-dioxane (≥ 93.0%), 4,4',4''-*s*-Triazine-2,4,6-triyl-tribenzoic acid (H_3tatb, 95.0%), Methyl 4-iodobenzoate (97.0%), Acetonitrile (MeCN, anhydrous, 99.8%) and benzoic acid (BA, C_6H_5COOH, ≥ 99.5%) were purchased from Merk. Tetrakis(triphenylphosphine)palladium (0) [$Pd(PPh_3)_4$, > 97.0%], bis(pinacolato)diboron (99.0%), di-tert-butyl dicarbonate ((Boc_2) O, > 95.0%), cesium fluoride (CsF) and *N,N*-Diethylformamide (DEF, >99.0%) were purchased from TCI Europe. Trifluoroacetic acid (TFA, CF_3COOH, 99.5+%) was purchased from Alfa Aesar. *N,N*-Dimethylformamide (DMF, ≥ 99.8%), acetone, *n*-hexane (> 99.0%), ethyl acetate (EtOAc, > 99.0%), dichloromethane (DCM, ≥ 99.9%) and acetic acid (CH_3COOH, 99.7%) were purchased from Scharlab. Chloroform-*d* ($CDCl_3$, 99.80%) and dimethylsulfoxide-d_6 (DMSO-d_6, 99.80%) were purchased from Eurisotop.

Analytical thin layer chromatography (TLC) was performed on plates using TLC Silica gel 60 F_{254} from Merk. Visualization was accomplished with short-waved (254 nm) UV light. Flash chromatography was performed on Acros Organics ultrapure silica gel (40-60 μm, 60A) using standard techniques eluding with solvents as indicated. Ultrapure water from Milli-Q equipment was used when required.

The synthesis of $[Ti_6O_6(O^iPr)_6(4\text{-tbbz})_6]$ (4-tbbz = 4-tert-butylbenzoic acid; Ti_6) was carried out according to a previously reported procedure.[1]

S.1.2. Physical and chemical characterization

- **FLEX SHAKE** high-throughput workstation from Chemspeed© for robotic dispensing of solids and liquids.

- **Scanning Electron Microscopy (SEM)** and single Energy-Dispersive X-Ray analysis (EDX): particle morphologies, dimensions and mapping were studied with a Hitachi S-4800 scanning electron microscope at an acceleration voltage of 20 kV, over metalized samples with a mixture of gold and palladium for 90 seconds.

- **X-Ray Diffraction (XDR)** patterns were collected in a PANanlytical X'Pert PRO diffractometer using cooper radiation (Cu Kα = 1.5418 $\dot{A}$) with an X'Celerator detector, operating at 40 mA and 45 kV. Profiles were collected in the 3º $< 2\theta <$ 40º range with a step size of 0.017º.

- **Single crystal X-Ray Diffraction.** Data was collected with synchrotron radiation in ALBA Synchrotron (BL13-XALOC) beamline. The diffraction pattern was indexed using the images taken from Dectris Pilatus 6M detector with the program CrysAlisPro 1.171.42.63a (Rigaku OD, 2022) obtaining a maximum resolution of 1.00 Å (Q = 21.38°). Data reduction, scaling and absorption corrections also was performed using CrysAlisPro 1.171.42.63a.

- **Powder X-Ray Diffraction (XDR) patterns for refinement** were collected for polycrystalline samples using a 0.5 mm glass capillary mounted and aligned in a PANanlytical Empyrean diffractometer using copper radiation (Cu Kα = 1.5418 $\dot{A}$) with an PIXcel detector, operating at 40 mA and 45 kV. Profiles were collected by using a Soller Slit of 0.02° and a divergence slit of $1/_4$ at room temperature in the angular range 3º $< 2\theta <$ 105º range with a step size of 0.013º. LeBail profile fitting and Rietveld structural refinements were carried out with TOPAS Academic v6 (http://www.topas-academic.net/).[2]

- **Optical images** were acquired with a ZEISS SteREO Discovery.V8. Crisp and Brilliant Images throughout the Whole 8:1 Manual Zoom Range.

- **Thermogravimetric analysis (TGA)** was carried out with a TGA 550 (Waters/TA Instruments) apparatus between 25 and 650ºC under ambient conditions (20ºC min^{-1} scan rate and an air flow of 30 $mL \cdot min^{-1}$).

- **Gas Adsorption** measurements were recorded on a Micromeritics 3Flex apparatus. The sample was degassed overnight at 60 °C and 10^{-6} Torr prior to analysis. Surface area, pore size and volume values were calculated from N_2 adsorption-desorption isotherms (77 K). Specific surface area was calculated by multi-point Brunauer-Emmett-Teller (BET) method. Total pore volume values were taken at P/P_0=0.96. Pore size distributions were analysed by using the solid density functional theory SWNT for the adsorption branch by assuming a cylindrical pore model.

- **^{1}H, ^{13}C, DEPT-135 and ^{19}F Nuclear Magnetic Resonance (NMR)** spectra were recorded on a Bruker AVIII 300 MHz, AV 400 MHz and NEO 500 MHz instruments and are calibrated using residual undeuterated solvent ($CHCl_3$, DMSO at 7.26 and 2.50 ppm for ^{1}H NMR, respectively, and 77.16 and 39.52 ppm for ^{13}C NMR). The following abbreviations were used to explain multiplicities: s = singlet, d = doublet, t = triplet, q = quartet, m = multiplet, br = broad.

- **High Resolution Mass Spectrometry (HRMS).** High resolution quadrupole time-of-flight mass spectrometer, QTOF (LC-MS/MS). The experiments were carried out with TripleTOF™ 5600 LC/MS/MS System, (AB SCIEX) apparatus. Ionization source: ESI/APCI. Temperature: 450 ºC, ion spray voltage (ISVF): 5500.

- **Inductively Coupled Plasma Mass Spectrometry (ICP-MS)** measurements were carried out with an Agilent 7900 apparatus.

- **UV-Vis Diffuse Reflectance Spectroscopy (DRS)** measurements were performed on a Jasco V-670 spectrophotometer using an integrated Labsphere in the range 200-800 nm.

S.2. SYNTHESES OF ORGANIC LINKERS

S.2.1. Synthesis of 1,3,5-tris(3,3,4,4-tetramethylborolan-1-yl)benzene (1)

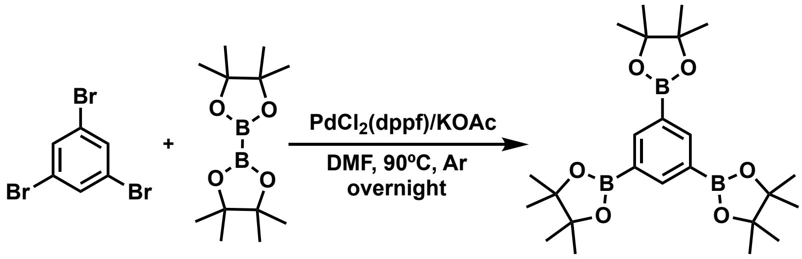

1 (98%)

Scheme S1. Synthesis of the 1,3,5-tris(3,3,4,4-tetramethylborolan-1-yl) benzene **(1)**.

To a 100 mL double-necked, round-bottomed flask, 1,3,5-tribromobenzene (2.0 g, 6.4 mmol), bis(pinacolato)diboron (5.1 g, 20.1 mmol), 1,1'-bis(diphenylphosphino)ferrocene-palladium(II) dichloride dichloromethane (0.16 g, 0.2 mmol), and KOAc (3.8 g, 38.2 mmol) were added. The flask was connected to Schlenk line and evacuated air and refilled with the nitrogen. 20 mL of *N,N*-dimethylformamide (DMF) was degassed (one hour) and added through a canula. The resulting mixture was stirred vigorously and heated at 90ºC under N_2 overnight. After cooling down to room temperature, deionized water (200 mL) was added to the cooled mixture resulting in a dark black precipitate. Upon filtration, the product was obtained as a black solid (98% yield) which was used for the next step without any additional purification. **^{1}H NMR (300 MHz, $CDCl_3$)** δ: 8.35 (s, 3H), 1.33 (s, 36H) ppm.

S.2.2. General Procedure A: synthesis of the esters

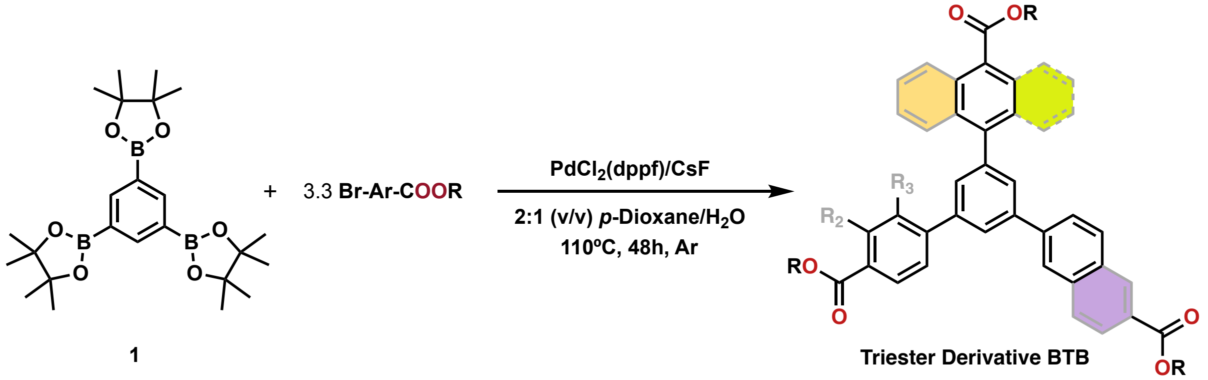

Scheme S2. Syntheses of Triester Derivatives H_3btb-X and H_3btb-Y based on Suzuki-Miyaura cross-coupling reaction.

Following the previously reported procedure[3] to a 250 mL double-necked, round-bottomed flask, compound **1** (3.00 g, 6.58 mmol), the corresponding bromo derivate (21.61 mmol), CsF (7.20 g, 47.40 mmol) and Pd(dppf)Cl_2 (0.3 g, 0.41 mmol) were added. The flask was connected to Schlenk line and deoxygenated and protected under Ar atmosphere. After adding 150 mL mixed solvent of ρ-dioxane/H_2O (2:1 *v/v*), which was previously degassed for one hour, the flask was equipped with a reflux under inert atmosphere at 110ºC for 48 hours with vigorously stirring. After that, the mixture was cooled down to room temperature, diluted with CH_2Cl_2, washed with 5% HCl, brine and dried over anhydrous Na_2SO_4. After removing CH_2Cl_2 solvent by rotary evaporation, the crude product was chromatographed over silica gel to afford the corresponding alkyl ester.

Synthesis of 4,4''-Dimethyl 5'-[4-(methoxycarbonyl)phenyl][1,1':3',1''-terphenyl]-4,4''-dicarboxylate (2)

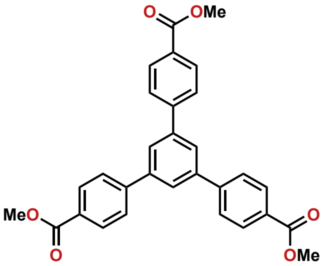

Following the general *procedure A*, reaction of the compound **1** (3.0 g, 6.58 mmol) with methyl 4-iodobenzoate (5.17 g, 19.73 mmol). Purification by column chromatography [silica gel, Hexane/EtOAc (8:2)] afforded 4.00 g, 8.32 mmol (72%) of the title compound **2** as a white solid. NMR data match with the previously reported in the literature.[4] ^{1}H NMR (300 MHz, $CDCl_3$) δ: 8.14 (d, J = 8.7 Hz, 6H), 7.83 (s, 3H), 7.74 (d, J = 8.8 Hz, 6H), 3.95 (s, 9H) ppm. R_f (hexane: ethyl acetate, 8:2): 0.55.

Synthesis of 4,4''-Dimethyl 2,2''-difluoro-5'-[2-fluoro-4-(methoxycarbonyl)phenyl)-[1,1':3',1''-terphenyl]-4,4''-dicarboxylate (3)

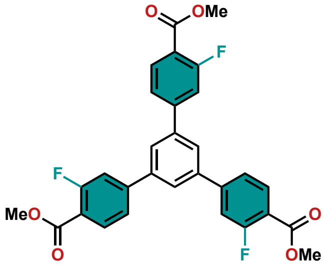

Following the general *procedure A*, reaction of the compound **1** (2.73 g, 5.98 mmol) with methyl 4-bromo-2-flurobenzoate (4.80 g, 19.50 mmol). Purification by column chromatography [silica gel, Hexane/EtOAc (7:3)] afforded 1.85 g, 3.46 mmol (58%) of the title compound **3** as a dark brown solid. NMR data match with the previously reported in the literature.[3] ^{1}H NMR (300 MHz, $CDCl_3$) δ: 8.08 (t, J = 7.9 Hz, 3H), 7.83 (s, 3H), 7.53 (dd, J = 8.0, 1.7 Hz, 3H), 7.47 (dd, J = 11.6, 1.5 Hz, 3H), 3.98 (s, 9H) ppm. ^{19}F NMR (282 MHz, $CDCl_3$) δ: -117.25 ppm. R_f (hexane: ethyl acetate, 7:3): 0.28.

Synthesis of 4,4''-Dimethyl 3',3''-difluoro-5'-[3-fluoro-4-(methoxycarbonyl)phenyl)-[1,1':3',1''-terphenyl]-4,4''-dicarboxylate (4)

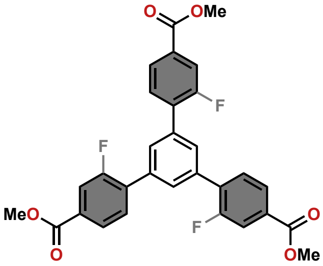

Following the general *procedure A*, reaction of the compound **1** (2.73 g, 5.98 mmol) with methyl 4-bromo-3-flurobenzoate (4.80 g, 19.50 mmol). Purification by column chromatography [silica gel, Hexane/EtOAc (7:3)] afforded 2.05 g, 3.83 mmol (64 %) of the title compound **4** as dark brown solid. ^{1}H NMR (300 MHz, $CDCl_3$) δ: 7.90 (dd, J = 8.1, 1.7 Hz, 3H), 7.83 (dd, J = 11.0, 1.6 Hz, 3H), 7.78 (brs, 3H), 7.58 (t, J = 7.8 Hz, 3H), 3.94 (s, 9H) ppm. ^{13}C NMR (126 MHz, DEPT-135, $CDCl_3$) δ: 166.2 (C), 159.8 (d, J = 249.5 Hz, C), 136.0 (C), 133.1 (d, J = 13.5 Hz, C), 131.9 (d, J = 7.8 Hz, C), 131.2 (d, J = 3.2 Hz, CH), 130.0 (CH), 126.1 (d, J = 3.6 Hz, CH), 117.9 (d, J = 24.8 Hz, CH), 52.9 (CH_3) ppm. ^{19}F NMR (282 MHz, $CDCl_3$) δ: -117.27 ppm. HRMS (ESI, m/z) for $C_{27}H_{15}F_3O_6$: Calc. $[M + H]^+$: 535.1363; found: 535.1368. R_f (hexane: ethyl acetate, 7:3): 0.36.

Synthesis of 4,4''-Dimethyl-5'-(4-(methoxycarbonyl)-3-methylphenyl)-3,3''-dimethyl-[1,1':3',1''-terpenyl]-4,4''-dicarboxylate (5)

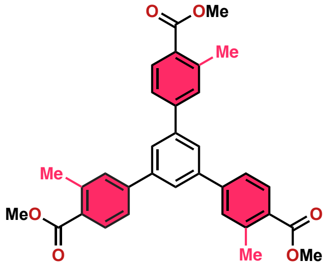

Following the general *procedure A*, reaction of the compound **1** (3.00 g, 6.58 mmol) with methyl-4-bromo-2-methylbenzoate (4.95 g, 21.61 mmol). Purification by column chromatography [silica gel, Hexane/EtOAc (8:2)] afforded 2.75 g, 5.15 mmol (70%) of the title compound **5** as a pale brown solid. NMR data match with the previously reported in the literature.[3] ^{1}H NMR (300 MHz, $CDCl_3$) δ: 8.05 (d, J = 8.8 Hz, 3H), 7.82 (s, 3H), 7.57 (ddt, J = 3.8, 1.8, 1.0 Hz, 6H), 3.93 (s, 9H), 2.71 (s, 9H) ppm. R_f (hexane: ethyl acetate, 8:2): 0.35.

Synthesis of 5'-(4-carboxy-2-methylphenyl)-2,2''-dimethyl-[1,1':3',1''-terpenyl]-4,4''-dicarboxylate (6)

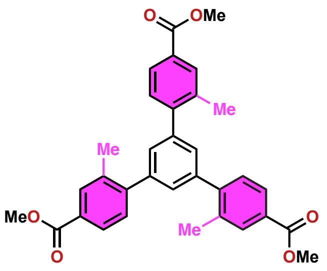

Following the general *procedure A*, reaction of the compound **1** (3.00 g, 6.58 mmol) with methyl-4-bromo-3-methylbenzoate (4.95 g, 21.61 mmol). Purification by column chromatography [silica gel, Hexane/EtOAc (8:2)] afforded 2.00 g, 3.75 mmol (68%) of the title compound **6** as a pale brown solid. ^{1}H NMR (500 MHz, $CDCl_3$) δ: 7.84 (d, *J* = 1.8 Hz, 3H), 7.77 (dd, *J* = 8.0, 1.8 Hz, 3H), 7.22 (d, *J* = 8.0 Hz, 3H), 7.14 (s, 3H), 3.78 (s, 9H), 2.26 (s, 9H) ppm. ^{13}C NMR (126 MHz, DEPT-135, DMSO-d_6) δ: 166.7 (C), 145.5 (C), 140.8 (C), 135.4 (C), 131.4 (CH), 129.7 (CH), 129.0 (C), 128.2 (CH), 126.9 (CH), 51.9 (CH_3), 20.4 (CH_3). HRMS (ESI, m/z) for $C_{30}H_{24}O_6$: Calc. $[M + H]^+$: 523.2115; found: 523.2107. R_f (hexane: ethyl acetate, 8:2): 0.40.

Synthesis of 1,1',1''-Trimethyl 4,4',4''-(1,3,5-benzenetriyl)tris[1-napthalencarboxylate] (7)

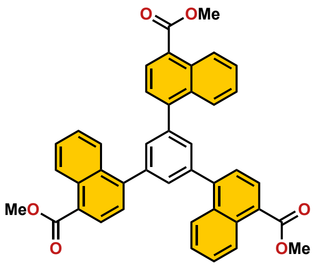

Following the general *procedure A*, reaction of the compound **1** (4.50 g, 9.87 mmol) with methyl 4-bromo-1-naphthoate (3.0 g, 11.71 mmol) under the presence of dioxane as the only solvent. Purification by column chromatography [silica gel, Hexane/EtOAc (8:2)] afforded 3.80 g, 5.92 mmol (60%) of the title compound **7** as a pale grey solid. NMR data match with the previously reported in the literature.[5] ^{1}H NMR (300 MHz, $CDCl_3$) δ: 9.05 – 8.95 (m, 3H), 8.24 (d, *J* = 7.5 Hz, 3H), 8.22 – 8.16 (m, 3H), 7.73 (s, 3H), 7.65 (ddd, *J* = 8.6, 6.9, 1.5 Hz, 3H), 7.62 (d, *J* = 7.5 Hz, 3H), 7.56 (ddd, *J* = 8.3, 6.8, 1.4 Hz, 3H), 4.03 (s, 9H) ppm. R_f (hexane: ethyl acetate, 8:2): 0.38.

Synthesis of trimethyl 2,2',2''-Trimethyl 6,6',6''-(1,3,5-benzenetriyl)tris[2-naphthalenecarboxylate] (8)

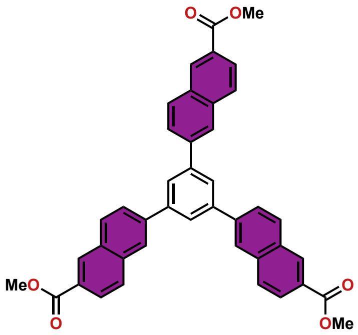

Following the general *procedure A*, reaction of the compound **1** (4.50 g, 9.87 mmol) with methyl 6-bromo-2-naphthoate (3.0 g, 11.71 mmol). Purification by column chromatography [silica gel, Hexane/EtOAc (8:2)] afforded 3.48 g, 5.41 mmol (55%) of the title compound **8** as a pale grey solid. NMR data match with the previously reported in the literature.[6] ^{1}H NMR (300 MHz, $CDCl_3$) δ: 8.67 – 8.64 (m, 3H), 8.21 – 8.18 (m, 3H), 8.11 (dd, *J* = 8.6, 1.7 Hz, 3H), 8.07 (d, *J* = 8.5 Hz, 3H), 8.05 (s, 3H), 7.98 – 7.91 (m, 6H), 4.01 (s, 9H) ppm. R_f (hexane: ethyl acetate, 8:2): 0.48.

Synthesis of dimethyl 10,10'-(5-(3-(methoxycarbonyl)8nthracene-1-yl)-1,3-phenylene)bis(anthracene-9-carboxylate) (9)

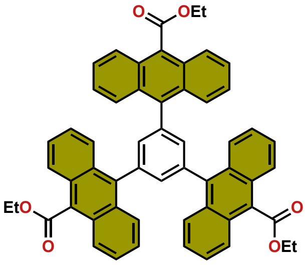

Following the general *procedure A*, reaction of the compound **1** (3.0 g, 6.58 mmol) with ethyl 10-bromoanthracene-9-carboxylate (5.0 g, 15.92 mmol). Purification by column chromatography [silica gel, Hexane/EtOAc (8:2)] afforded 3.76 g, 4.80 mmol (73%) of the title compound **9** as a yellow solid. ^{1}H NMR (300 MHz, $CDCl_3$) δ: 8.22 – 8.11 (m, 6H), 8.11 – 8.02 (m, 6H), 7.72 (s, 3H), 7.64 – 7.50 (m, 12H), 4.70 (q, *J* = 7.1 Hz, 6H), 1.53 (t, *J* = 7.1 Hz, 9H) ppm. ^{13}C NMR (126 MHz, DEPT-135, $CDCl_3$) δ: 169.9 (C), 138.9 (C), 138.5(C), 133.8 (CH), 129.8 (C), 129.2 (C), 128.1 (C), 126.9 (CH), 126.8 (CH), 126.1 (CH), 125.5 (CH), 62.0 (CH2), 14.6 (CH3) ppm. HRMS (ESI, m/z) for $C_{57}H_{42}O_6$: $[M + H]^+$: 823.3054; found: 823.3032. R_f (hexane: ethyl acetate, 8:2): 0.35.

S.2.3. General Procedure B: Saponification

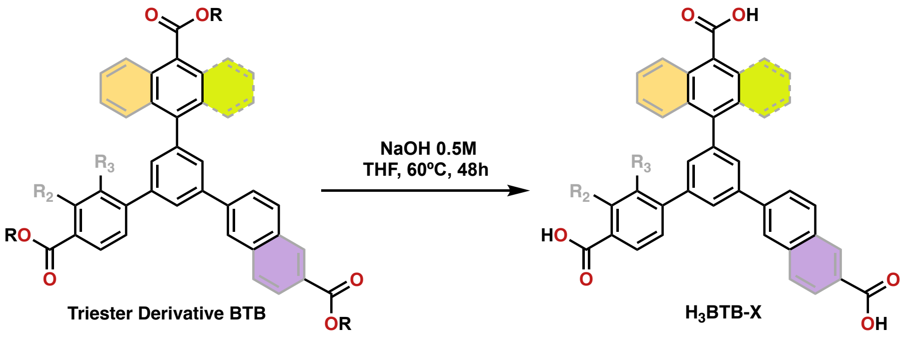

Scheme S3. Syntheses of H_3btb-X and H_3btb-Y based on a classical saponification reaction.

The corresponding triester derivative previously synthesized following the general *procedure A* (5.2 mmol) was dissolved in a 500 mL round bottom flask with 100 mL THF. Subsequently, 100 mL of an aqueous solution 0.5 M of NaOH was added. The suspension was stirred at 60 °C for 48 hours. After cooling down, THF was removed by rotary evaporation. Then, the aqueous solution was acidified with concentrated HCl (37%) to pH < 2. The precipitate was collected by filtration, washed with deionized water, and dried under high vacuum.

Synthesis of 1,3,5-Tris(4-carboxyphenyl) benzene (10, H_3btb)

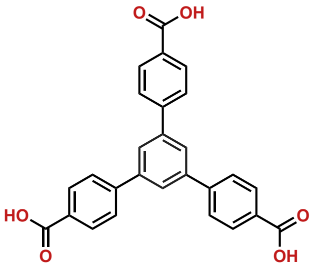

Following the general *procedure B*, saponification of compound **2** (4.00 g, 9.13 mmol) afforded 3.60 g, 8.21 mmol (90%) of the title compound **10 [H_3btb]** as a white solid. NMR data match with the previously reported in the literature.[7] ^{1}H NMR (500 MHz, DMSO-d_6) δ:13.03 (brs, 3H), 8.09 (s, 3H), 8.06 (m, 12H) ppm.

Synthesis of 4,4''-Dimethyl 5'-(4-Carboxy-2-fluorophenyl)-3,3''-difluoro[1,1':3',1''-terphenyl]-4,4''-dicarboxylic acid (11, H_3btb(*o*-F)$_3$)

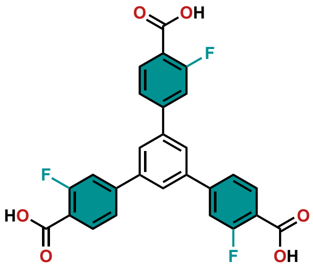

Following the general *procedure B*, saponification of compound **3** (1.85 g, 3.76 mmol) afforded 1.79 g, 3.64 mmol (97%) of the title compound **11 [H_3btb(*o*-F)$_3$]** as a white solid. NMR data match with the previously reported in the literature. [3] ^{1}H NMR (300 MHz, DMSO-d_6) δ: 13.30 (brs, 3H), 8.20 (s, 3H), 8.07 – 7.89 (m, 9H) ppm. ^{19}F NMR (282 MHz, DMSO-d_6) δ: -110.33 (s, 3F) ppm.

Synthesis 4,4''-Dimethyl 3',3''-difluoro-5'-[3-fluoro-4-(methoxycarbonyl)phenyl)-[1,1':3',1''-terphenyl]-4,4''-dicarboxylic acid (12, H_3btb(*m*-F)$_3$)

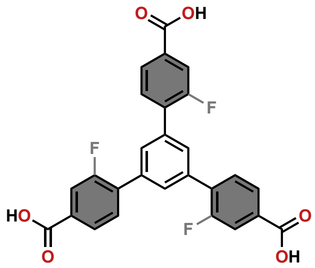

Following the general *procedure B*, saponification of compound **4** (2.05 g, 4.16 mmol) afforded 1.98 g, 4.02 mmol (97%) of the title compound **12 [H_3btb(*m*-F)$_3$]** as a white solid. ^{1}H NMR (300 MHz, DMSO-d_6) δ: 13.38 (brs, 3H), 7.91 – 7.80 (m, 12H) ppm. ^{13}C NMR (75 MHz, DEPT-135, DMSO-d_6) δ: 164.9 (d, J = 3.4 Hz, C), 161.7 (d, J = 256.8 Hz, C-F), 145.6 (d, J = 9.2 Hz, C), 139.4 (C), 132.4 (CH), 126.2 (CH), 123.2 (d, J = 3.3 Hz, CH), 118.3 (d, J = 10.6 Hz, C), 115.7 (d, J = 23.6 Hz, CH) ppm. ^{19}F NMR (282 MHz, DMSO-d_6) δ: -117.79 ppm. HRMS (ESI, m/z) for $C_{27}H_{14}F_3O_6$: Calc. [M – H]$^-$: 491.0742; found: 491.0738.

Synthesis of 5'-(4-Carboxy-3-methylphenyl)-3,3''-dimethyl[1,1':3',1''-terphenyl]-4,4''-dicarboxylic acid (13, H_3btb(*o*-Me)$_3$)

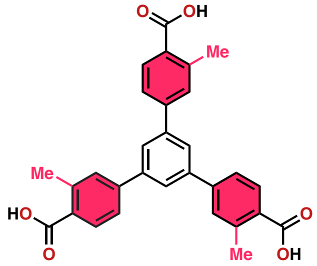

Following the general *procedure B*, saponification of compound **5** (2.75 g, 5.72 mmol) afforded 2.75 g, 5.15 mmol (97%) of the title compound **13 [H_3btb(*o*-Me)$_3$]** as a white solid. NMR data match with the previously reported in the literature. [3] ^{1}H NMR (300 MHz, DMSO-d_6) δ: 12.86 (brs, 3H), 8.04 (s, 3H), 7.96 (d, J = 8.1 Hz, 3H), 7.89 – 7.77 (m, 6H), 2.65 (s, 9H) ppm.

Synthesis of 5'-(4-carboxy-2-methylphenyl)-2,2''-dimethyl-[1,1':3',1''-terphenyl]-4,4''-dicarboxylic acid (14, H_3btb(*m*-Me)$_3$)

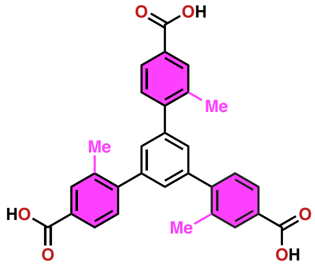

Following the general *procedure B*, saponification of compound **6** (2.00 g, 4.16 mmol) afforded 2.75 g, 5.15 mmol (97%) of the title compound **14 [H_3btb(*m*-Me)$_3$]** as a white solid. ^{1}H NMR (500 MHz, DMSO-d_6) δ: 12.72 (brs, 3H), 7.89 (s, 3H), 7.81 (d, J = 8.1 Hz, 3H), 7.70 (d, J = 1.9 Hz, 3H), 7.67 (dd, J = 8.0, 1.9 Hz, 3H), 2.50 (s, 9H) ppm. ^{13}C NMR (126 MHz, DEPT-135, DMSO-d_6) δ: 168.3 (C), 142.6 (C), 140.5 (C), 139.8 (C), 130.8 (CH), 130.10(CH), 129.4 (C), 125.14 (CH), 124.4 (CH), 21.3 (CH_3) ppm. HRMS (ESI, m/z) for $C_{30}H_{24}O_6$: Calc. [M - H]$^-$:479.1495; found: 479.1504.

Synthesis of 4,4',4''-(1,3,5-benzenetriyl)tris[1-napthalencarboxylic acid] (15, H_3btb(1,4-napth))

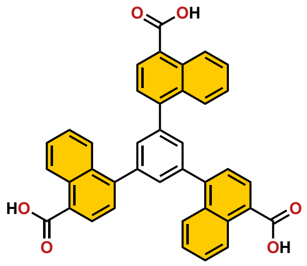

Following the previously reported procedure[5] saponification of compound **7** (3.80 g, 5.92 mmol) was dissolved in a mixture of THF (52.6 mL) and MeOH (52.6 mL). Then 52.6 mL of an aqueous NaOH 6M solution was added. The suspension was stirred under reflux at 60ºC for 48 hours. After removing THF by rotary evaporation, the residue was dissolved in water. The aqueous solution was acidified with concentrated HCl (37%) in an ice-water batch to pH < 2. The white precipitate was collected by filtration, washed with deionized water and dried under vacuum to afford 3.49 g, 5.94 mmol (92%) of the title compound **15** as a white solid. NMR data match with the previously reported in the literature.[5] ^{1}H NMR (300 MHz, DMSO-d_6) δ: 13.25 (brs, 3H), 8.99 – 8.95 (m, 3H), 8.24 – 8.18 (m, 6H), 7.78 (d, J = 7.5 Hz, 3H), 7.75 (s, 3H), 7.72 – 7.64 (m, 6H) ppm.

Synthesis of 6,6',6''-(1,3,5-benzenetriyl)tris[2-naphthalenecarboxylic acid (16, H_3btb(2,6-napth))

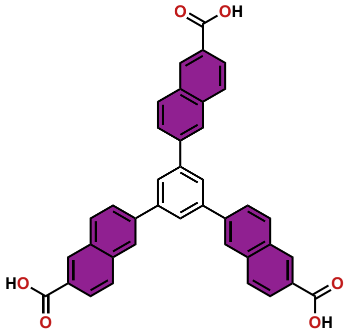

Following the general *procedure B*, saponification of compound **8** (3.48 g, 5.91 mmol) afforded 2.66 g, 5.54 mmol (97%) of the title compound **16** [**H_3btb(2,6-napth)**] as a white solid. NMR data match with the previously reported in the literature. [6] ^{1}H NMR (300 MHz, DMSO-d_6) δ: 13.25 (brs, 3H), 8.67 (ddt, *J* = 16.1, 1.8, 0.8 Hz, 6H), 8.36 (s, 3H), 8.33 – 8.23 (m, 6H), 8.17 (dt, *J* = 9.0, 0.7 Hz, 3H), 8.06 (dd, *J* = 8.6, 1.7 Hz, 3H) ppm.

Synthesis of dimethyl 10,10'-(5-(3-(methoxycarbonyl)anthracen-1-yl)-1,3-phenylene)bis(anthracene-9-carboxylic acid) (17, H_3btb(anth))

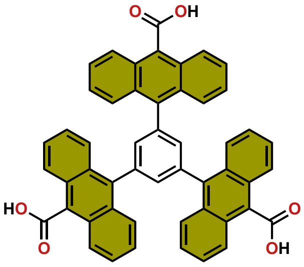

Following the general *procedure B*, saponification of compound **9** (3.76 g, 5.09 mmol) afforded 3.68 g, 4.98 mmol (98%) of the title compound **17** [**H_3btb(anth)**] as a yellow solid. ^{1}H NMR (300 MHz, DMSO-d_6) δ: 14.08 (s, 3H), 8.23 – 8.13 (m, 6H), 8.13 – 8.06 (m, 6H), 7.77 – 7.72 (m, 6H), 7.70 (brs, 3H), 7.69 – 7.63 (m, 6H) ppm. ^{13}C NMR (126 MHz, DEPT-135, DMSO-d_6) δ: 168.8 (C), 138.4 (C), 137.8 (C), 133.0 (CH), 129.0 (CH), 128.9 (C), 127.2 (CH), 127.0 (C), 126.6 (C), 126.6 (CH), 125.0 (CH) ppm. HRMS (ESI, m/z) for $C_{51}H_{30}O_6$: Calc. $[M + NH_4]^+$: 756.2381; found:756.2370.

S.3. SYNTHESIS OF THE MATERIALS

Optimization of synthetic conditions for the formation of MUV-12(X) and MUV-12(Y) materials

For the optimization of the reaction conditions, we made use of a FLEX SHAKE high-throughput workstation from Chemspeed© for robotic dispensing of solids and liquids. The optimization was carried out by screening different reaction parameters like temperature (in an explored range: 100 – 180ºC, with increases of 5ºC at a time), solvent (*N,N*-Dimethylformamide and *N,N*-Diethylformamide), modulator (acetic acid, benzoic acid and trifluoroacetic acid), amount of modulator (from 0.5 to 5.0 mL), concentration (from 0.0083 to 0.037 mM), reaction time (in an explored range: 18 – 96 hours, with increases of 6h at a time) and titanium precursor/source: $Ti(O^{i}Pr)_4$ and $[Ti_6O_6(O^{i}Pr)_6(4\text{-tbbz})_6]$.

NOTE: To minimize the number of factors that can affect the formation of these isoreticular materials, the synthesis of the MUV-12(X) and MUV-12(Y) should be carried out with special attention in the quality of the organic linkers.

After the synthesis of the materials, the single crystals were recovered by centrifugation and rinsed with fresh DMF and acetone several times.

S.3.1. Synthesis of MUV-12

Following a modified procedure reported by our group,[8] the synthesis of MUV-12 solid was carried out in a 25 mL Schott bottle by dissolving 250.0 mg of compound **10** (H_3btb, 0.57 mmol), 14.2 mg of anhydrous $CaCl_2$ (130 μmol) in a mixture of 12 mL of *N,N*-dimethylformamide and 3.5 mL of AcOH. Subsequently, 80 μL of $Ti(O^{i}Pr)_4$ (260 μmol) were added to the clear solution. The bottle was sealed and heated in an oven at 120ºC for 48 hours (heating rate: 5ºC·min^{-1}, cooling rate: 0.2ºC·min^{-1}).

S.3.2. Synthesis of MUV-12(tatb)

The synthesis of MUV-12(tatb) was carried out in a 10 mL PTFE bottle by dissolving 50.0 mg of the commercial compound H_3btb(tatb) (0.103 mmol), 7.2 mg of anhydrous $CaCl_2$ (65 μmol) in a mixture of 3 mL of *N,N*-dimethylformamide and 1 mL of AcOH. Subsequently, 20 μL of $Ti(O^{i}Pr)_4$ (65 μmol) were added to the clear solution. The bottle was sealed and heated in an oven at 120ºC for 48 hours (heating rate: 1ºC·min^{-1}, cooling rate: 0.1ºC·min^{-1}).

S.3.3. Synthesis of MUV-12(OH)

The synthesis of MUV-12(OH) was carried out in a 10 mL PTFE bottle by dissolving 40.0 mg of the commercial compound H_3btb(OH) (0.088 mmol), 7.2 mg of anhydrous $CaCl_2$ (65 μmol) and of 20 μL of $Ti(O^{i}Pr)_4$ (65 μmol) in a mixture of 6 mL of *N,N*-dimethylformamide and 0.5 mL of AcOH. (heating rate: 5ºC·min^{-1}, cooling rate: 0.2ºC·min^{-1}). The bottle was sealed and heated in an oven at 120℃ for 48h hours.

S.3.4. Synthesis of MUV-12(*o*-F)$_3$

The synthesis of MUV-12(*o*-F)$_3$ was carried out in a 25 mL PTFE bottle by dissolving 100.0 mg of compound **11** (H_3btb(*o*-F)$_3$, 0.20 mmol), 18.0 mg of anhydrous $CaCl_2$ (162.5 μmol) in a mixture of 15.0 mL of *N,N*-diethylformamide and 3.0 mL of AcOH. Subsequently, 50.0 μL of $Ti(O^{i}Pr)_4$ (162.5 μmol) were added to the clear solution. The bottle was sealed and heated in an oven at 115ºC for 72 hours (heating rate: 5ºC·min^{-1}, cooling rate: 0.2ºC·min^{-1}).

S.3.4. Synthesis of MUV-12(*m*-F)$_3$

The synthesis of MUV-12(*m*-F)$_3$ was carried out in a 10 mL PTFE bottle by dissolving 40.0 mg of compound **12** (H_3btb(*m*-F)$_3$, 0.08 mmol), 7.2 mg of anhydrous $CaCl_2$ (65 μmol) in a mixture of 6.0 mL of *N,N*-dimethylformamide and 0.5 mL of AcOH. Subsequently, 20.0 μL of Ti(O^{i}Pr)$_4$ (65 μmol) were added to the clear solution. The bottle was sealed and heated in an oven at 120ºC for 48 hours (heating rate: 5ºC·min^{-1}, cooling rate: 0.2ºC·min^{-1}).

S.3.5. Synthesis of MUV-12(*o*-Me)$_3$

The synthesis of MUV-12(*o*-Me)$_3$ was carried out in a 10 mL PTFE bottle by dissolving 40.0 mg of compound **13** (H_3btb(*o*-Me)$_3$, 0.083 mmol), 7.2 mg of anhydrous $CaCl_2$ (65 μmol) in a mixture of 6 mL of *N,N*-dimethylformamide and 0.5 mL of AcOH. Subsequently, 20 μL of Ti(O^{i}Pr)$_4$ (65 μmol) were added to the clear solution. The bottle was sealed and heated in an oven at 120ºC for 48 hours (heating rate: 5ºC·min^{-1}, cooling rate: 0.2ºC·min^{-1}).

S.3.6. Synthesis of MUV-12(*m*-Me)$_3$

The synthesis of MUV-12(*m*-Me)$_3$ was carried out in a 10 mL PTFE bottle by dissolving 40.0 mg of compound **14** (H_3btb(*m*-Me)$_3$, 0.083 mmol), 7.2 mg of anhydrous $CaCl_2$ (65 μmol) in a mixture of 6 mL of *N,N*-dimethylformamide and 0.5 mL of AcOH. Subsequently, 20 μL of Ti(O^{i}Pr)$_4$ (65 μmol) were added to the clear solution. The bottle was sealed and heated in an oven at 120ºC for 48 hours (heating rate: 5ºC· min^{-1}, cooling rate: 0.2ºC·min^{-1}).

S.3.7. Synthesis of MUV-12(1,4-napth)

The synthesis of MUV-12(1,4-napth) was carried out in a 10 mL PTFE bottle by dissolving 40.0 mg of compound **15** (H_3btb(1,4-napth), 0.085 mmol), 7.2 mg of anhydrous $CaCl_2$ (65 μmol) and 20.0 mg of Ti_6 cluster (11.1 mol) in a mixture of 6 mL of *N,N*-dimethylformamide and 0.5 mL of AcOH. The bottle was sealed and heated in an oven at 120ºC for 24h hours (heating rate: 5ºC·min^{-1}, cooling rate: 0.2ºC·min^{-1}).

S.3.8. Synthesis of MUV-12(2,6-napth)

The synthesis of MUV-12(2,6-Napth) was carried out in a 10 mL PTFE bottle by dissolving 40.0 mg of compound **16** (H_3btb(2,6-napth),0.068 mmol), 7.2 mg of anhydrous $CaCl_2$ (65 μmol) in a mixture of 6 mL of *N,N*-diethylformamide and 0.4 mL of AcOH. Subsequently, 20.0 μL of Ti(O^{i}Pr)$_4$ (65 μmol) were added to the clear solution. The bottle was sealed and heated in a preheated oven at 120ºC for 48 hours.

S.3.9. Synthesis of MUV-12(anth)

The synthesis of MUV-12(anth) was carried out in a 10 mL PTFE bottle by dissolving 40.0 mg of compound **17** (H_3btb(anth), 0.054 mmol), 7.2 mg of anhydrous $CaCl_2$ (65 μmol) in a mixture of 6 mL of *N,N*-dimethylformamide and 0.5 mL of AcOH. Subsequently, 20 μL of Ti(O^{i}Pr)$_4$ (65 μmol) were added to the clear solution. The bottle was sealed and heated in an oven at 120ºC for 48 hours (heating rate: 5ºC·min^{-1}, cooling rate: 0.2ºC·min^{-1}).

S.4. X- RAY DIFFRACTION (XRD)

S.4.1. Single Crystal X-Ray Diffraction

X-ray diffraction data single crystal was collected with synchrotron radiation at ALBA Synchrotron (BL13-XALOC) beamline. The diffraction pattern was indexed using the images taken from a Dectris Pilatus 6M detector with the program CrysAlisPro 1.171.42.63a (Rigaku OD, 2022) obtaining a maximum resolution of 1.00 Å (Q = 21.38°). Data reduction, scaling and absorption corrections were also performed using CrysAlisPro 1.171.42.63a. The structure was solved, and the space group *Pm*-3 (# 200) determined by the ShelXT 2018/2 (Sheldrick, 2018) structure solution program using dual methods and refined by full matrix least squares minimisation on $\boldsymbol{F}^2$ using **olex2.refine** 1.5.[9] All non-hydrogen atoms were refined anisotropically. Hydrogen atom positions were calculated geometrically and refined using the riding model. In this particular case, both the organic part and the Ti/Ca core present a certain degree of disorder that could be modelled. Unfortunately, this has not been the case for the existing solvent molecules in the structure, which could not be modelled due to their high degree of disorder, so the masking procedure existing in Olex2 had to be used.[9–11]

Table S1. Crystallographic Information of MUV-12(anth)
CCDC 2270773

**Compound**	**MUV12(anth)**
Formula	$C_{67}H_{35}CaO_{11}Ti$
$D_{calc.}$/ g cm^{-3}	0.617
m/mm^{-1}	0.154
Formula Weight	1103.963
Colour	colourless
Shape	prism-shaped
Size/mm^3	0.04×0.02×0.02
T/K	100(2)
Crystal System	cubic
Space Group	*Pm*-3
a/Å	26.1272(6)
b/Å	26.1272(6)
c/Å	26.1272(6)
a/°	90
b/°	90
g/°	90
V/Å^3	17835.2(7)
Z	6
Z'	0.25
Wavelength/Å	0.72931
Radiation type	synchrotron
Q_{min}/°	1.79
Q_{max}/°	21.38
Measured Refl's.	30429
Indep't Refl's	3385
Refl's I≥2 *s*(I)	2605
R_{int}	0.0427
Parameters	95
Restraints	121
Largest Peak	1.4491
Deepest Hole	-0.7141
GooF	3.1182
wR_2 (all data)	0.6278
wR_2	0.6009
R_1 (all data)	0.2513
R_1	0.2348

S.4.2. Le Bail & Rietveld Refinements

Rietveld refinements were carried out using TOPAS Academic v6 software (http://www.topas-academic.net/)[2] Prior to Rietveld refinement, a full profile powder refinement was carried out with the LeBail method in all the samples to confirm the space group and phase purity. For the structural refinements with the Rietveld method, we used the reported single-crystal structure of MUV-12 as a starting model. In the case of MUV-12(anth), the starting model used was that obtained by SCXRD. In this case, given the low diffraction at higher angles, only the atomic positions of the Ti, Ca and O atoms in the Ti_2Ca_2 unit were refined. For the rest of MUV-12 structure, the organic linkers were refined as rigid bodies, where the initial bond distance values were set as 1.38 Å (aromatic C-C), 1.46 Å (exocyclic C-C), 1.28 Å (carboxylic C-O). H atoms were placed in ideal positions with a bond distance constrain of 0.93 (C-H) in the final stages of the refinement. All the constrained bond distances were allowed to refine with the exception of the C-H bond with was fixed throughout the refinement. The residual electron density inside the pores was modelled as water molecules, whose position and occupancies were allowed to refine. The background was fitted with a 24-coefficient Chebyshev polynomial and peak-shapes were modelled with a Thompson-Cox-Hasting pseudo-Voigt profile function. The instrumental parameters were obtained from the LeBail refinement of the pristine MUV-12 and were used as a reference for the refinement of the remaining samples.

Table S2. Crystallographic Information of MUV-12(tatb) and MUV-12(OH)

Name	MUV-12(tatb)	MUV-12(OH)
**Empirical formula**	$Ti_3Ca_3C_{98.6}H_{60.3}N_{11.3}O_{87.3}$	$Ti_3Ca_3C_{102.8}H_{49.5}O_{71.3}$
**Formula weight, g mol^{-1}**	3064	2690
**Temperature, K**	298	298
**Crystal system**	Cubic	Cubic
**Space group**	Im-3	Im-3
***a*, Å**	26.1313(3)	26.4030(4)
**Volume, Å^3**	17843.6(5)	18405.9(9)
**Z**	4	4
**Wavelength, Å**	1.540596	1.540596
**Number of structural variables/Number of total variables**	41/62	69/94
**2θ range, °**	3.0 – 70.0	3.0 – 70.0
**R_p, %**	4.02	3.35
**R_{wp}, %**	6.12	5.10
**R_{exp}, %**	1.00	1.15
**R_{Bragg}, %**	2.72	2.02
**GoF**	6.15	4.43

Table S3. Crystallographic Information of MUV-12(*o*-Me)$_3$ and MUV-12(*m*-Me)$_3$

Name	MUV-12(*o*-Me)$_3$	MUV-12(*m*-Me)$_3$
**Empirical formula**	$Ti_3Ca_3C_{111.3}H_{66.1}O_{78.5}$	$Ti_3Ca_3C_{120}H_{84}O_{68.6}$
**Formula weight, g mol^{-1}**	2920	2890
**Temperature, K**	298	298
**Crystal system**	Cubic	Cubic
**Space group**	Im-3	Im-3
***a*, Å**	26.5002(4)	26.4576(4)
**Volume, Å^3**	18610.1(8)	18420.5(8)
**Z**	4	4
**Wavelength, Å**	1.540596	1.540596
**Number of structural variables/Number of total variables**	84/109	72/97
**2θ range, °**	3.0 – 70.0	3.0 – 70.0
**R_p, %**	4.58	4.32
**R_{wp}, %**	6.94	6.78
**R_{exp}, %**	1.12	1.20
**R_{Bragg}, %**	5.12	2.41
**GoF**	6.19	5.65

Table S4. Crystallographic Information of MUV-12(o-F)$_3$ and MUV-12(m-F)$_3$

**Name**	**MUV-12(o-F)$_3$**	**MUV-12(m-F)$_3$**
**Empirical formula**	$Ti_3Ca_3C_{108}F_{12}H_{36}O_{65.9}$	$Ti_3Ca_3C_{120}F_{12}H_{36}O_{64.4}$
**Formula weight, g mol^{-1}**	2903	2855
**Temperature, K**	298	298
**Crystal system**	Cubic	Cubic
**Space group**	Im-3	Im-3
***a*, Å**	26.5323(4)	26.4648(3)
**Volume, Å^3**	18677.8(8)	18535.5(5)
**Z**	4	4
**Wavelength, Å**	1.540596	1.540596
**Number of structural variables/Number of total variables**	74/99	74/99
**2θ range, °**	3.0 – 70.0	3.0 – 70.0
**R_p, %**	3.79	2.93
**R_{wp}, %**	5.78	4.04
**R_{exp}, %**	0.96	0.96
**R_{Bragg}, %**	1.99	1.13
**GoF**	6.00	4.22

Table S5. Crystallographic Information of MUV-12(1,4-naph), MUV-12(2,6-naph), and MUV-12(anth)

Name	MUV-12(1,4-naph)	MUV-12(2,6-naph)	MUV-12(anth)
**Empirical formula**	$Ti_3Ca_3C_{125.9}H_{67.9}O_{97.3}$	-	$Ti_3Ca_3C_{201}H_{105}O_{58.8}$
**Formula weight, g mol^{-1}**	3400	-	3725
**Temperature, K**	298	298	298
**Crystal system**	Cubic	Cubic	Cubic
**Space group**	Pm-3	Im-3 / Pm-3	Pm-3
***a*, Å**	26.448(1)	31.771(3) / 31.760(3)	26.253(1)
**Volume, Å^3**	18501(2)	32070(9) / 32037(8)	18095(3)
**Z**	2	4	2
**Wavelength, Å**	1.540596	1.540596	1.540596
**2θ range, °**	2.0 – 40.0	2.0 – 40.0	2.5 – 40.0
**Weight fraction, %**	-	69.0(8) / 31.0(8)	-
**Number of structural variables/Number of total variables**	46/65	48/90	19/38
**R_p, %**	7.01	6.27	3.85
**R_{wp}, %**	8.86	8.99	5.75
**R_{exp}, %**	0.55	0.73	0.84
**R_{Bragg}, %**	1.11	3.47 / 4.55	1.03
**GoF**	16.26	12.31	6.84

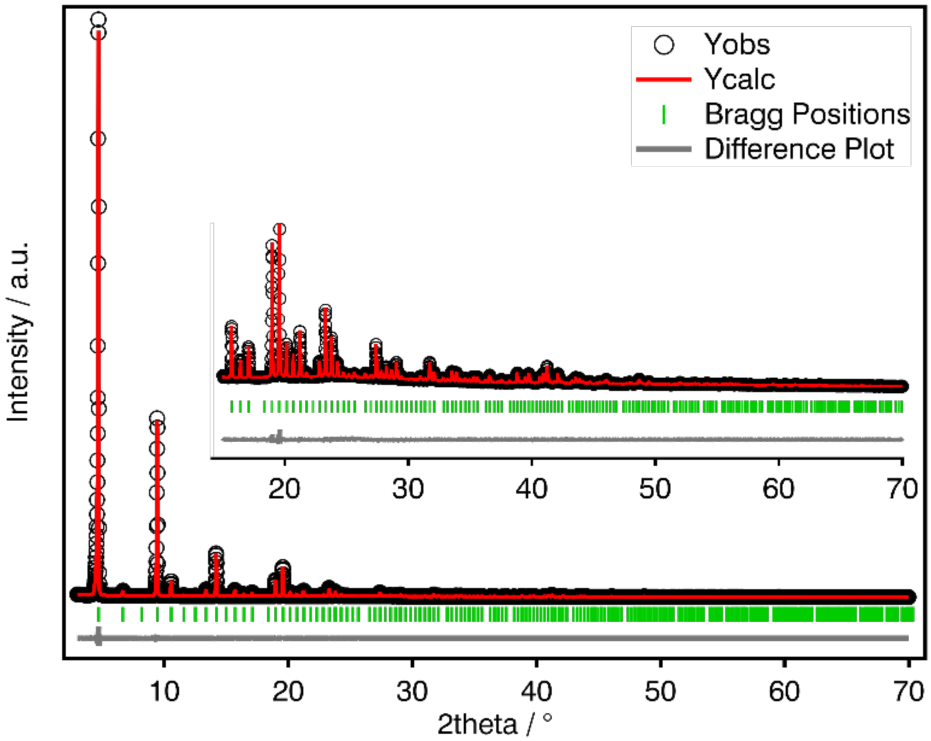

Figure S1. Experimental (black dots), calculated (red line), difference plot [(Iobs−Icalc)] (grey line, bottom panel) and Bragg positions (green ticks, bottom panel) for the Le Bail refinement of the experimental diffraction data of **MUV-12**

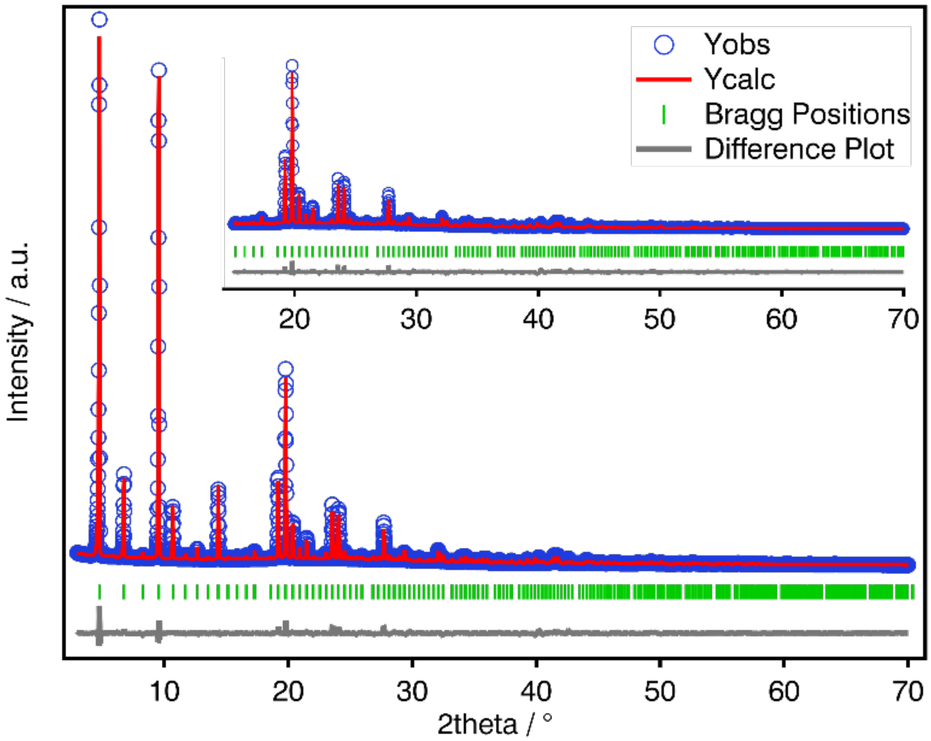

Figure S2. Experimental (blue dots), calculated (red line), difference plot [(Iobs−Icalc)] (grey line, bottom panel) and Bragg positions (green ticks, bottom panel) for the Rietveld refinement of the experimental diffraction data of **MUV-12(tatb)**

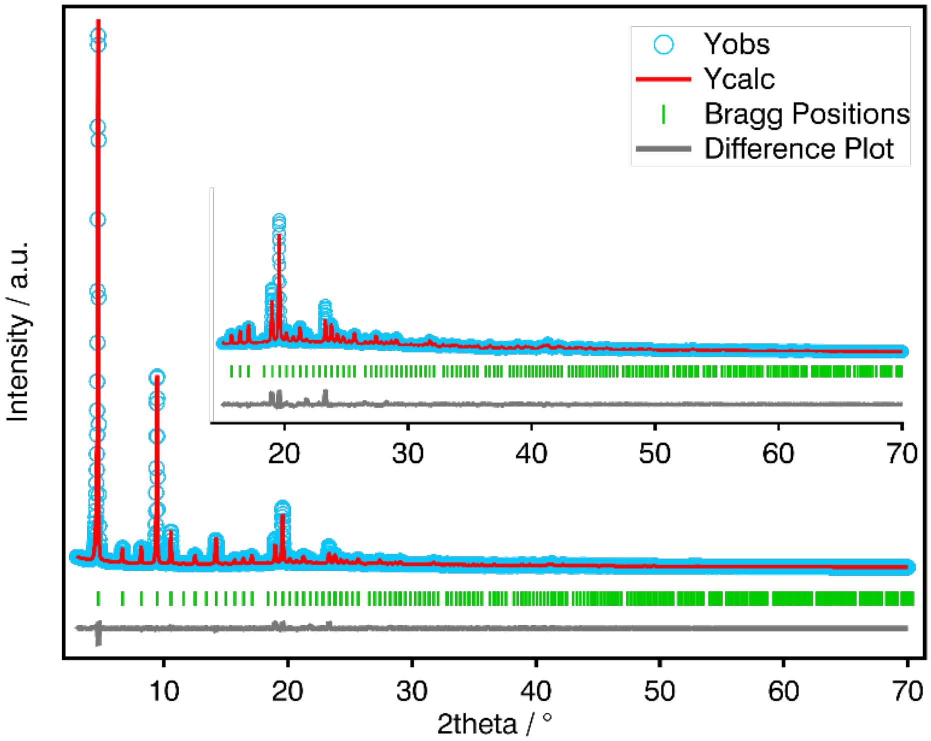

Figure S3. Experimental (blue dots), calculated (red line), difference plot [(Iobs−Icalc)] (grey line, bottom panel) and Bragg positions (green ticks, bottom panel) for the Rietveld refinement of the experimental diffraction data of **MUV-12(OH)**

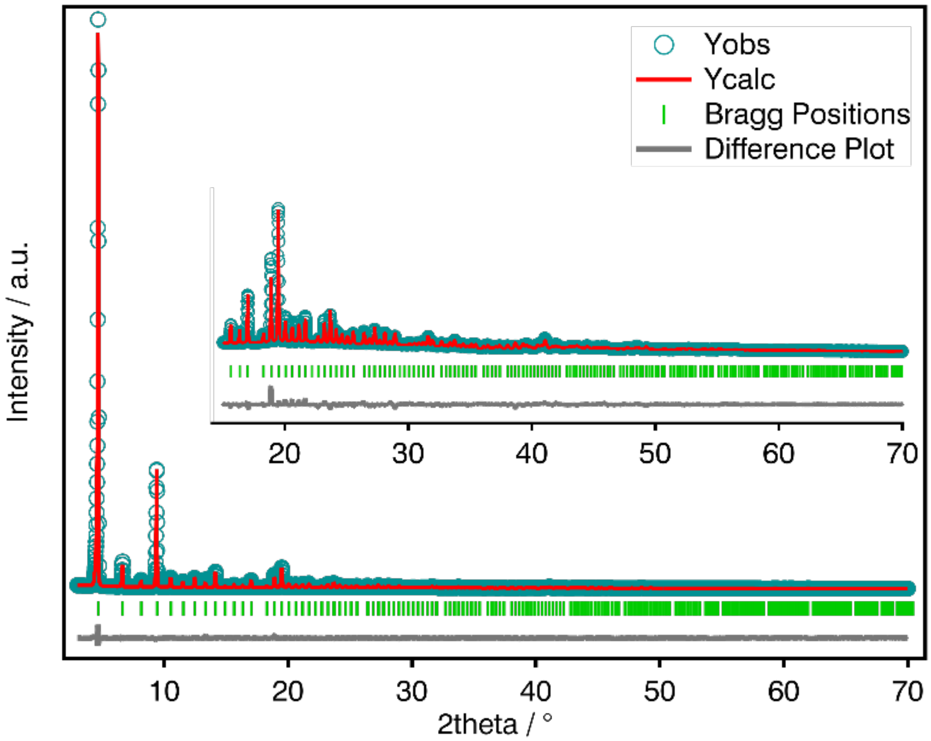

Figure S4. Experimental (blue dots), calculated (red line), difference plot [(Iobs−Icalc)] (grey line, bottom panel) and Bragg positions (green ticks, bottom panel) for the Rietveld refinement of the experimental diffraction data of **MUV-12(*o*-F)$_3$**

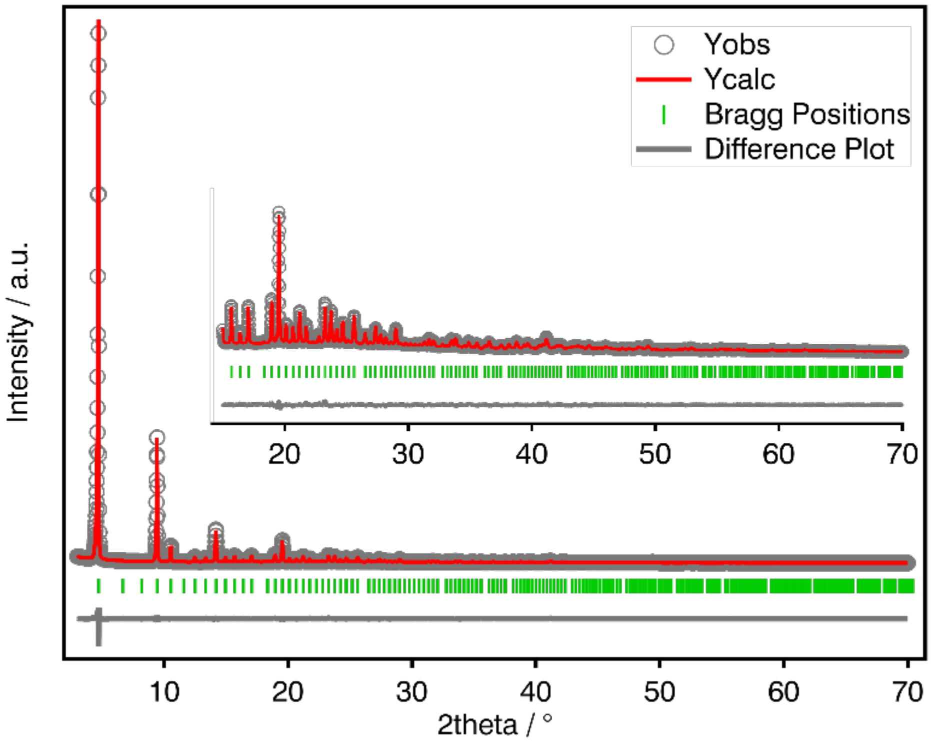

Figure S5. Experimental (grey dots), calculated (red line), difference plot [(Iobs−Icalc)] (grey line, bottom panel) and Bragg positions (green ticks, bottom panel) for the Rietveld refinement of the experimental diffraction data of **MUV-12(*m*-F)$_3$**

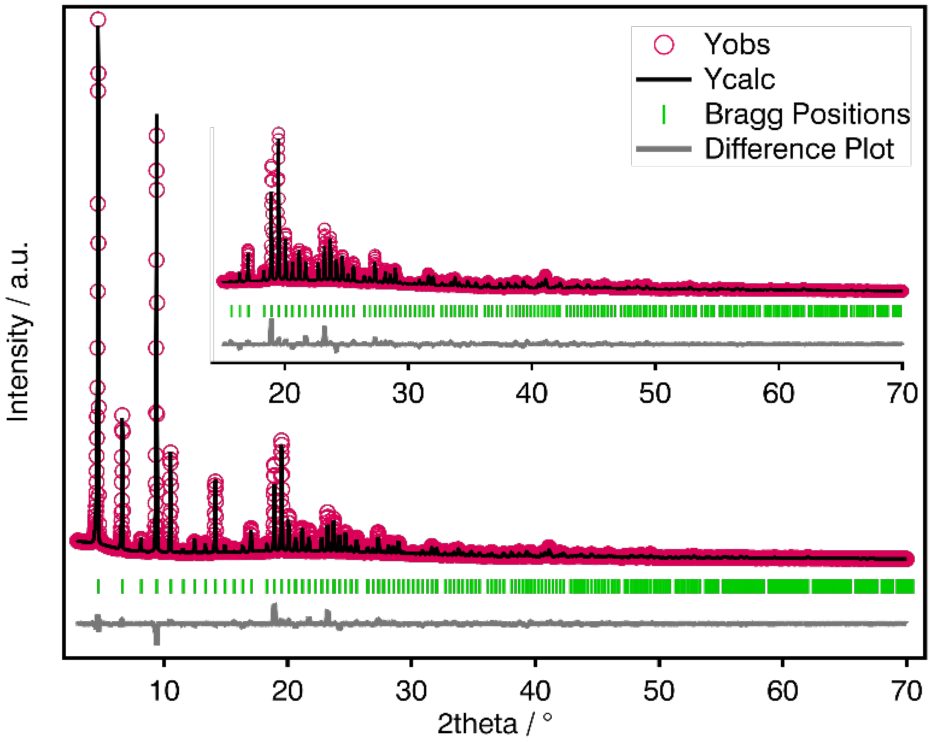

Figure S6. Experimental (magenta dots), calculated (black line), difference plot [(Iobs−Icalc)] (black line, bottom panel) and Bragg positions (green ticks, bottom panel) for the Rietveld refinement of experimental diffraction data of **MUV-12(*o*-Me)$_3$**

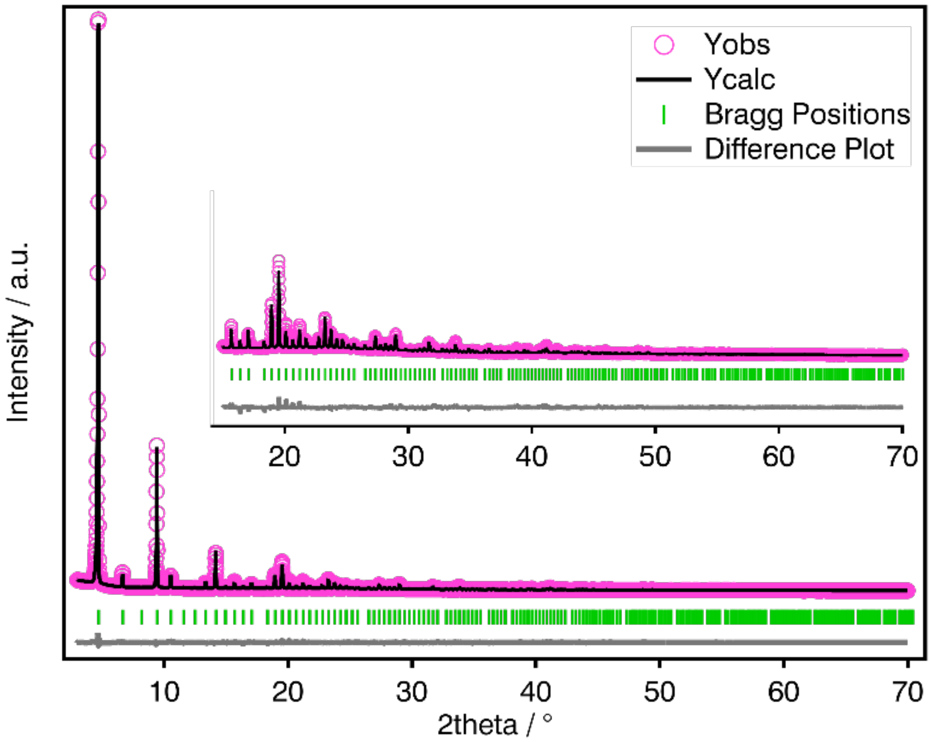

Figure S7. Experimental (pink dots), calculated (black line), difference plot [(Iobs−Icalc)] (black line, bottom panel) and Bragg positions (green ticks, bottom panel) for the Rietveld refinement of experimental diffraction data of **MUV-12(*m*-Me)$_3$**

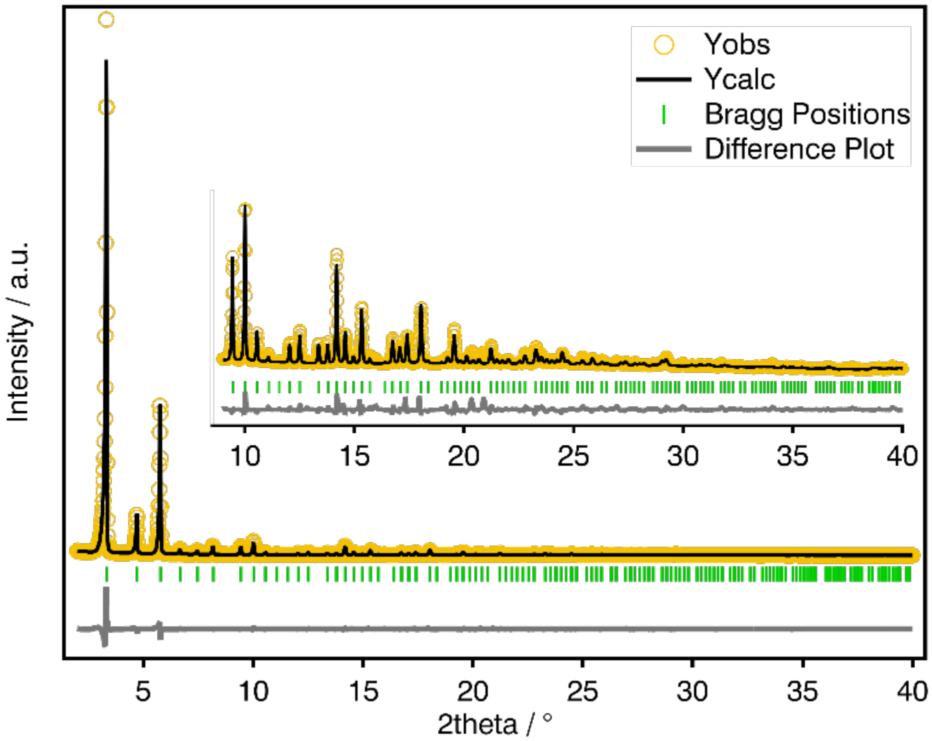

Figure S8. Experimental (yellow dots), calculated (black line), difference plot [(Iobs−Icalc)] (black line, bottom panel) and Bragg positions (green ticks, bottom panel) for the Rietveld refinement of the experimental diffraction data of **MUV-12(1,4-napth)**

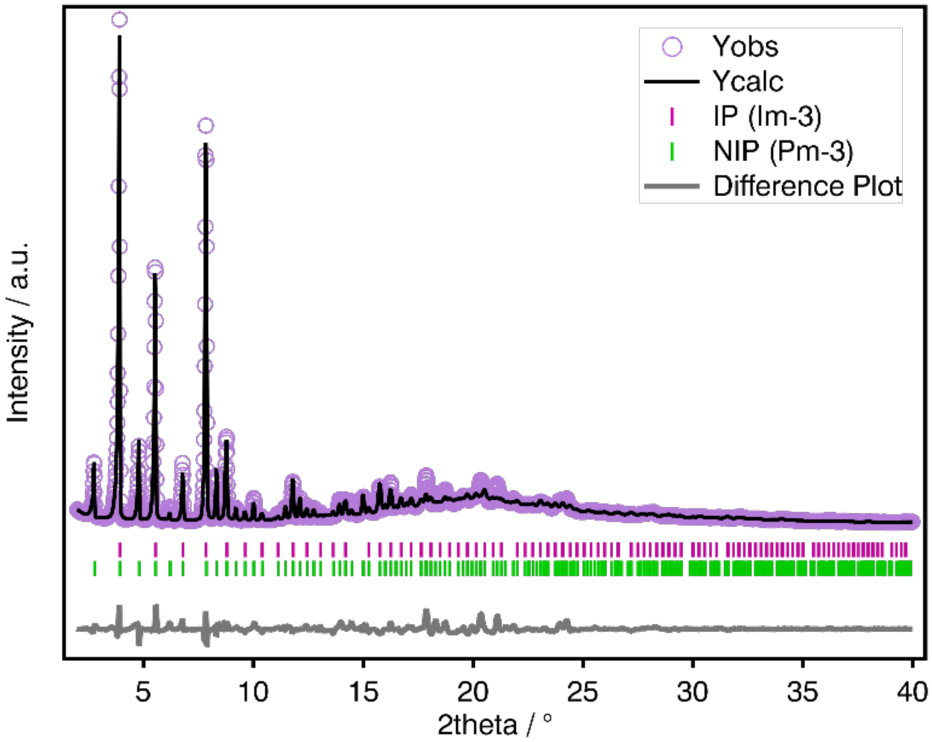

Figure S9. Experimental (purple dots), calculated (blue line), difference plot [(Iobs−Icalc)] (black line, bottom panel) and Bragg positions (green ticks, bottom panel) for the 2-phase Rietveld refinement of experimental diffraction data of **MUV-12(2,6-napth).** The relative weight fractions for the IP and NIP phases are 69.0(8) and 31.0(8) %, respectively.

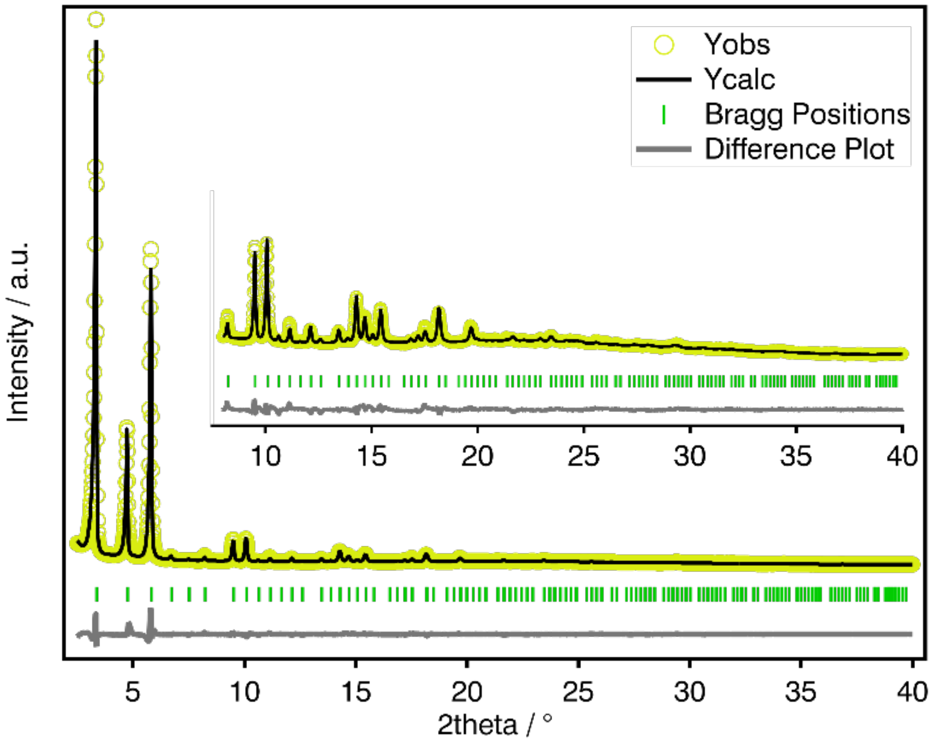

Figure S10. Experimental (green dots), calculated (black line), difference plot [(Iobs−Icalc)] (grey line, bottom panel) and Bragg positions (green ticks, bottom panel) for the Rietveld refinement of the experimental diffraction data of **MUV-12(anth)**

S.5. OPTICAL IMAGES

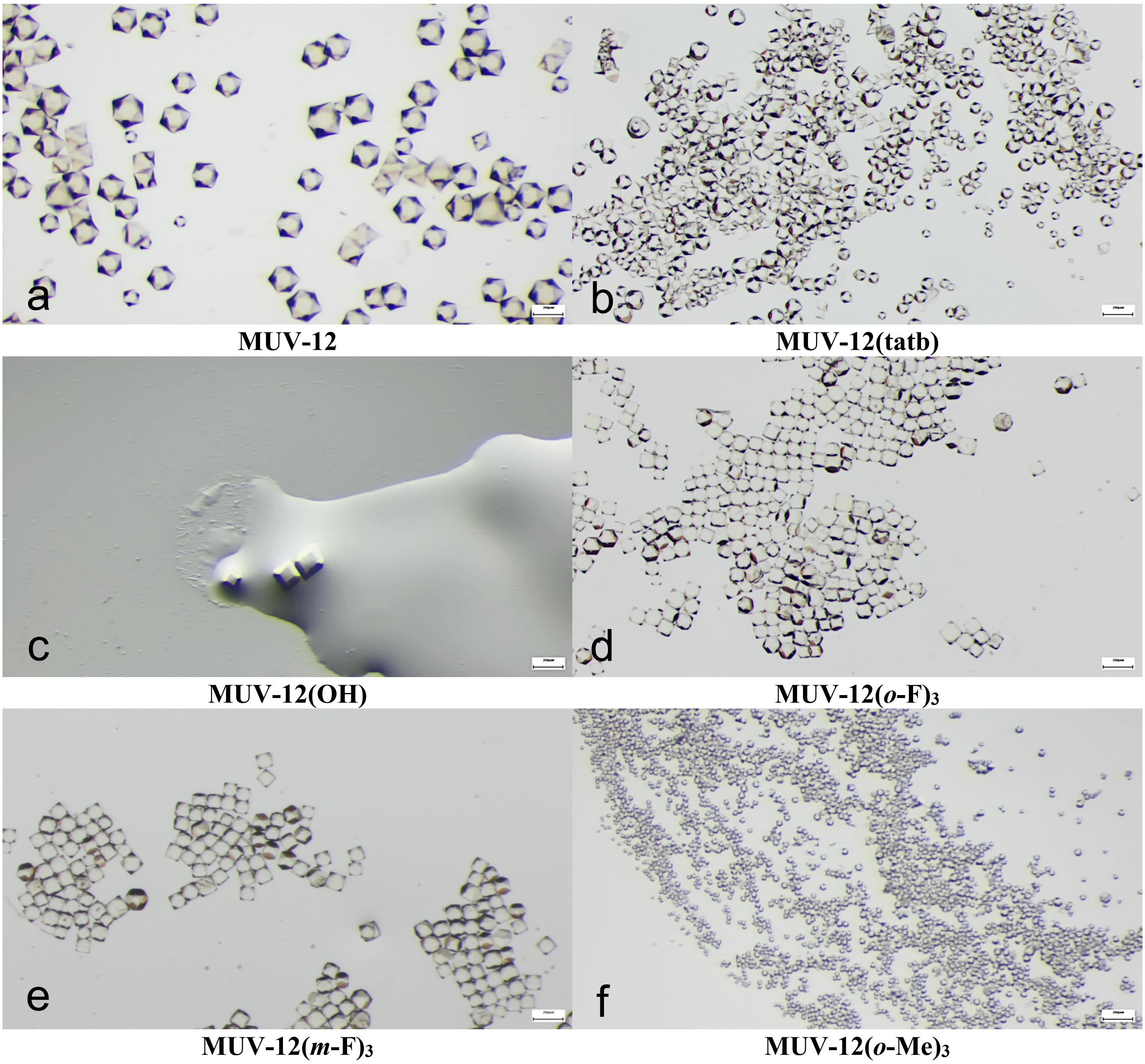

Figure S11. Optical images of MUV-12(X) materials: *a)* MUV-12, *b)* MUV-12(tatb), *c)* MUV-12(OH), *d)* MUV-12(*o*-F)$_3$, *e)* MUV-12(*m*-F)$_3$ and *f)* MUV-12(*o*-Me)$_3$

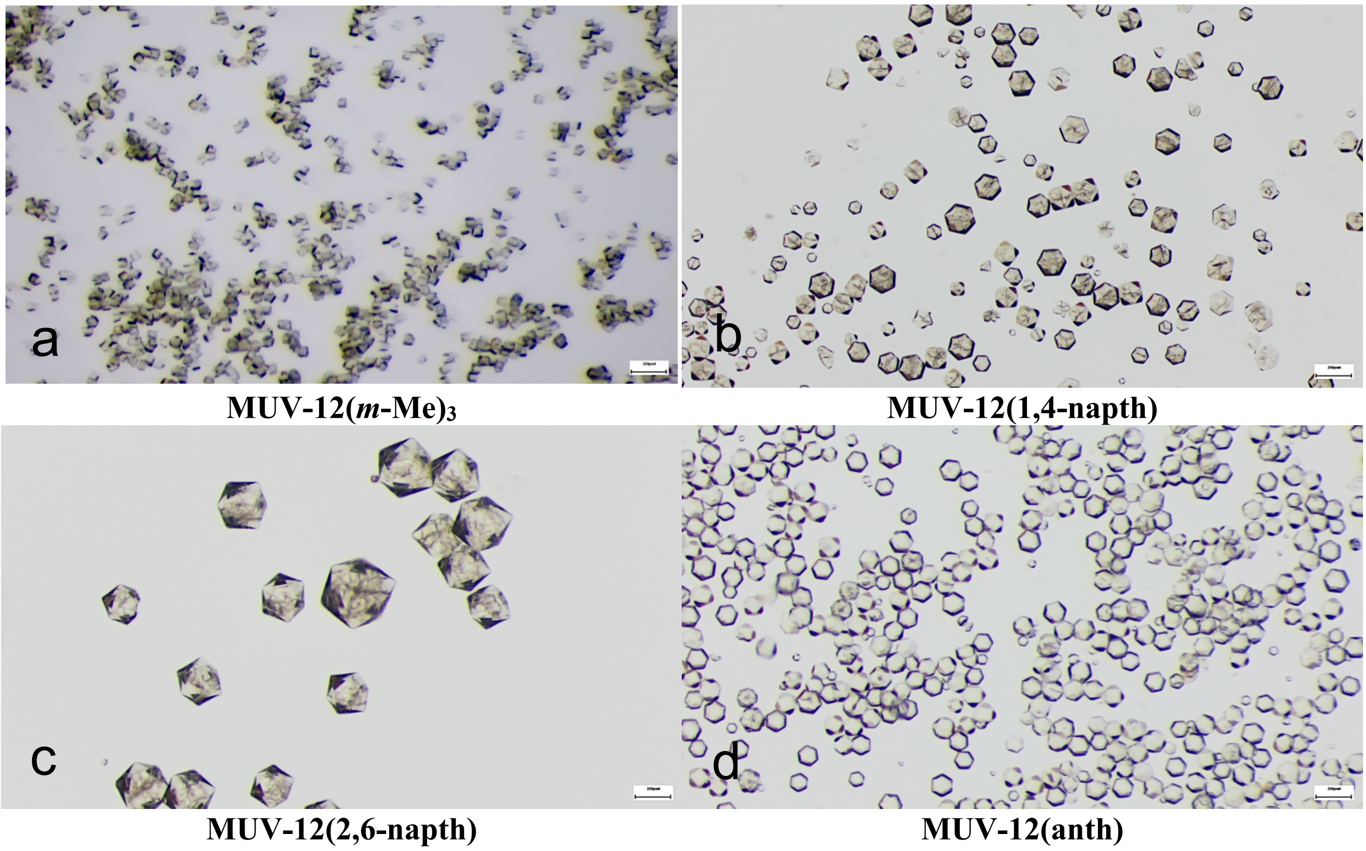

Figure S12. Optical images of MUV-12(X) and MUV-12(Y) materials: *a)* MUV-12(m-Me)$_3$, *b)* MUV-12(1,4-napth), *c)* MUV-12(2,6-napth) and d) MUV-12(anth)

S.6. CHARACTERIZATION OF THE MATERIALS

S.6.1. Analysis of N_2 Adsorption/Desorption Isotherms at 77 K

Gas adsorption measurements were performed ex-situ on MUV-12(X) and MUV-12(Y) solids exchanged in acetone. Surface area, pore size and volume values were calculated from nitrogen adsorption-desorption isotherms (77 K) recorded on a *Micromeritics 3Flex* apparatus. Samples were degassed overnight at 100ºC and 10^{-6} Torr prior to analysis. Brunauer-Emmett-Teller (BET) Surface area analysis were performed as recommended for microporous and mesoporous materials (see below).[12] Specific surface area (SA) was calculated by multi-point Brunauer-Emmett-Teller (BET) method. Total pore volume was taken at P/P_0=0.96. Pore size distribution was analysed by using the solid density functional theory (SWDFT) for the adsorption branch by assuming a cylindrical pore model in the case of MUV-12(X) and for MUV-12(Y) by HS-2D-NLDFT Carb Cyl Mesopores.

a)

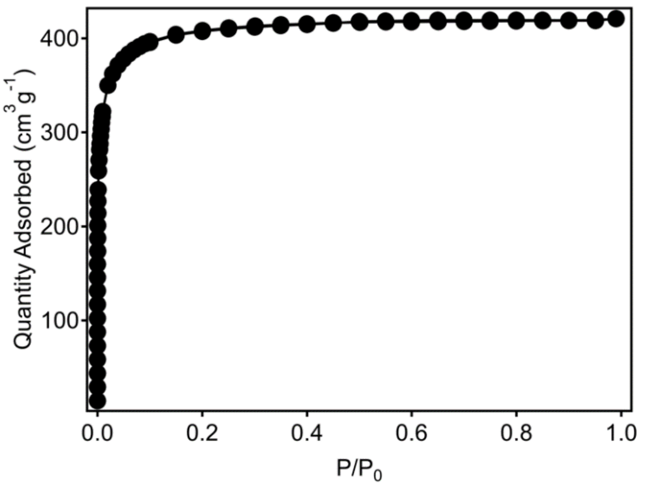

b)

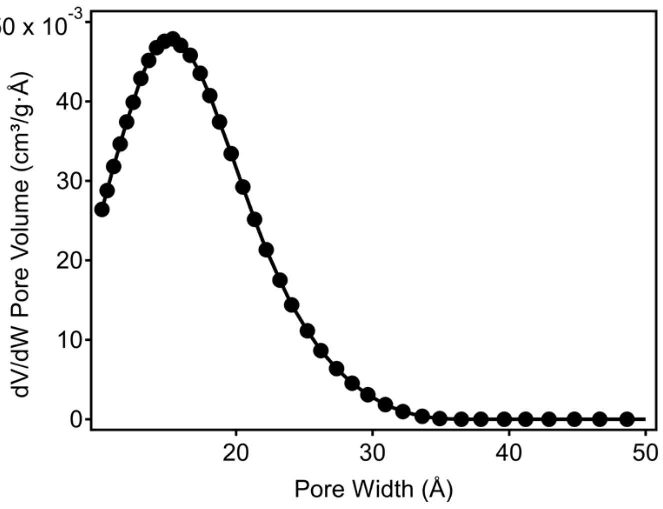

c)

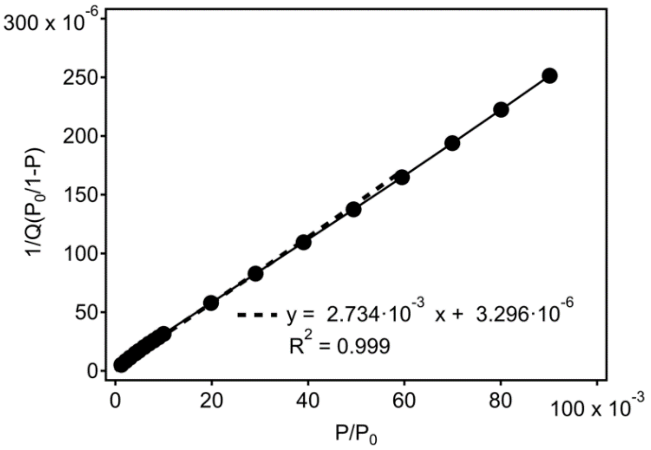

d)

**BET Area (m^2 g^{-1})**	1589.9088 ± 4.0011
**Slope (g/cm^3)**	0.002734 ± 0.000007
**Intercept (g/cm^3)**	0.000003 ± 0.000000
**R^2**	0.9999493
**C**	830.610
**V_m (cm^3 g^{-1})**	365.279
**$1/(\sqrt{C}+1)$**	0.03353

Figure S13. Analysis of the N_2 adsorption/desorption isotherm of **MUV-12** at 77 K. a) N_2 adsorption isotherm; b) Pore Size Distribution calculated by SWNT-NLDFT (regularization = 1.0); c) Multi-Point BET analysis and; d) main parameters calculated from the multi-point BET analysis

a) b)

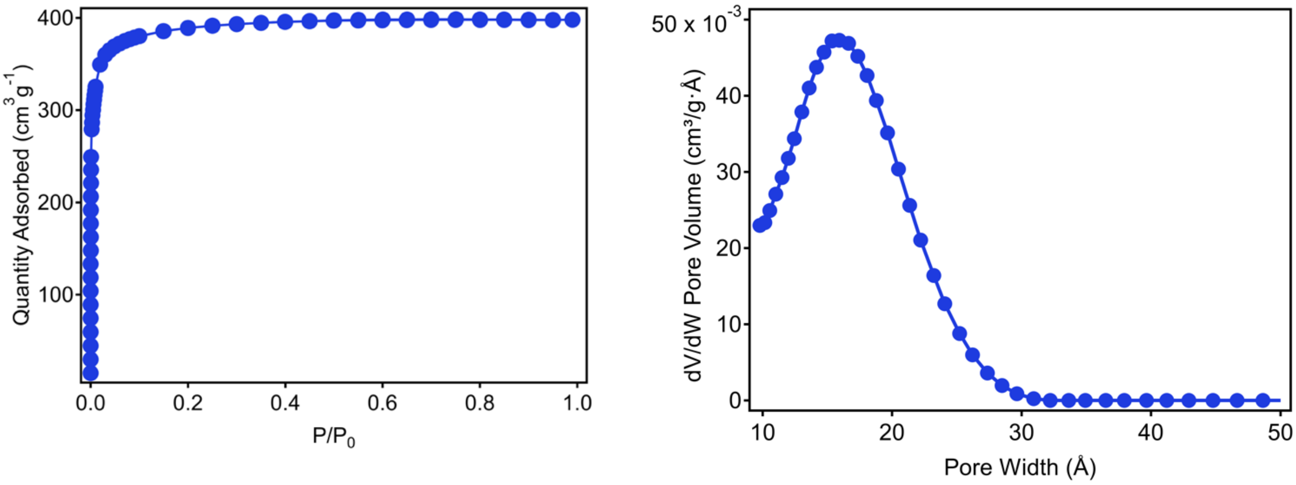

c)

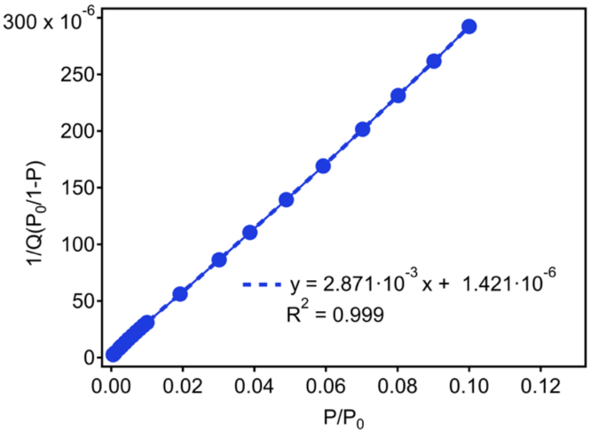

d)

**BET Area (m^2 g^{-1})**	1514.9458 ± 5.2752
**Slope (g/cm^3)**	0.002872 ± 0.000010
**Intercept (g/cm^3)**	0.000001 ± 0.000000
**R^2**	0.999878
**C**	2022.194
**V_m (cm^3 g^{-1})**	348.0571
**$1/(\sqrt{C}+1)$**	0.021754

Figure S14. Analysis of the N_2 adsorption/desorption isotherm of **MUV-12(tatb)** at 77 K. a) N_2 adsorption isotherm; b) Pore Size Distribution calculated by SWNT-NLDFT (regularization = 1.0); c) Multi-Point BET analysis and; d) main parameters calculated from the multi-point BET analysis

a)

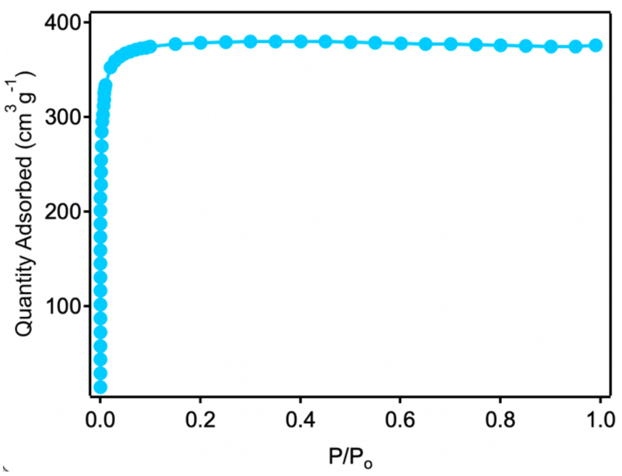

b)

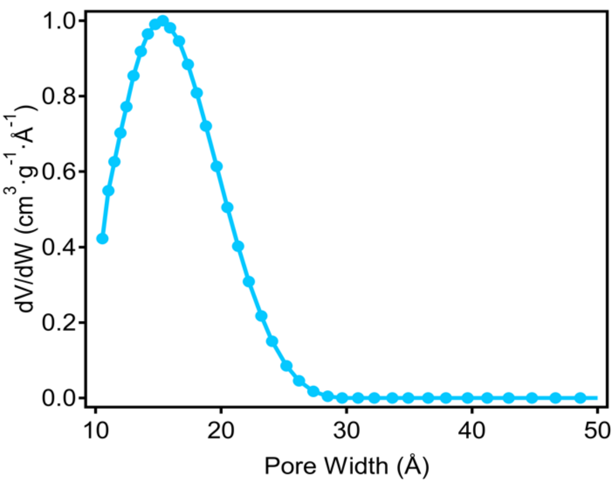

c)

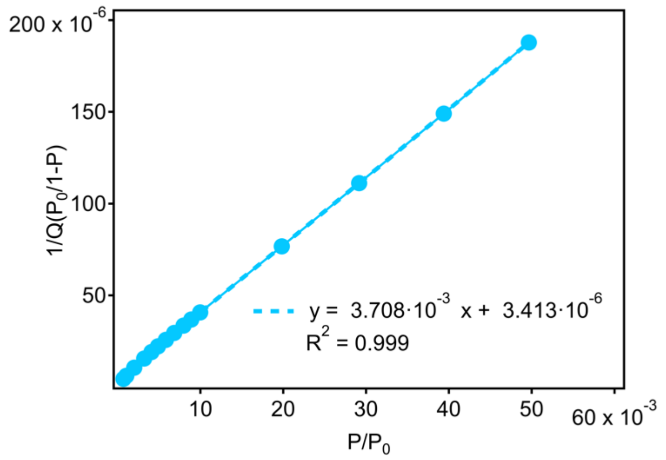

d)

**BET Area (m^2 g^{-1})**	1493.524 ± 4.0863
**Slope (g/cm^3)**	0.003708 ± 0.000013
**Intercept (g/cm^3)**	0.000003 ± 0.000000
**R^2**	0.999210
**C**	1087.506
**V_m (cm^3 g^{-1})**	269.4010
**1/(√C+1)**	0.029431

Figure S15. Analysis of the N_2 adsorption/desorption isotherm of **MUV-12(OH)** at 77 K. a) N_2 adsorption isotherm; b) Pore Size Distribution calculated by SWNT-NLDFT (regularization = 1.0); c) Multi-Point BET analysis and; d) main parameters calculated from the multi-point BET analysis

a)

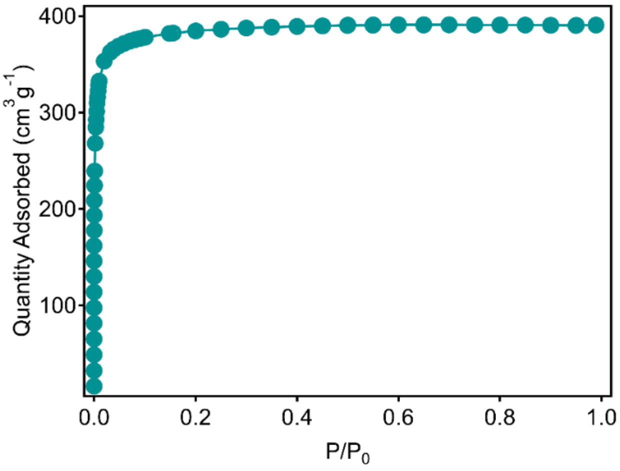

b)

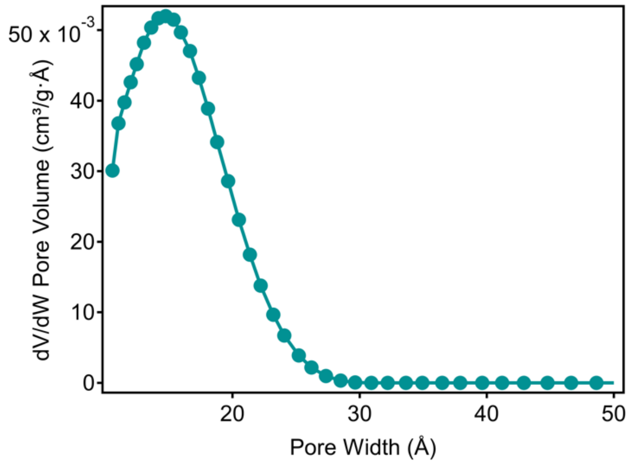

c)

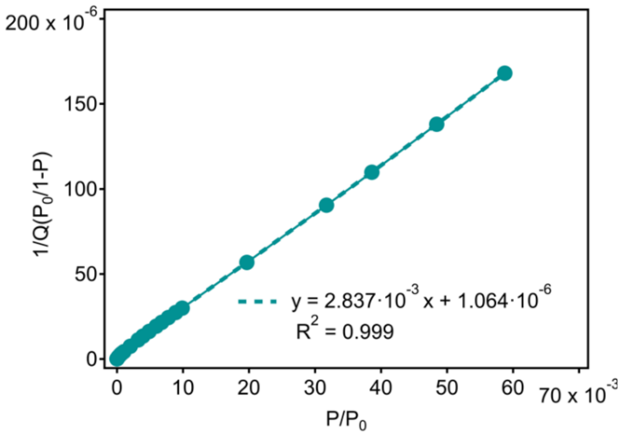

d)

**BET Area (m^2 g^{-1})**	1533.5922 ± 5.9294
**Slope (g/cm^3)**	0.002837 ± 0.000011
**Intercept (g/cm^3)**	0.000001 ± 0.000000
**R^2**	0.99979
**C**	2668.051
**V_m (cm^3 g^{-1})**	352.341
**$1/(\sqrt{C}+1)$**	0.01899

Figure S16. Analysis of the N_2 adsorption/desorption isotherm of **MUV-12(*o*-F)$_3$** at 77 K. a) N_2 adsorption isotherm; b) Pore Size Distribution calculated by SWNT-NLDFT (regularization = 1.0); c) Multi-Point BET analysis and; d) main parameters calculated from the multi-point BET analysis

a)

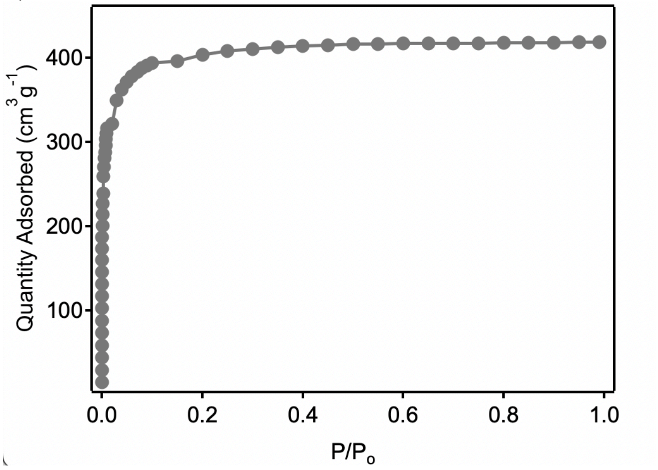

b)

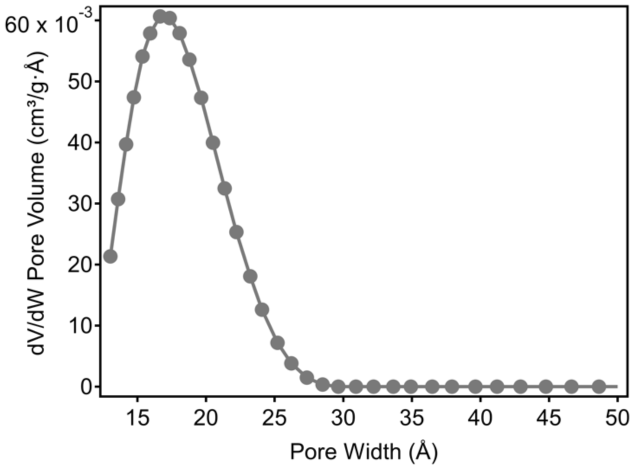

c)

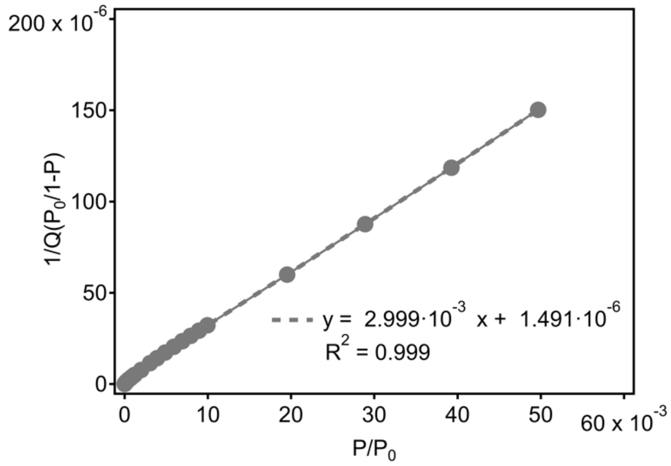

d)

**BET Area (m^2 g^{-1})**	1568.2989 ± 7.3007
**Slope (g/cm^3)**	0.003000 ± 0.000015
**Intercept (g/cm^3)**	0.000001 ± 0.000000
**R^2**	0.999734
**C**	2011.971
**V_m (cm^3 g^{-1})**	333.2046
**1/(√C+1)**	0.021807

Figure S17. Analysis of the N_2 adsorption/desorption isotherm of **MUV-12(*m*-F)$_3$** at 77 K; a) N_2 adsorption isotherm; b) Pore Size Distribution calculated by SWNT-NLDFT (regularization = 1.0), c) Multi-Point BET analysis and; d) main parameters calculated from the multi-point BET analysis

a) b)

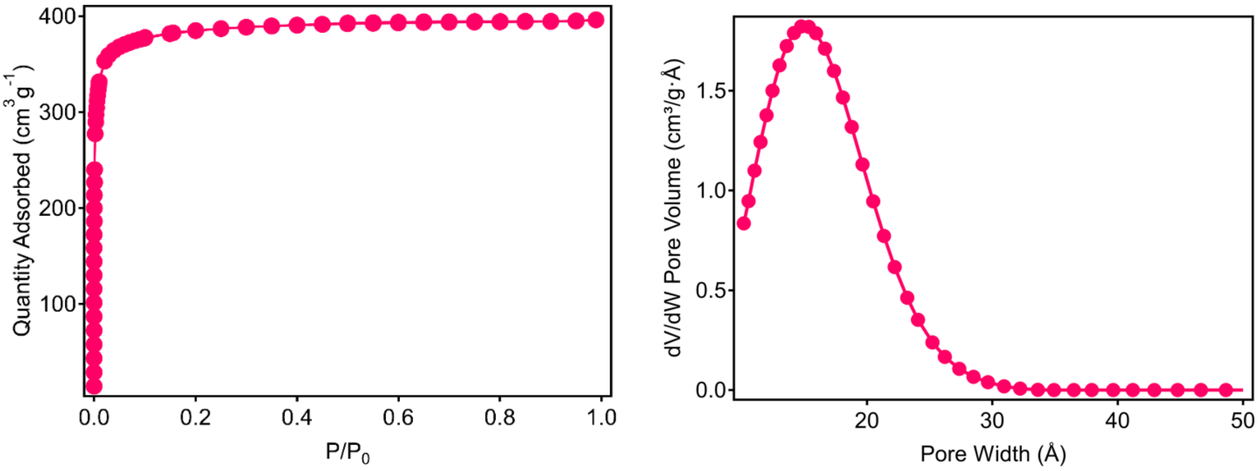

c)

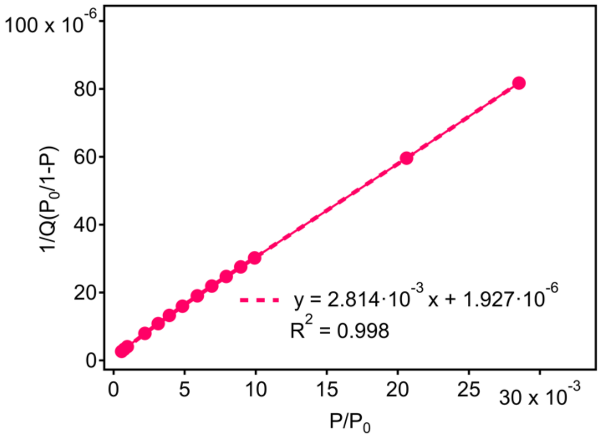

d)

**BET Area (m^2 g^{-1})**	1545.5608 ± 10.1698
**Slope (g/cm^3)**	0.002814 ± 0.000019
**Intercept (g/cm^3)**	0.000002 ± 0.000000
**R^2**	0.999740
**C**	1460.802
**V_m (cm^3 g^{-1})**	355.0909
**1/(√C+1)**	0.035496

Figure S18. Analysis of the N_2 adsorption/desorption isotherm of **MUV-12(*o*-Me)$_3$** at 77 K. a) N_2 adsorption isotherm; b) Pore Size Distribution calculated by SWNT-NLDFT (regularization = 1.0); c) Multi-Point BET analysis and; d) main parameters calculated from the multi-point BET analysis

a)

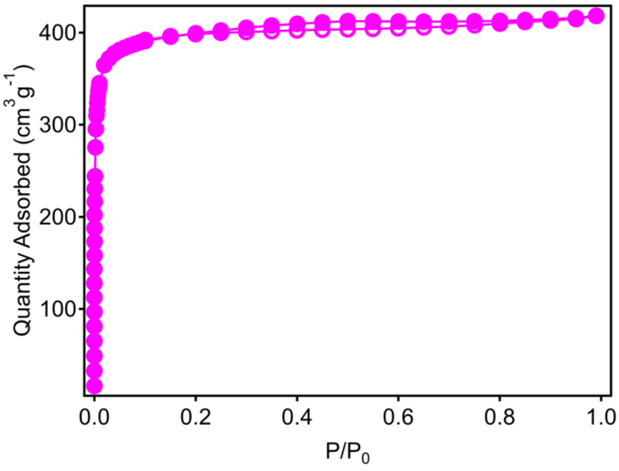

b)

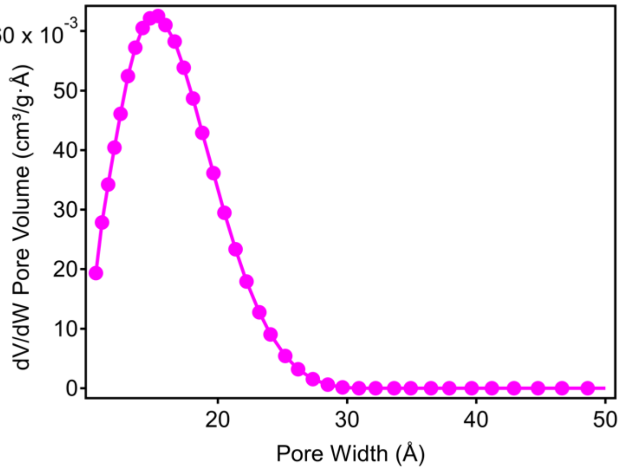

c)

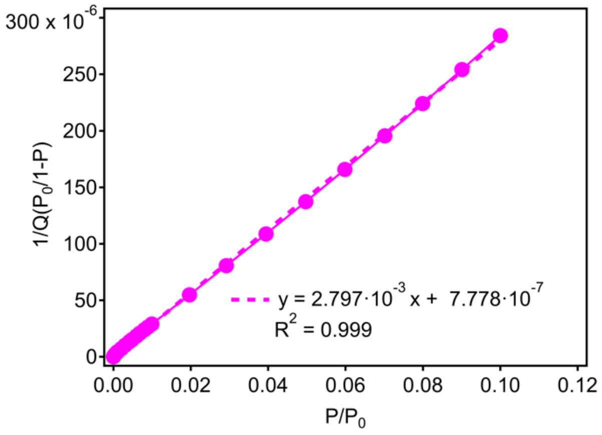

d)

**BET Area (m^2 g^{-1})**	1555.7141 ± 4.1876
**Slope (g/cm^3)**	0.002797 ± 0.000008
**Intercept (g/cm^3)**	0.000001 ± 0.000000
**R^2**	0.999842
**C**	3596.490
**V_m (cm^3 g^{-1})**	357.4236
**1/(√C+1)**	0.016401

Figure S19. Analysis of the N_2 adsorption/desorption isotherm of **MUV-12(*m*-Me)$_3$** at 77 K. a) N_2 adsorption isotherm; b) Pore Size Distribution calculated by SWNT-NLDFT (regularization = 1.0); c) Multi-Point BET analysis and d) main parameters calculated from the multi-point BET analysis

a)

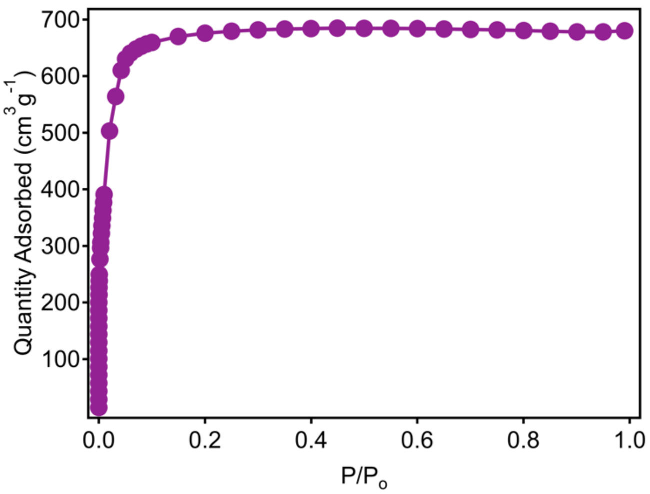

b)

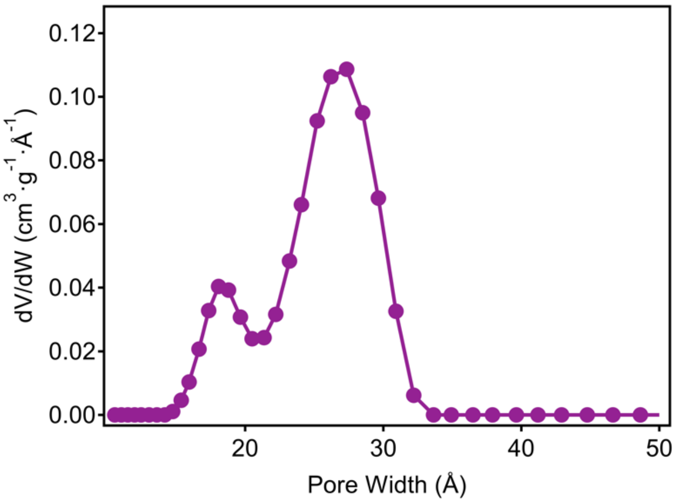

c)

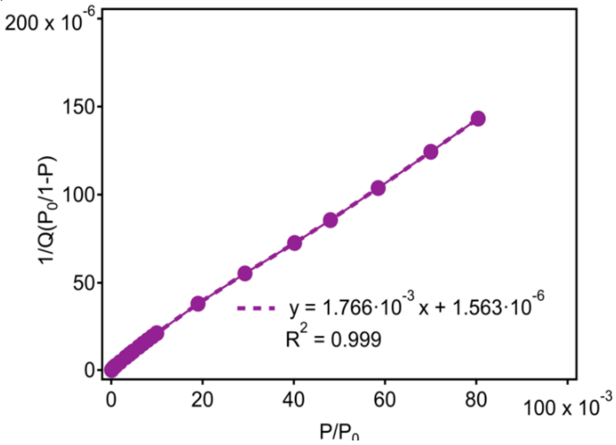

d)

**BET Area (m^2 g^{-1})**	3019.4576 ± 92.5761
**Slope (g/cm^3)**	0.001365 ± 0.000044
**Intercept (g/cm^3)**	0.000076 ± 0.000004
**R^2**	0.998446
**C**	18.87475
**V_m (cm^3 g^{-1})**	693.7170
**$1/(\sqrt{C}+1)$**	0.187197

Figure S20. Analysis of the N_2 adsorption/desorption isotherm of **MUV-12(2,6-napth)** at 77 K. a) N_2 adsorption isotherm; b) Pore Size Distribution calculated by SWNT-NLDFT (regularization = 0.1); c) Multi-Point BET analysis and d) main parameters calculated from the multi-point BET analysis

a)

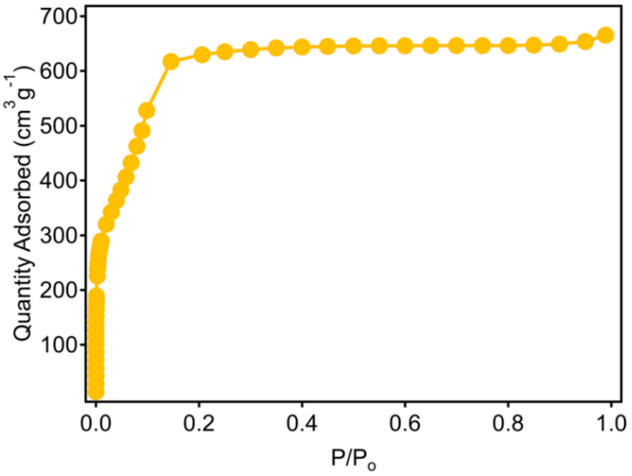

b)

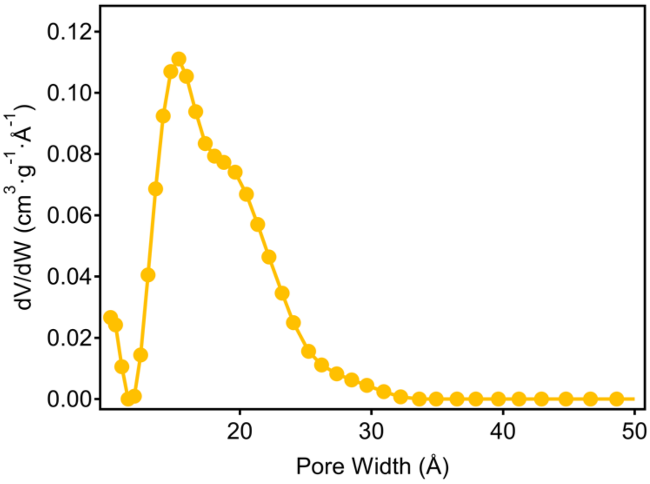

c)

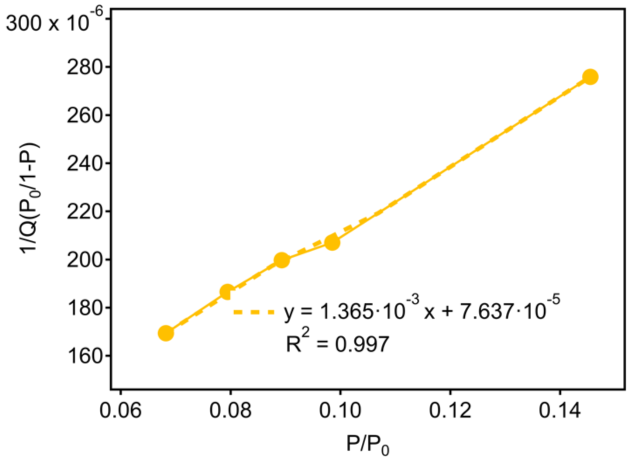

d)

**BET Area (m^2 g^{-1})**	2461.5524 ±14.3363
**Slope (g/cm^3)**	0.001767 ± 0.000010
**Intercept (g/cm^3)**	0.000002 ± 0.000000
**R^2**	0.999508
**C**	1131.176
**V_m (cm^3 g^{-1})**	565.5389
**1/(√C+1)**	0.028874

Figure S21. Analysis of the N_2 adsorption/desorption isotherm of **MUV-12(1,4-napth)** at 77 K. a) N_2 adsorption isotherm; b) Pore Size Distribution calculated by SWNT-NLDFT (regularization = 0.1); c) Multi-Point BET analysis and d) main parameters calculated from the multi-point BET analysis

a)

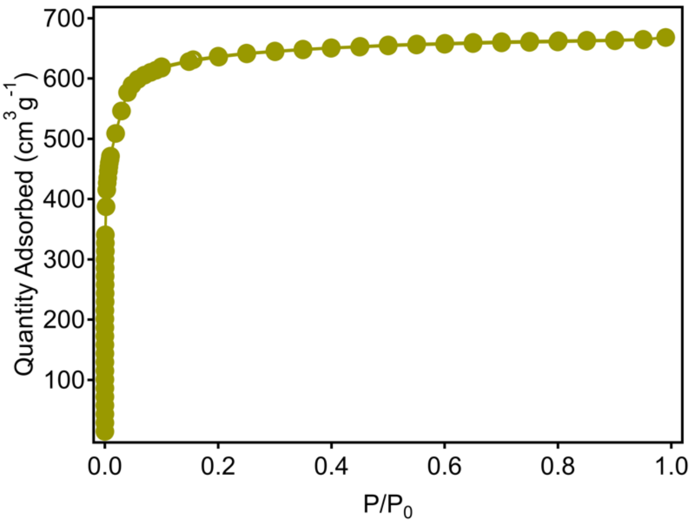

b)

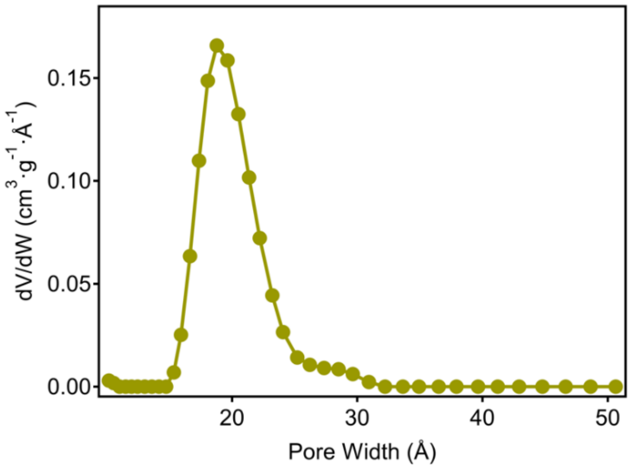

c)

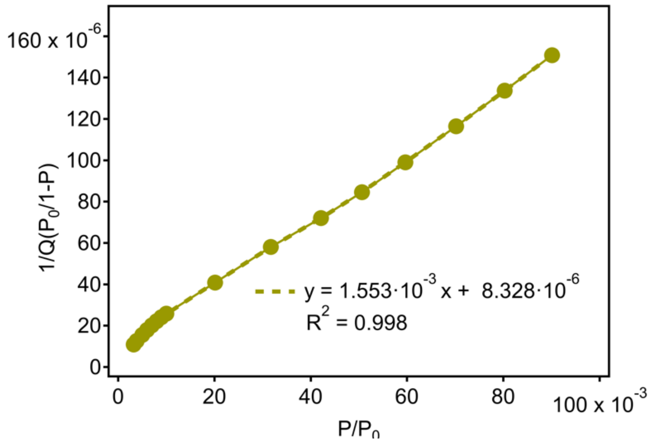

d)

**BET Area (m^2 g^{-1})**	2787.8142 ± 26.0532
**Slope (g/cm^3)**	0.001553 ± 0.000015
**Intercept (g/cm^3)**	0.000008 ± 0.000001
**R^2**	0.999383
**C**	187.4790
**V_m (cm^3 g^{-1})**	640.4972
**$1/(\sqrt{C}+1)$**	0.068063

Figure S22. Analysis of the N_2 adsorption/desorption isotherm of **MUV-12(anth)** at 77 K. a) N_2 adsorption isotherm, b) Pore Size Distribution calculated by SWNT-NLDFT (regularization = 0.1), c) Multi-Point BET analysis and d) main parameters calculated from the multi-point BET analysis

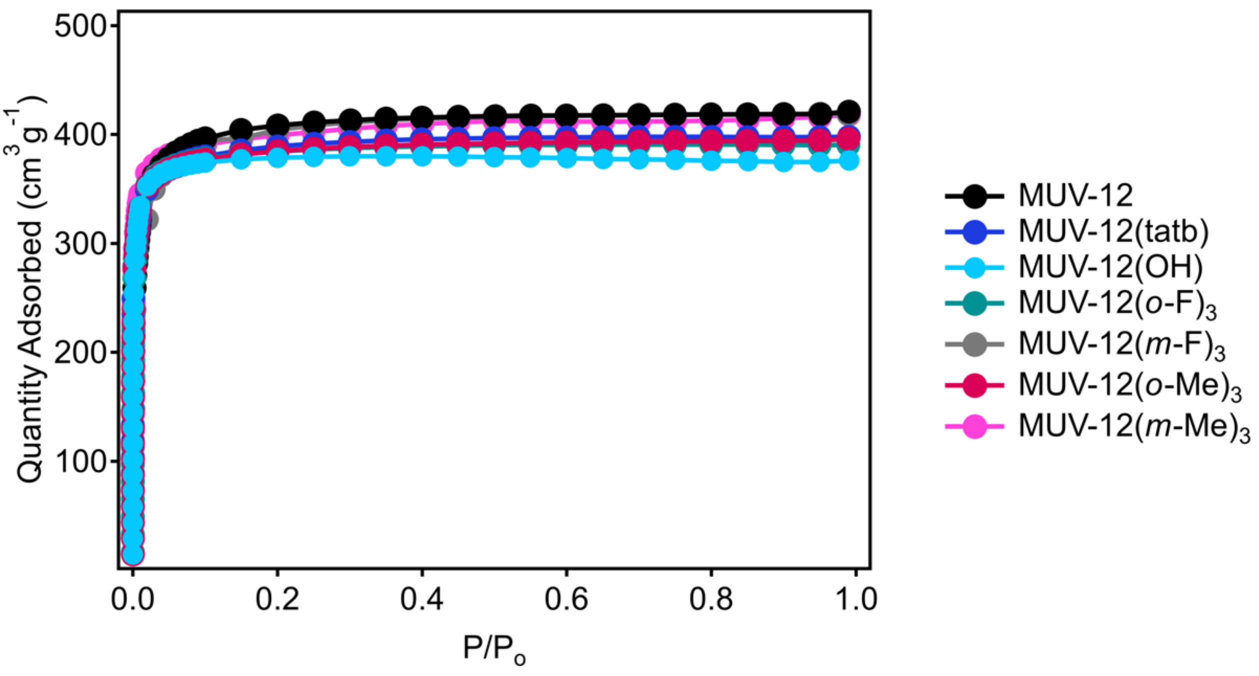

Figure S23. Comparison of N_2 isotherm at 77 K of the **MUV-12(X)** materials

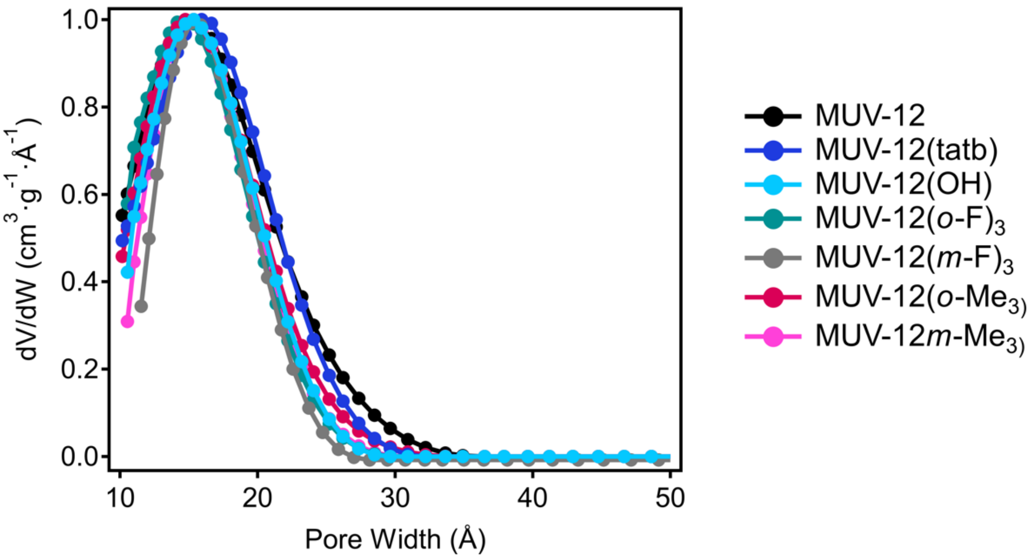

Figure S24. Comparison of Pore Size Distribution of the **MUV-12(X)** materials

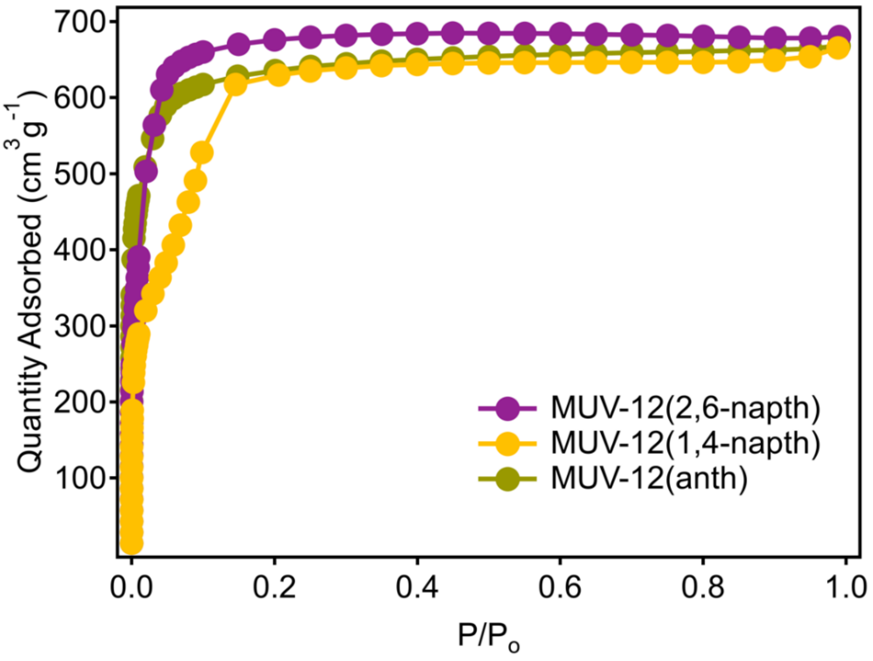

Figure S25. Comparison of N_2 isotherm at 77 K of the **MUV-12(Y)** materials

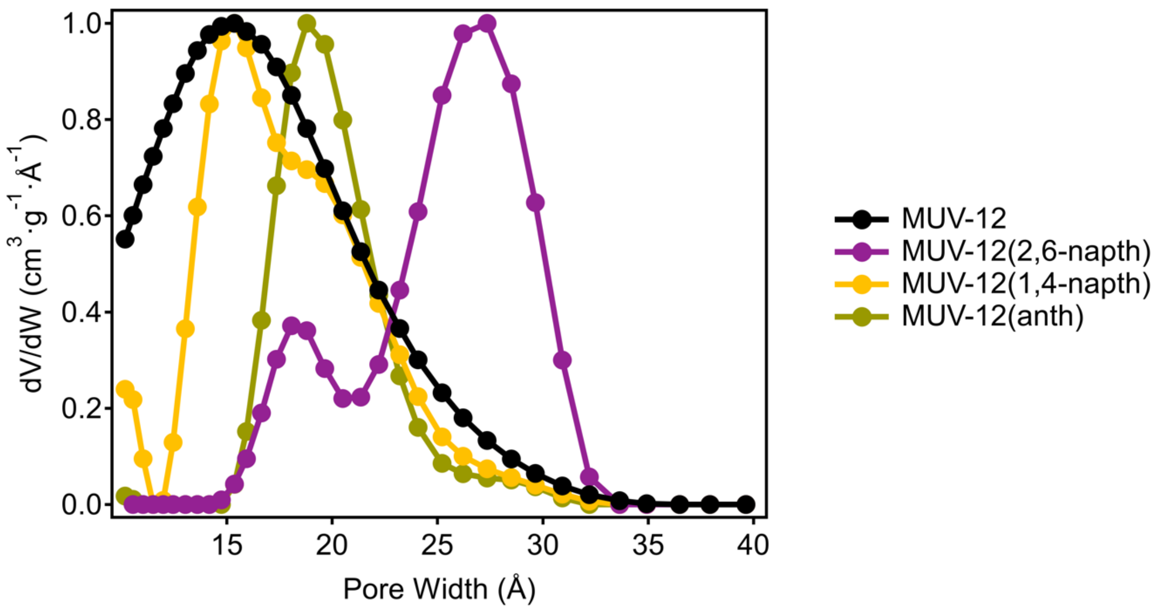

Figure S26. Comparison of Pore Size Distribution of the **MUV-12(Y)** materials and MUV-12 as reference

Table S6. Summary of the experimental adsorption data of **MUV-12(X)** materials

Framework	SA_{BET}[a] $[m^2 \cdot g^{-1}]$	V_t[b] $[cm^3 \cdot g^{-1}]$	PSD_{NLDFT}[C] [nm]	
			micro	meso
MUV-12	1589.909	0.570	15.2	-
MUV-12(tatb)	1514.946	0.543	15.7	-
MUV-12(OH)	1493.624	0.535	15.2	-
MUV-12(*o*-F)$_3$	1533.592	0.531	14.8	-
MUV-12(*m*-F)$_3$	1568.299	0.562	14.2	-
MUV-12(*o*-Me)$_3$	1545.561	0.536	14.1	-
MUV-12(*m*-Me)$_3$	1555.714	0.611	15.1	-

[a] Specific surface area (Sa) was calculated by multi-point Brunauer-Emmett-Teller (BET) method at P/P_0 = 0.01-0.08. [b] Total pore volume at P/P_0=0.96. [c] Pore size distribution was analyzed by using the solid density functional theory (SWNT-NLDFT Carb Cyl Mesopore; regularization = 1.000) for the adsorption branch assuming a cylindrical pore model.

Table S7. Summary of the experimental adsorption data of **MUV-12(Y)** materials

Framework	SA_{BET}[a] $[m^2 \cdot g^{-1}]$	V_t[b] $[cm^3 \cdot g^{-1}]$	PSD_{NLDFT}[C] [nm]	
			micro	meso
MUV-12(2,6-napth)	3019.458	0.904	18.1	27.3
MUV-12(1,4-napth)	2461.552	0.908	15.3	19.5
MUV-12(anth)	2787.814	0.938	-	18.7

[a] Specific surface area (Sa) was calculated by multi-point Brunauer-Emmett-Teller (BET) method at P/P_0 = 0.01-0.08. [b] Total pore volume at P/P_0=0.96.[c] Pore size distribution was analyzed by using the solid density functional theory (SWNT-NLDFT Carb Cyl Mesopore; regularization = 0.1000) for the adsorption branch assuming a cylindrical pore model.

S.6.2. Pore Volume and Surface Area calculations

The geometrical surface area values and probe occupied volumes were obtained by analyzing the crystallographic structures of MUV-12 and functionalized MUV-12(X) and MUV-12(Y) frameworks by using MoloVol 1.1.0[13] with a small probe radius of 1.66 Å (Nitrogen). Elements radii: Ti (2.46 Å), F (1.46 Å), O (1.5 Å), H (1.2 Å), Ca (2.62 Å) and C (1.77 Å).

Table S8. Summary of the computational analysis.

Framework	$SA_{BET}{}^{a}$ [$m^2 \cdot g^{-1}$]	$V_t{}^{b}$ [$cm^3 \cdot g^{-1}$]
MUV-12	2229.05	0.776
MUV-12(tatb)	1960.69	0.753
MUV-12(OH)	1647.34	0.631
MUV-12(*o*-F)$_3$	1592.84	0.612
MUV-12(*m*-F)$_3$	1829.97	0.630
MUV-12(*o*-Me)$_3$	1486.11	0.559
MUV-12(*m*-Me)$_3$	1756.26	0.669
MUV-12(2,6-napth)	3987.08	1.442
MUV-12(1,4-napth)	3435.27	1.154
MUV-12(anth)	2626.89	1.005

[a] Probe accessible surface area similar to Lee-Richards surface.

[b] Probe occupied volume.

S.6.3. Scanning Electron Microscopy (SEM-EDX)

Particle morphologies and dimensions were studied with a Hitachi S-4800 scanning electron microscope at an accelerating voltage of 20 kV, on samples metalized with a mixture of gold and palladium during 90 seconds. SEM-Energy-dispersive X-Ray analysis (EDX) of the materials were also studied. Mapping of Ca (blue), Ti (green) and F (orange) confirms that element distribution is homogeneous with small statistic deviation across the solid. Experimental metal radio % from point and shoot EDX analysis for different crystals in the solids is represented in the histogram below.

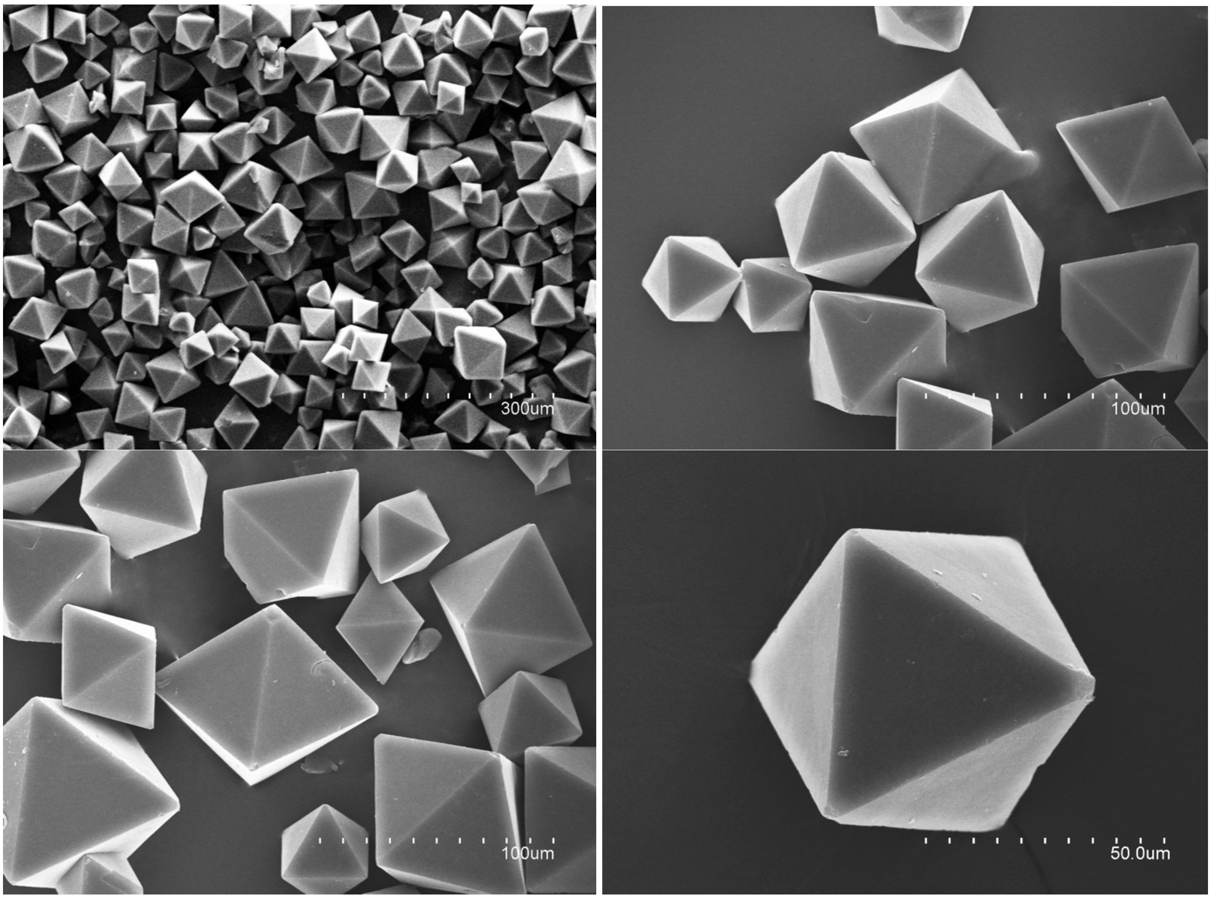

Figure S27. Scanning Electron Microscopy (SEM) images of **MUV-12**

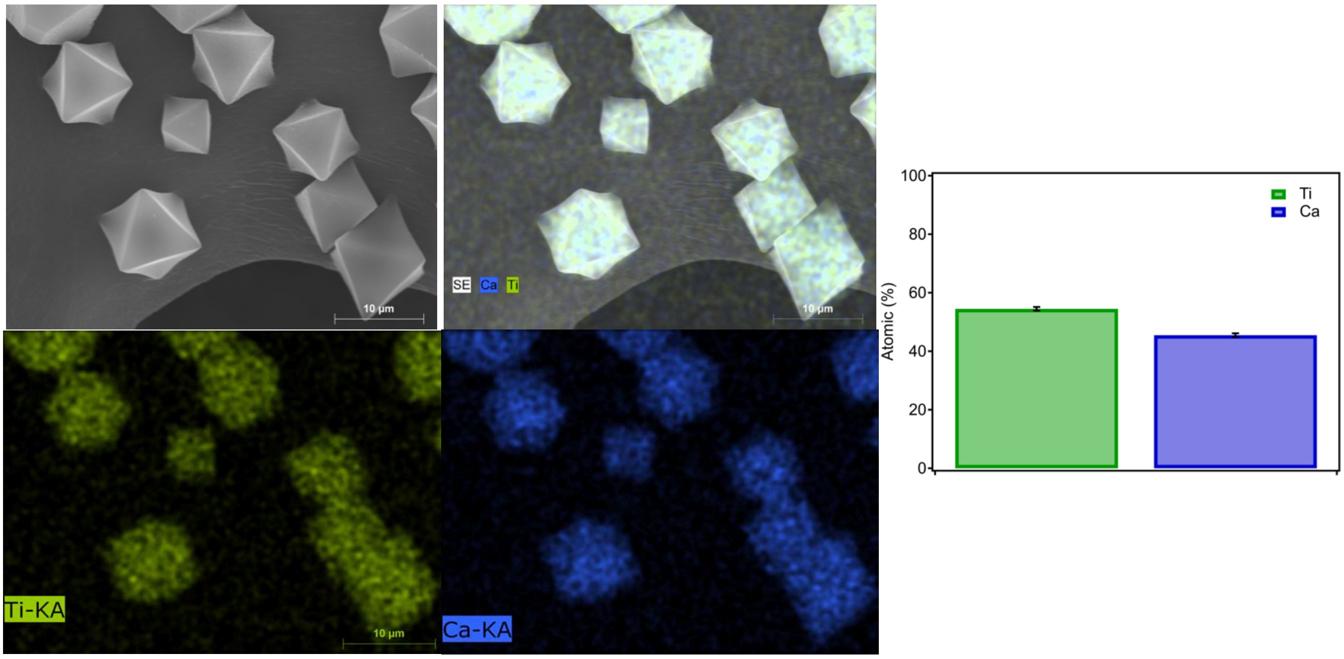

Figure S28. Mapping of **MUV-12** showing Ti (green), Ca (blue) and Experimental Ti:Ca ratio % from point and shoot EDX analysis showing the standard deviation (s) of n samples (n=3)

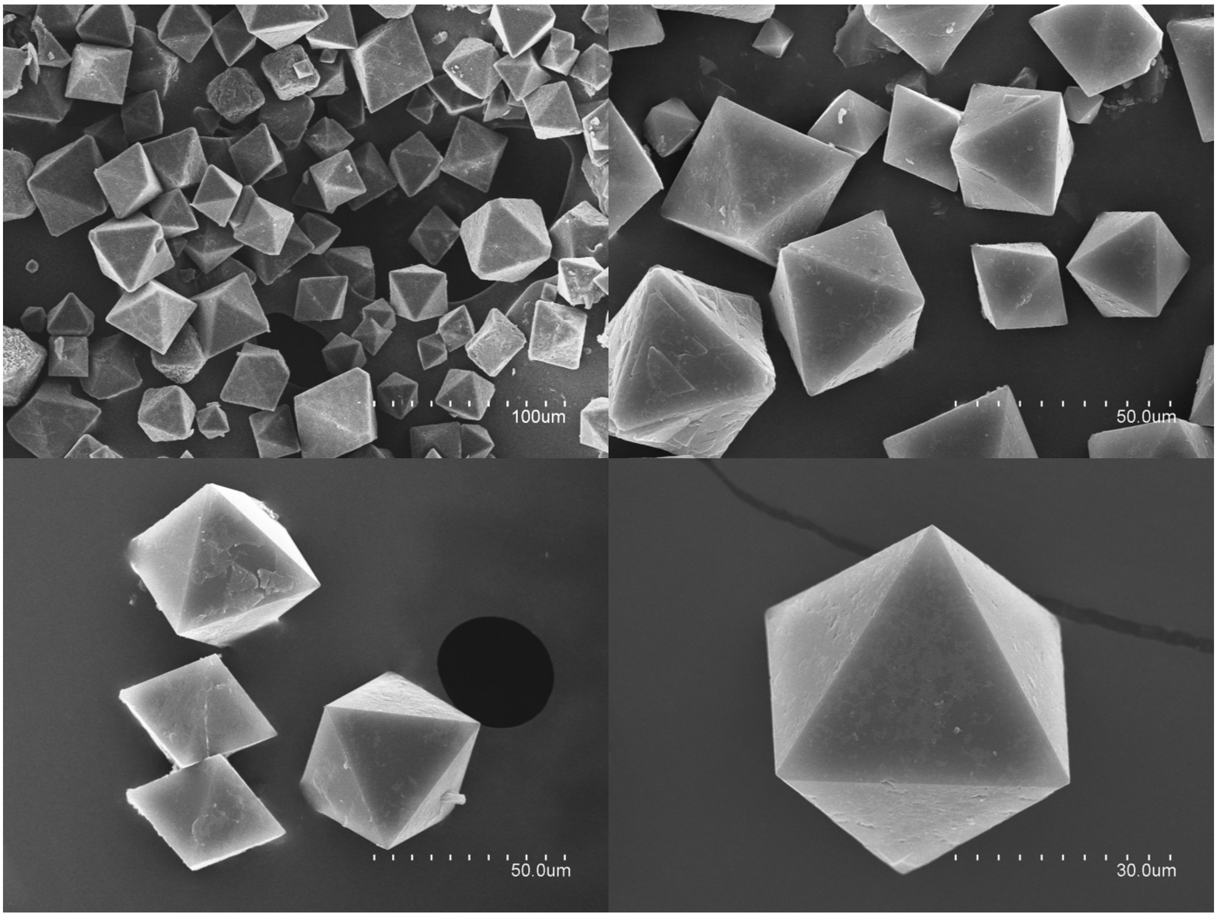

Figure S29. Scanning Electron Microscopy (SEM) images of **MUV-12(tatb)**

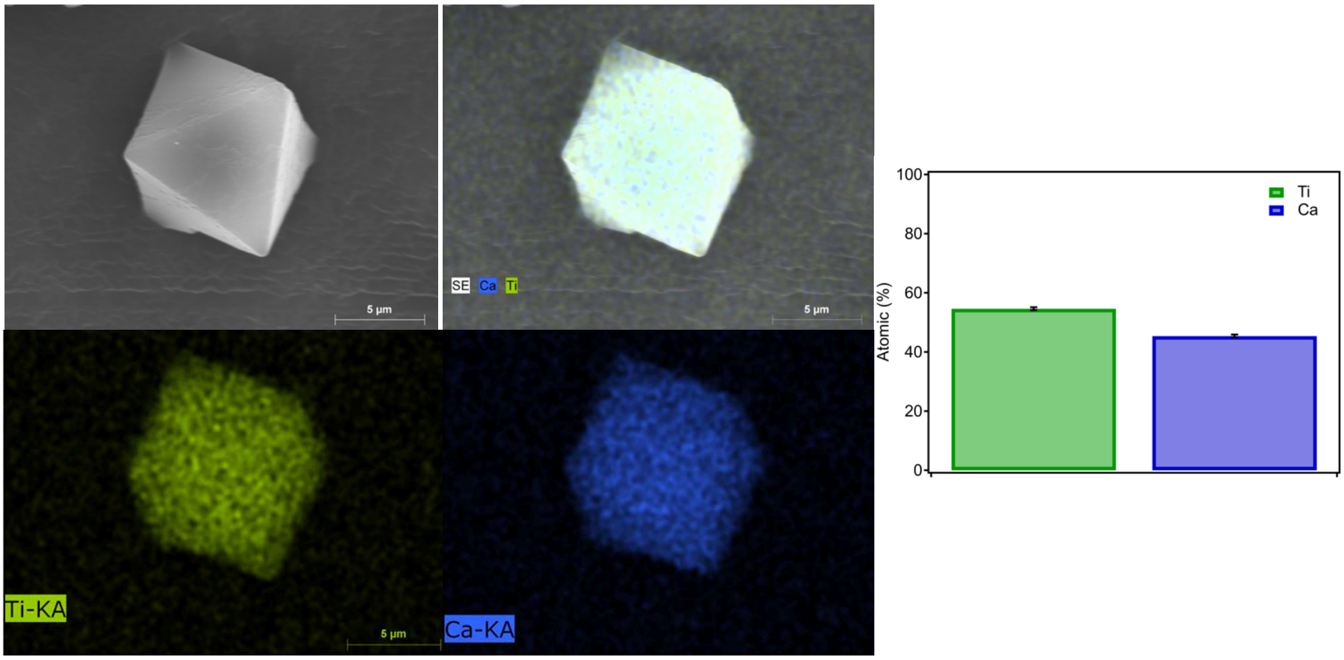

Figure S30. Mapping of **MUV-12(tatb)** showing Ti (green), Ca (blue) and Experimental Ti:Ca ratio % from point and shoot EDX analysis showing the standard deviation (s) of n samples (n=3)

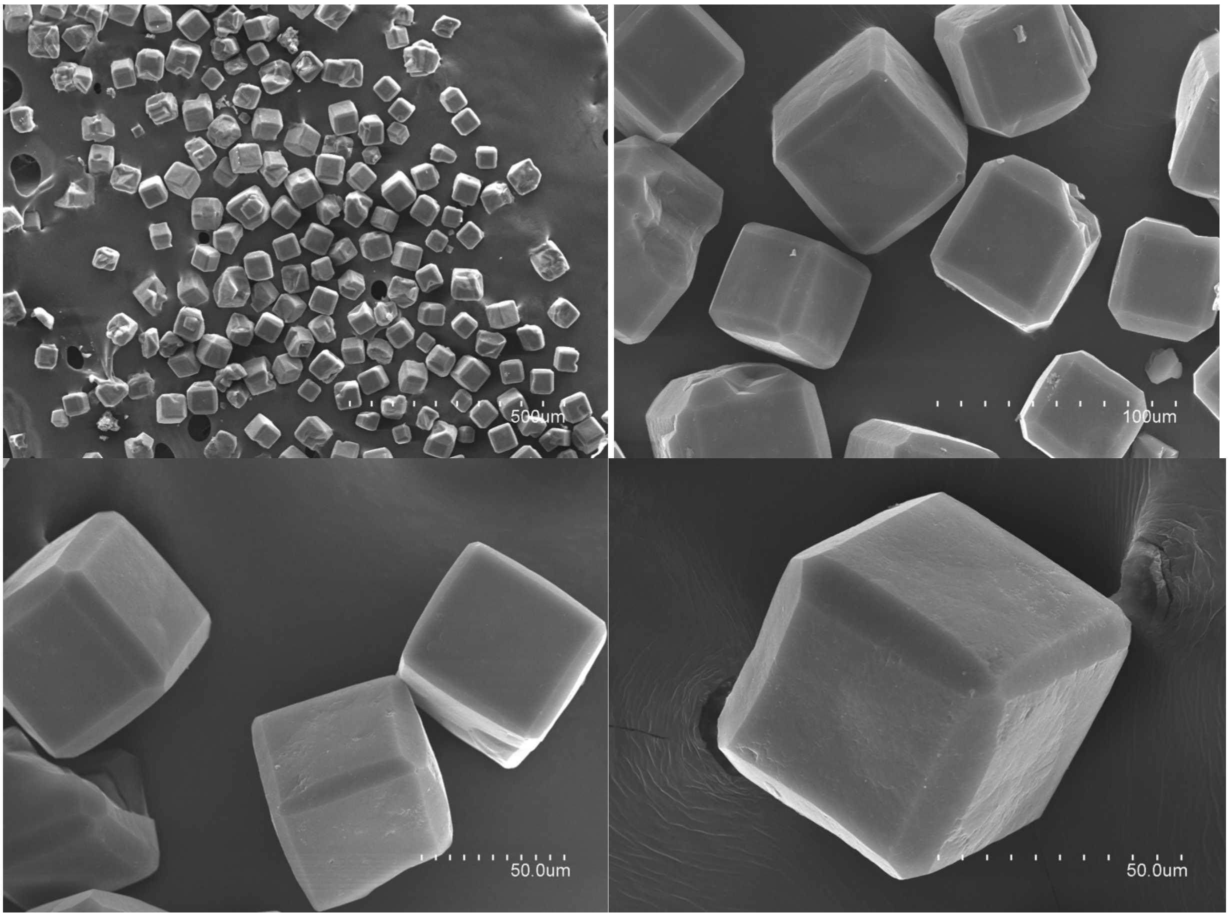

Figure S31. Scanning Electron Microscopy (SEM) images of **MUV-12(OH)**

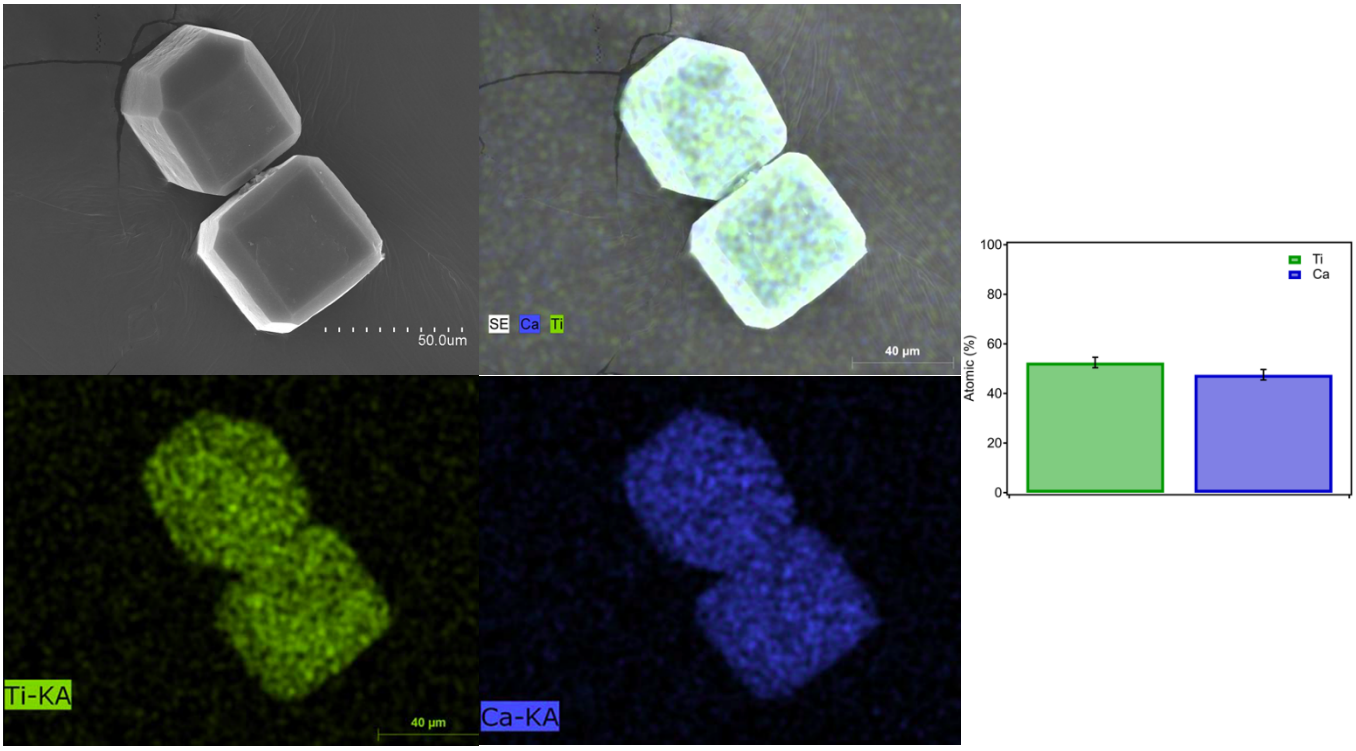

Figure S32. Mapping of **MUV-12(OH)** showing Ti (green), Ca (blue) and Experimental Ti:Ca ratio % from point and shoot EDX analysis showing the standard deviation (s) of n samples (n=3)

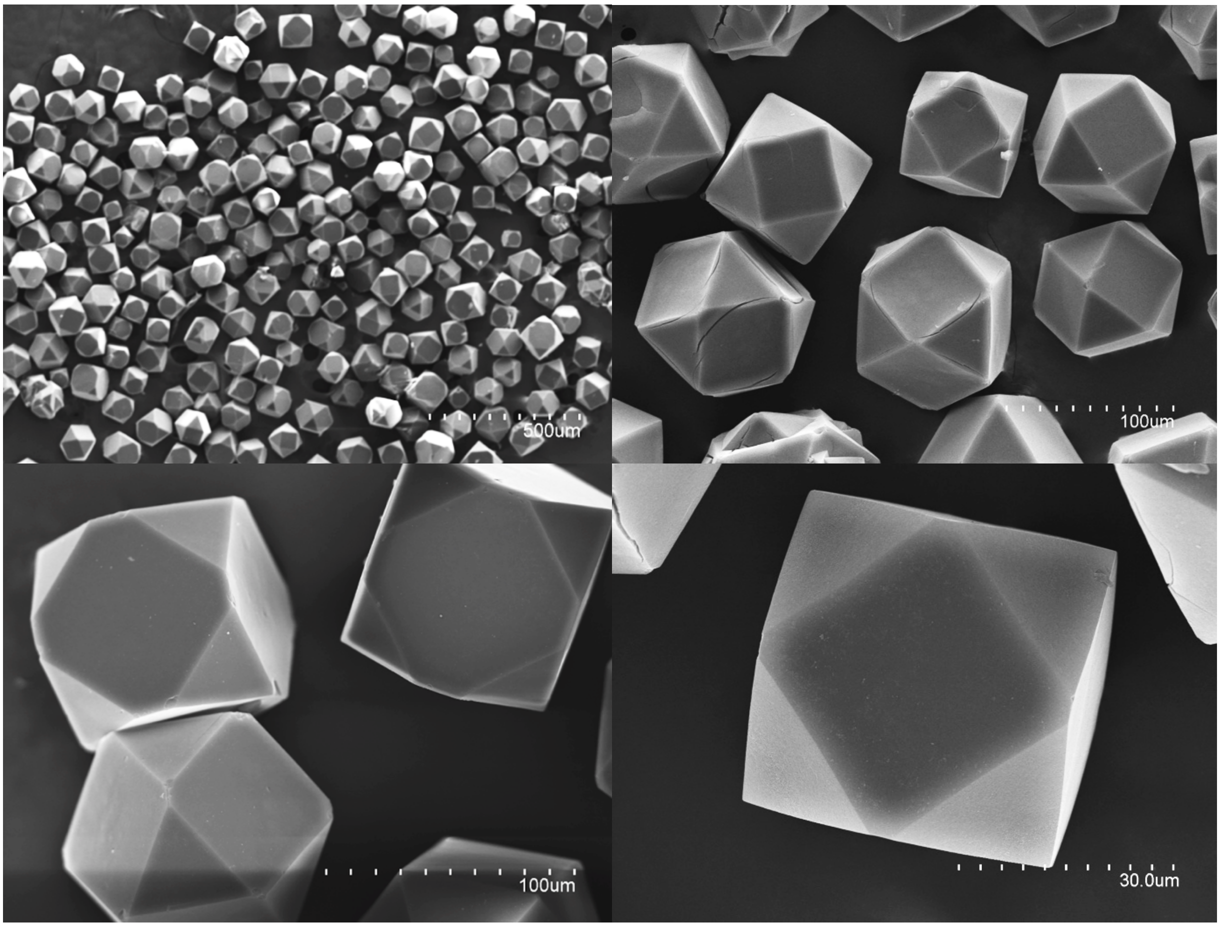

Figure S33. Scanning Electron Microscopy (SEM) images of **MUV-12(*o*-F)$_3$**

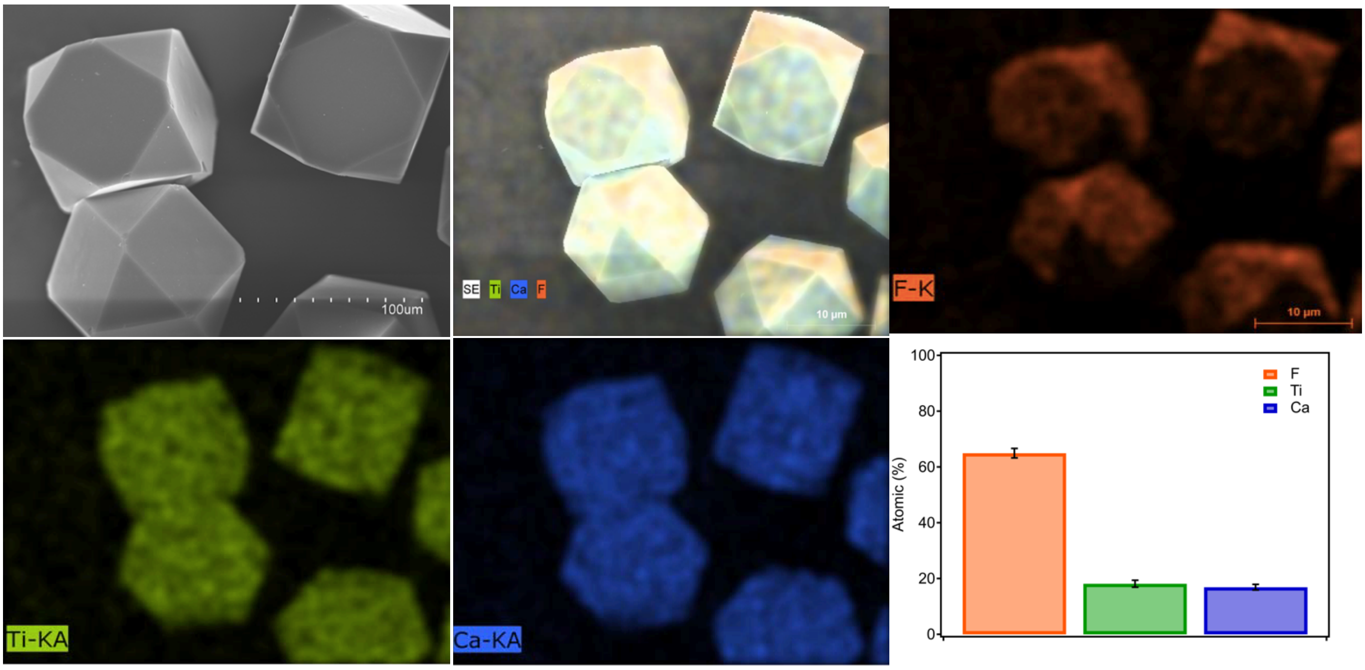

Figure S34. Mapping of **MUV-12(*o*-F)$_3$** showing Ti (green), Ca (blue), F (orange) and Experimental Ti:Ca:F ratio % from point and shoot EDX analysis showing the standard deviation (s) of n samples (n=3)

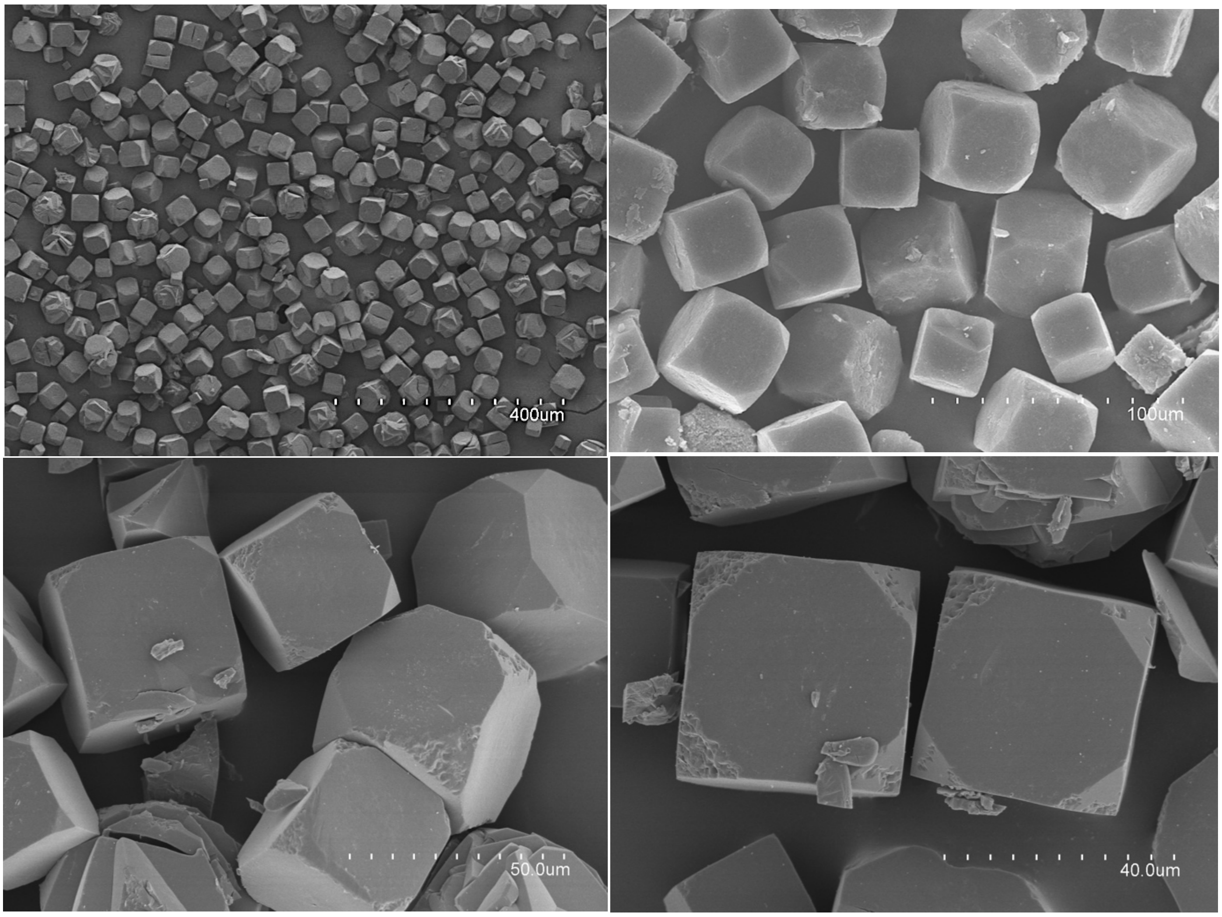

Figure S35. Scanning Electron Microscopy (SEM) images of **MUV-12(*m*-F)$_3$**

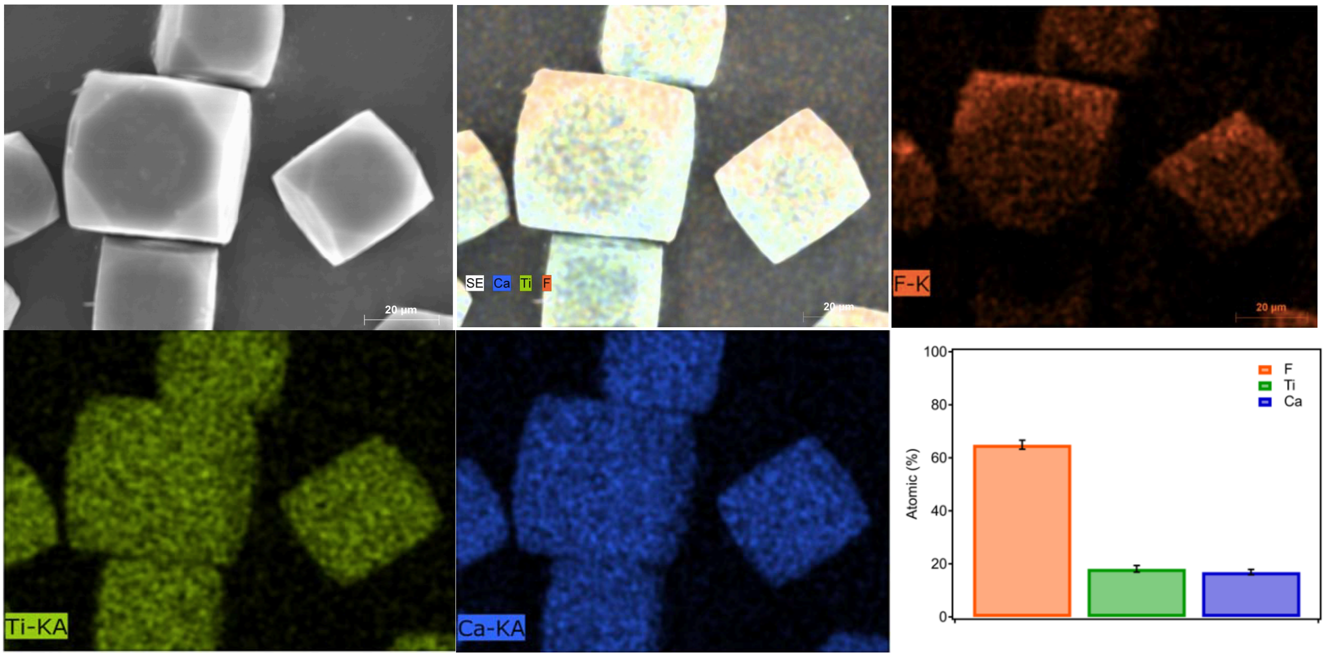

Figure S36. Mapping of **MUV-12(*m*-F)$_3$** showing Ti (green), Ca (blue), F (orange) and Experimental Ti:Ca:F ratio % from point and shoot EDX analysis showing the standard deviation (s) of n samples (n=3)

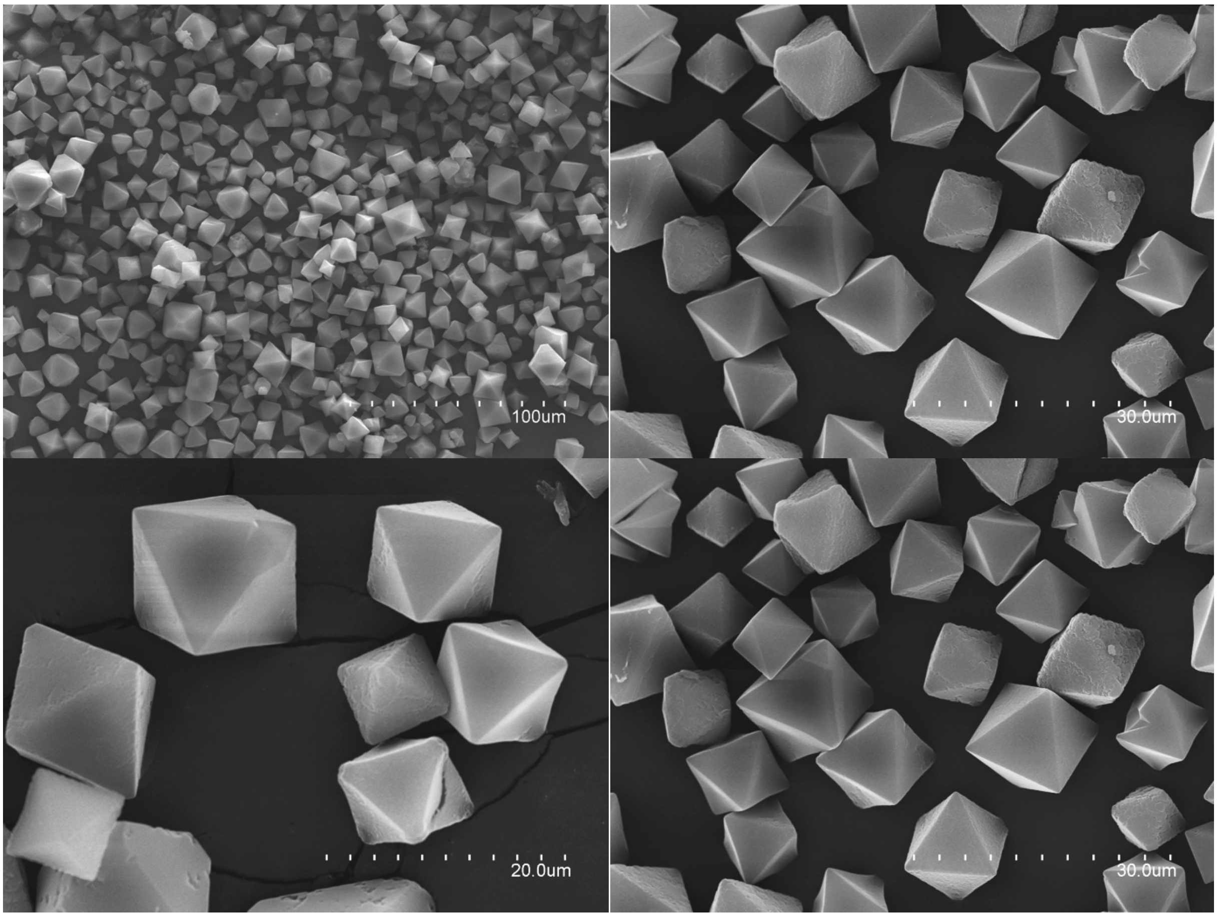

Figure S37. Scanning Electron Microscopy (SEM) images of **MUV-12(*o*-Me)$_3$**

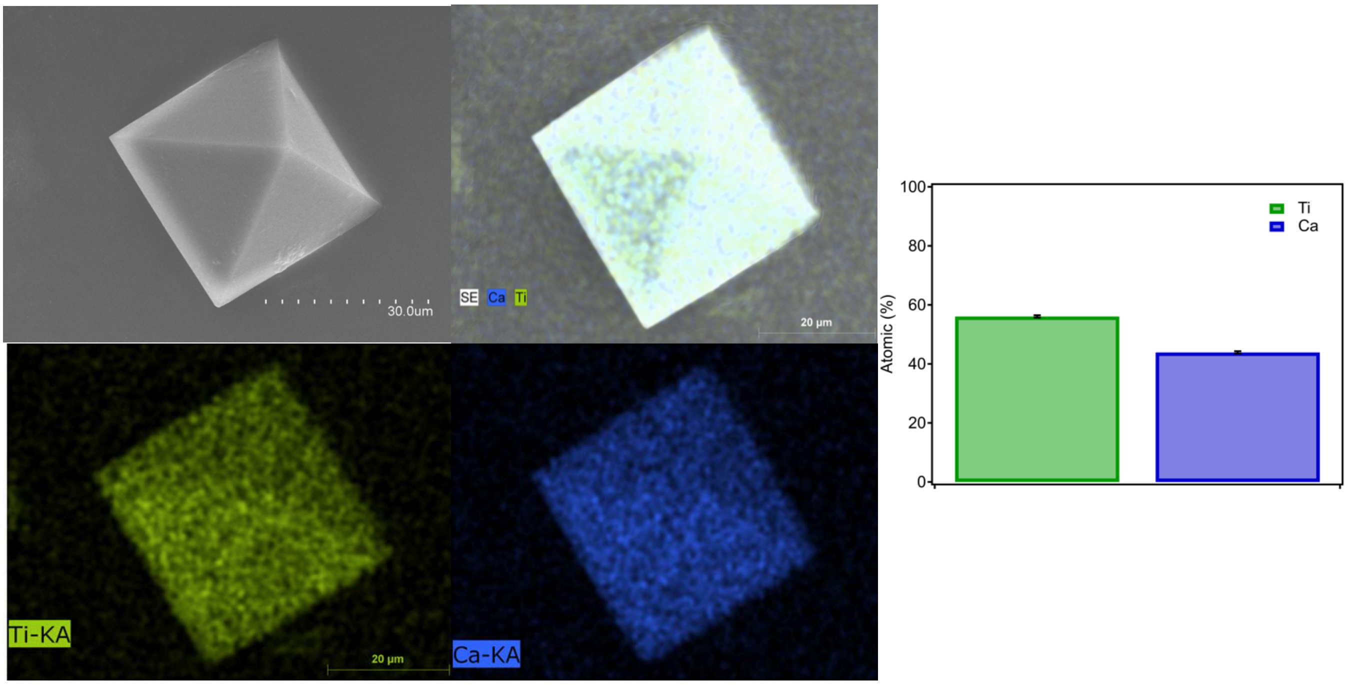

Figure S38. Mapping of **MUV-12(*o*-Me)$_3$** showing Ti (green), Ca (blue) and Experimental Ti:Ca ratio % from point and shoot EDX analysis showing the standard deviation (s) of n samples (n=3)

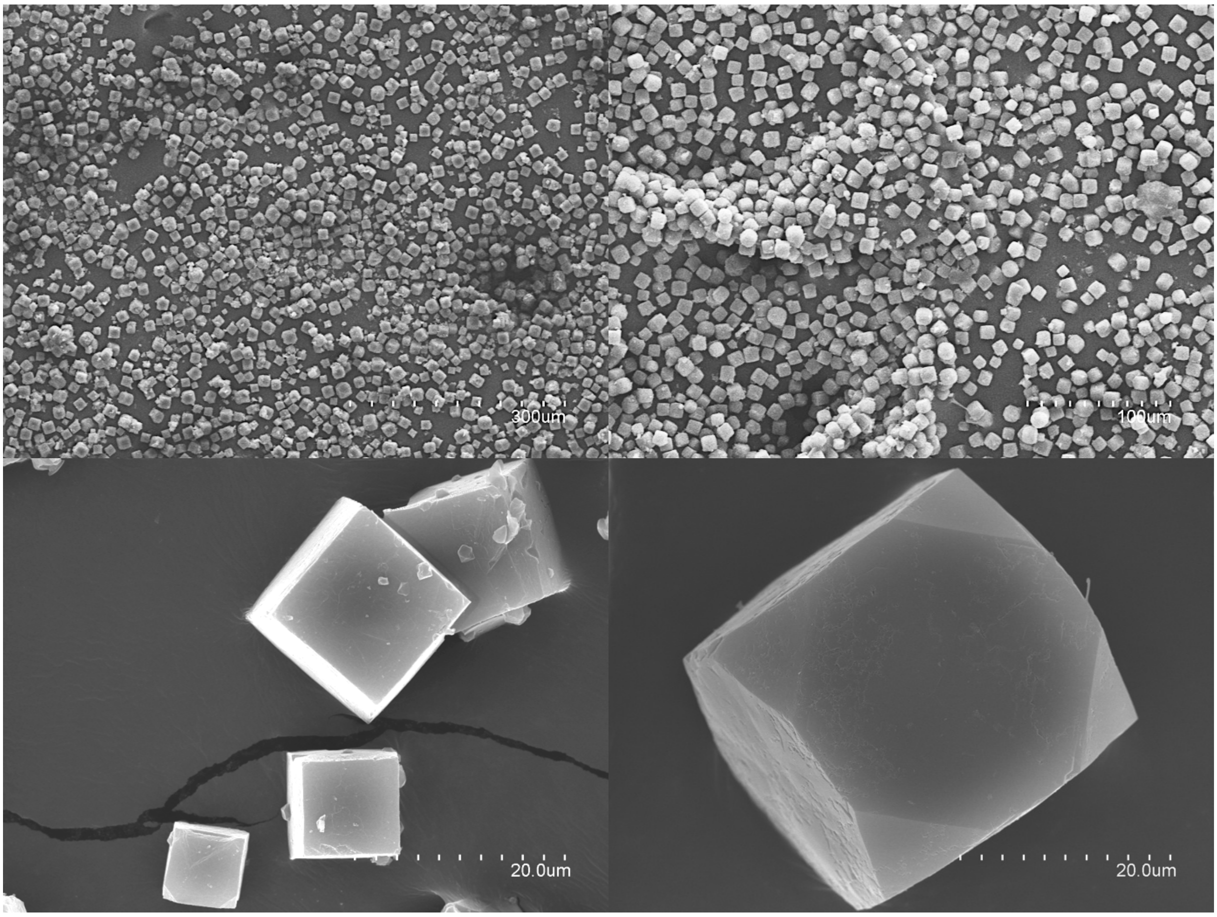

Figure S39. Scanning Electron Microscopy (SEM) images of **MUV-12(*m*-Me)$_3$**

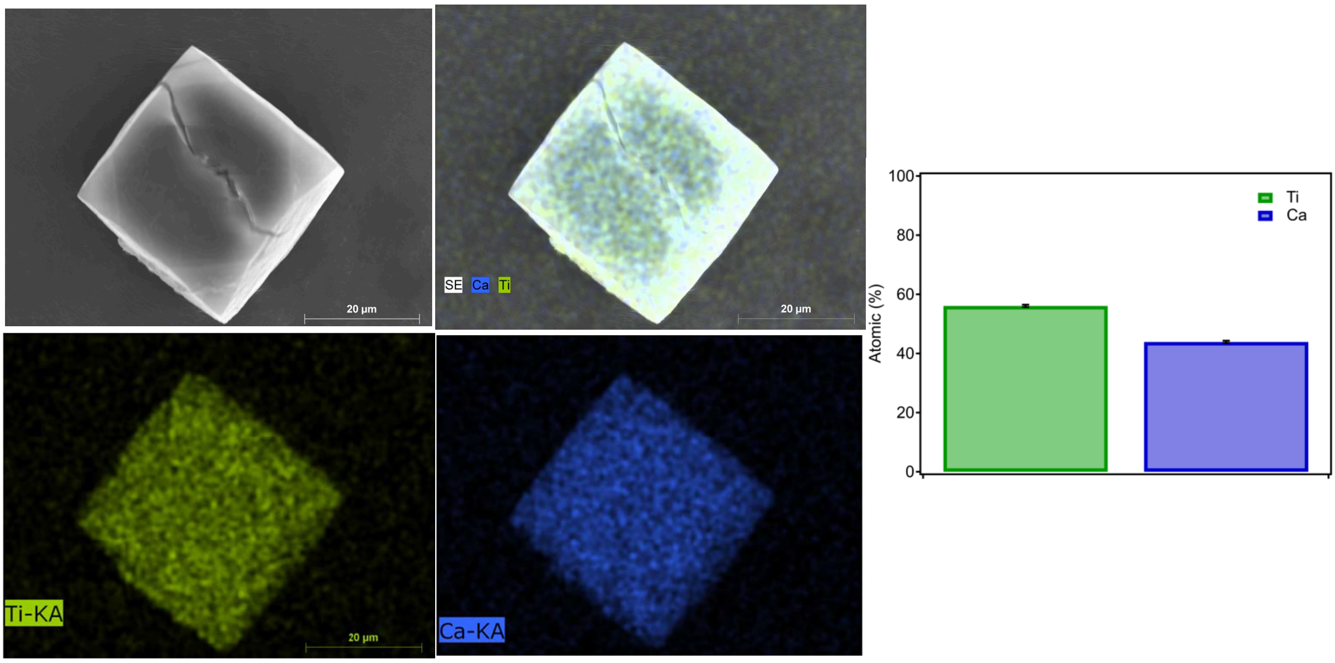

Figure S40. Mapping of **MUV-12(*m*-Me)$_3$** showing Ti (green), Ca (blue) and Experimental Ti:Ca ratio % from point and shoot EDX analysis showing the standard deviation (s) of n samples (n=3)

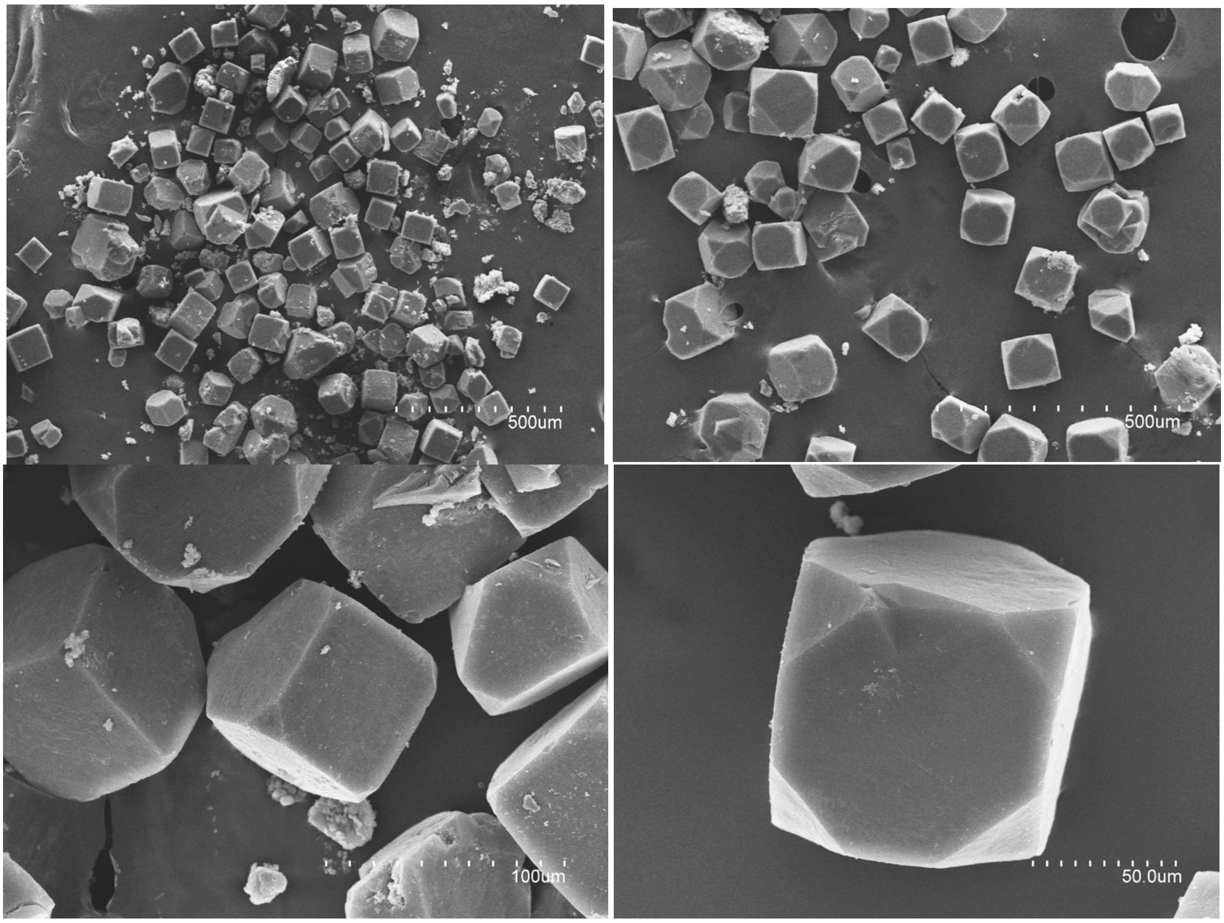

Figure S41. Scanning Electron Microscopy (SEM) images of **MUV-12(1,4-napth)**

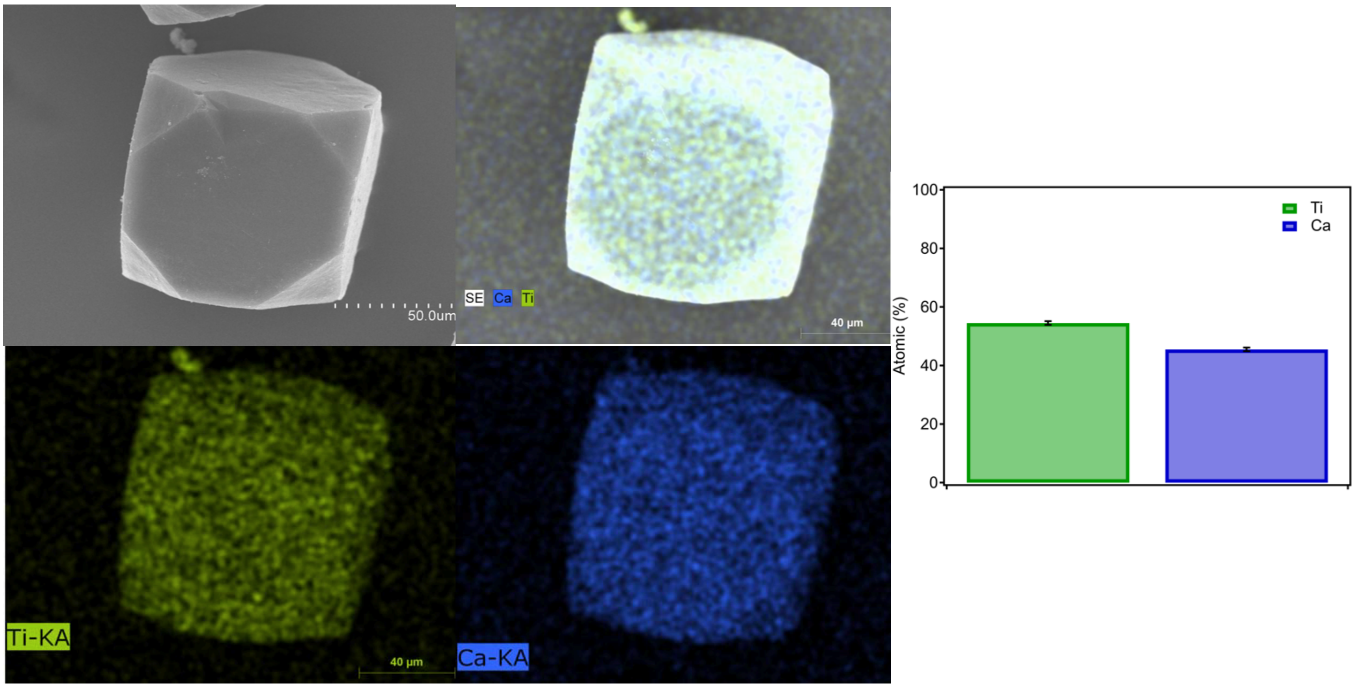

Figure S42. Mapping of **MUV-12(1,4-napth)** showing Ti (green), Ca (blue) and Experimental Ti:Ca ratio % from point and shoot EDX analysis showing the standard deviation (s) of n samples (n=3)

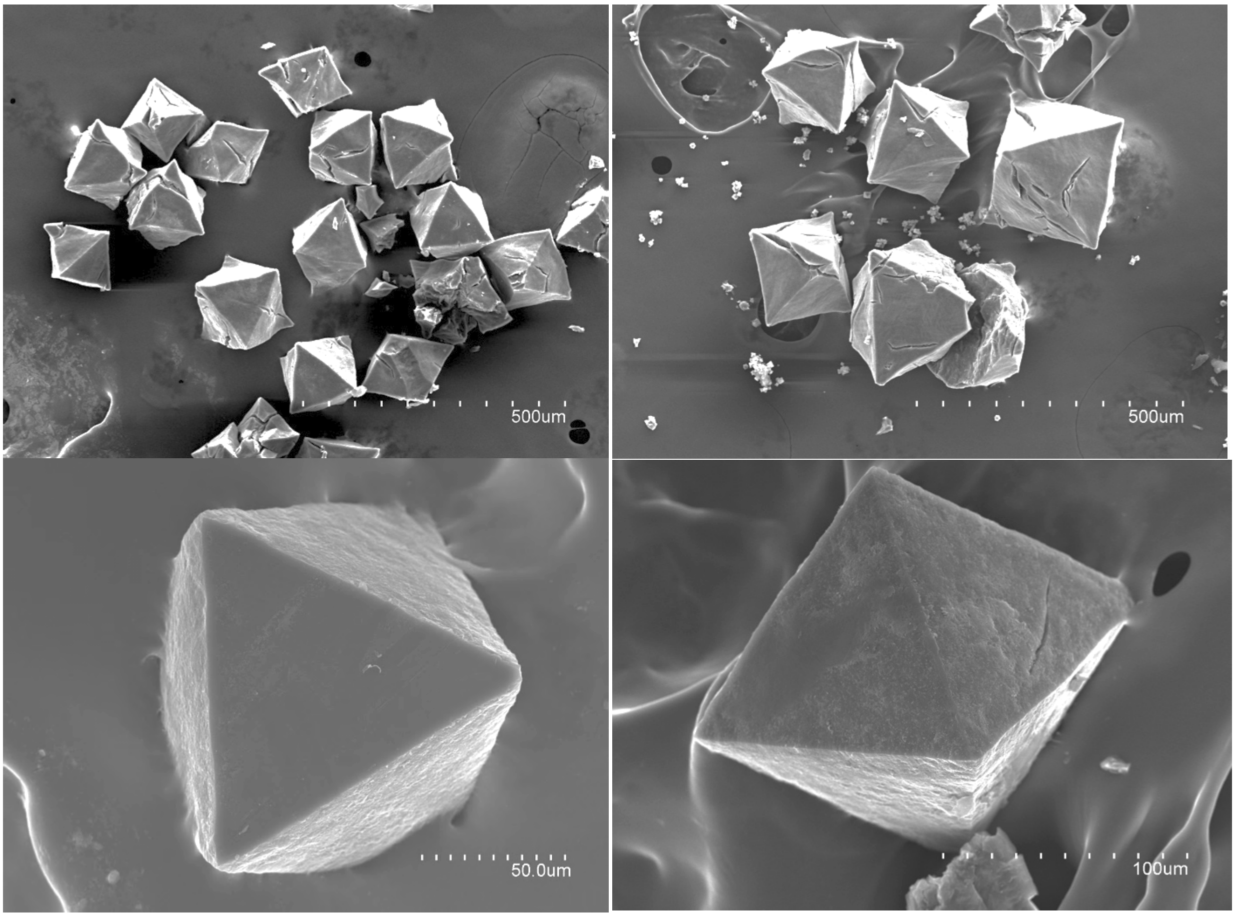

Figure S43. Scanning Electron Microscopy (SEM) images of **MUV-12(2,6-napth)**

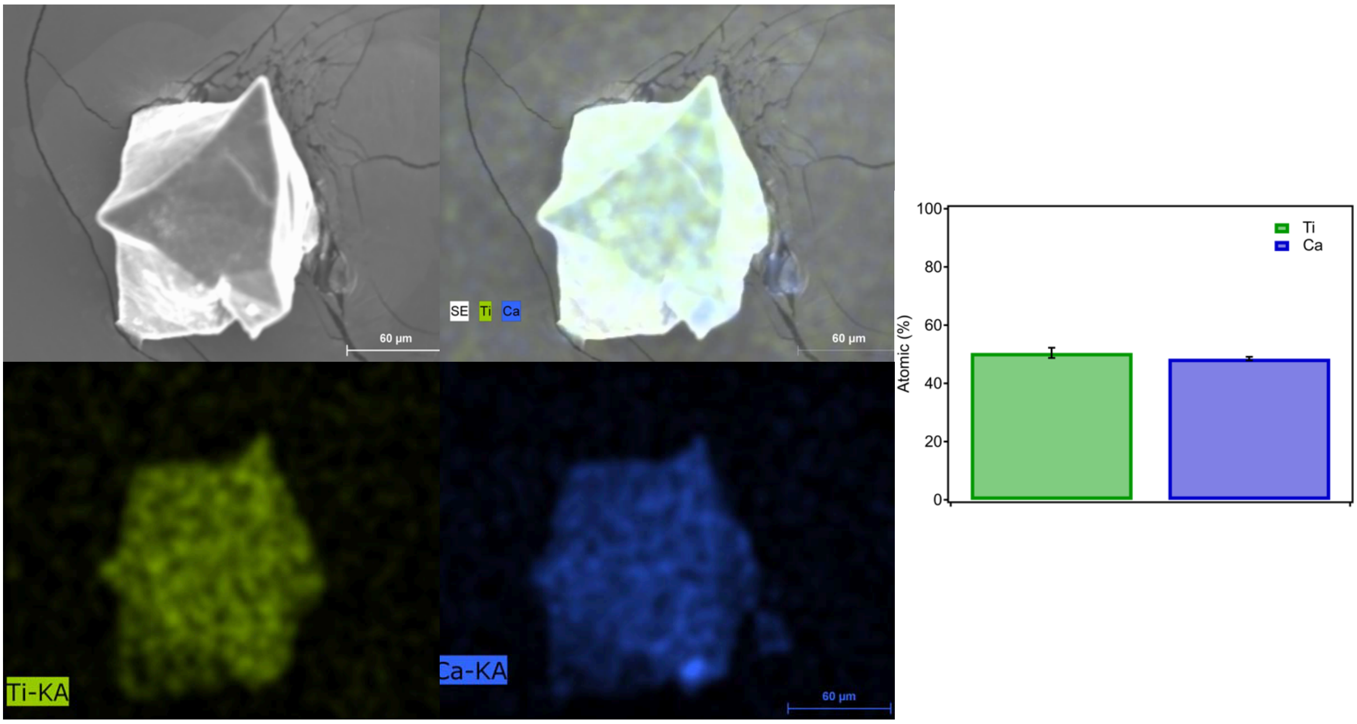

Figure S44. Mapping of **MUV-12(2,6-napth)** showing Ti (green), Ca (blue) and Experimental Ti:Ca ratio % from point and shoot EDX analysis showing the standard deviation (s) of n samples (n=3)

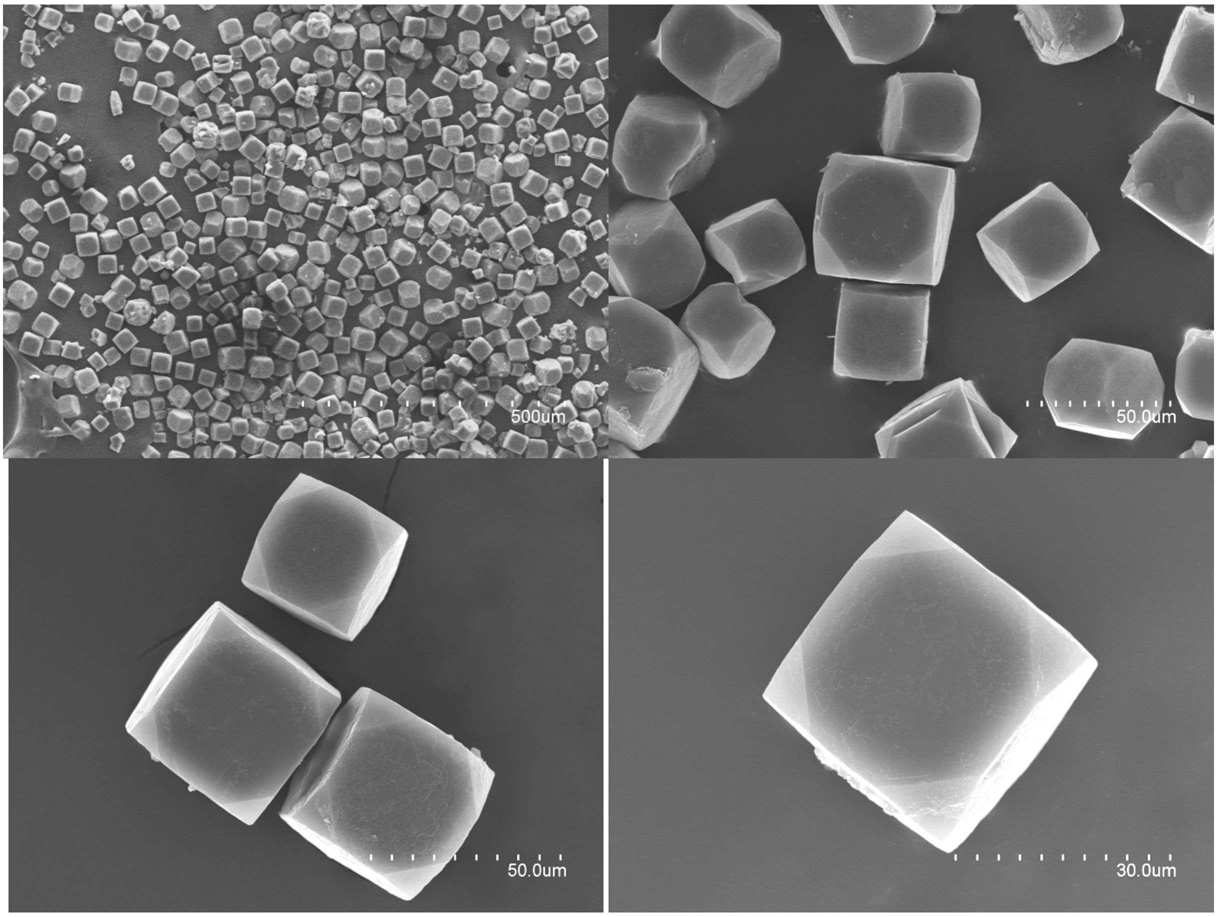

Figure S45. Scanning Electron Microscopy (SEM) images of **MUV-12(anth)**

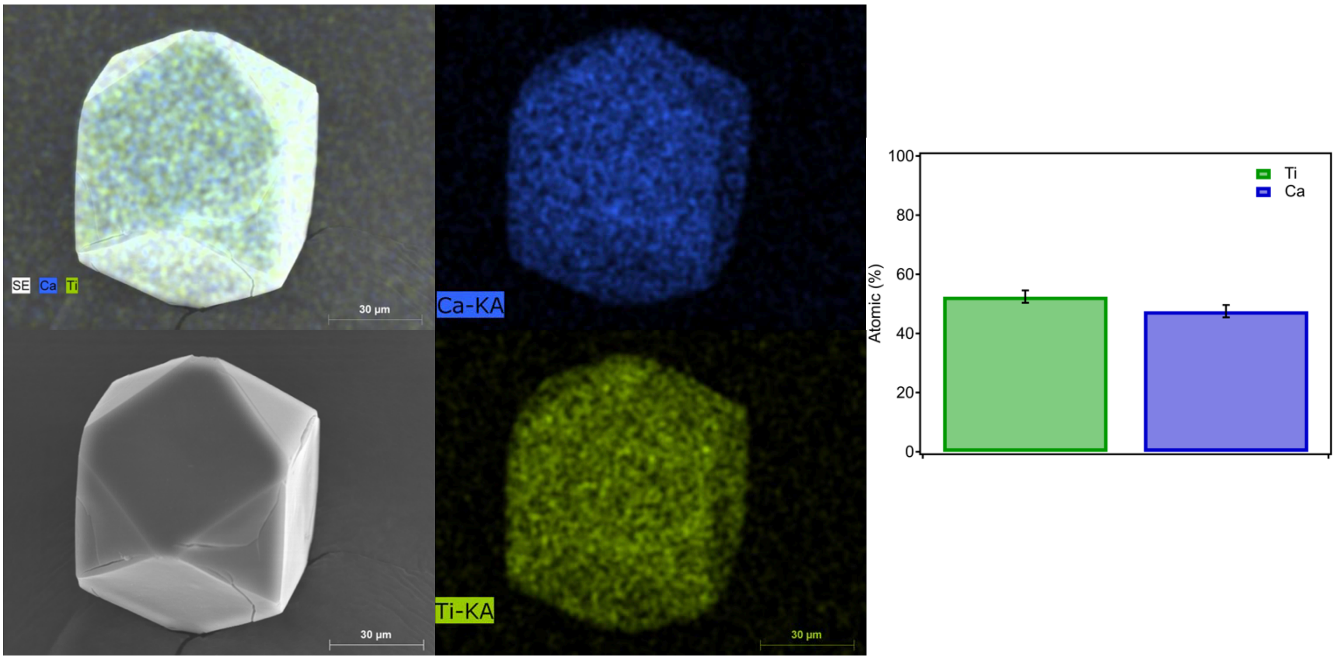

Figure S46. Mapping of **MUV-12(anth)** showing Ti (green), Ca (blue) and Experimental Ti:Ca ratio % from point and shoot EDX analysis showing the standard deviation (s) of n samples (n=3)

S.6.4. Inductively Coupled Plasma Mass Spectrometry (ICP-MS)

Inductively Coupled Plasma Mass Spectrometry (ICP-MS) measurements were performed on an Agilent 7900 instrument to confirm minimal deviations from the theoretical Ti:Ca ratios expected for TiCa cluster formation.

Table S9. Experimental metal content determined by ICP-MS

Framework	Ti [%]	Ca [%]
MUV-12	50.8 ± 0.1	49.2 ± 0.3
MUV-12(tatb)	51.0 ± 0.5	49.0 ± 0.3
MUV-12(OH)	50.9 ± 0.2	49.1 ± 0.2
MUV-12(*o*-F)$_3$	50.7 ± 0.1	49.3 ± 0.1
MUV-12(*m*-F)$_3$	50.6± 0.1	49.4 ± 0.1
MUV-12(*o*-Me)$_3$	50.5 ± 0.3	49.5 ± 0.2
MUV-12(*m*-Me)$_3$	50.7 ± 0.4	49.3 ± 0.2
MUV-12(2,6-napth)	50.6 ± 0.2	49.4 ± 0.3
MUV-12(1,4-napth)	50.4 ± 0.3	49.6 ± 0.2
MUV-12(anth)	50.4 ± 0.2	49.6 ± 0.1

S.6.5. Thermogravimetric Analysis (TGA)

TGA-SDTA curves were recorded at a ramp at a 20°C/min on a TGA 550 (Waters/TA Instruments) apparatus between 25 and 650ºC under ambient conditions (an air flow of 30 mL·min^{-1}).

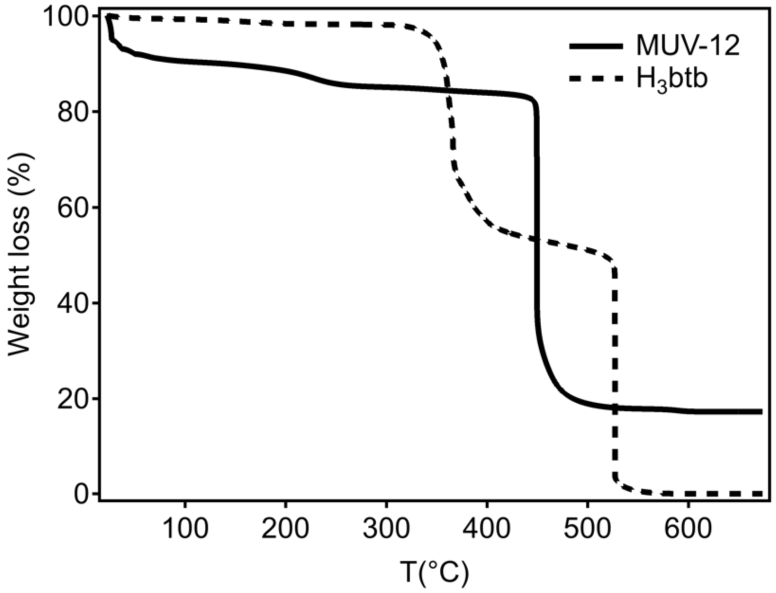

Figure S47. TGA analysis of the linker **H$_3$btb** (dashed line) and **MUV-12** (solid line)

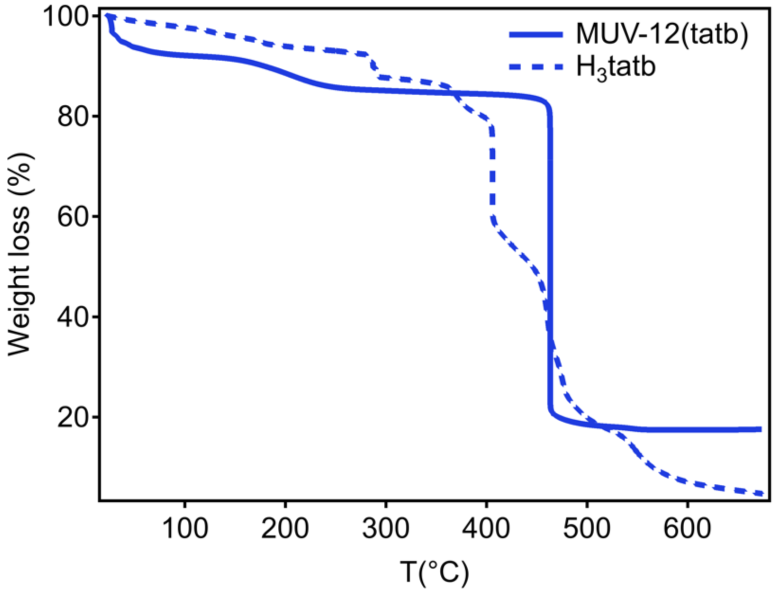

Figure S48. TGA analysis of the linker **H$_3$btb-tatb** (dashed line) and **MUV-12(tatb)** (solid line)

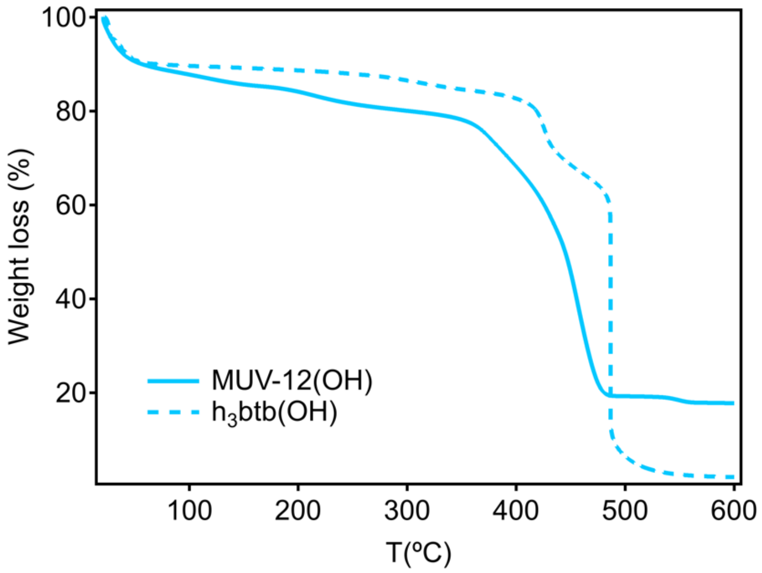

Figure S49. TGA analysis of the linker **H3btb(OH)** (dashed line) and **MUV-12(OH)** (solid line)

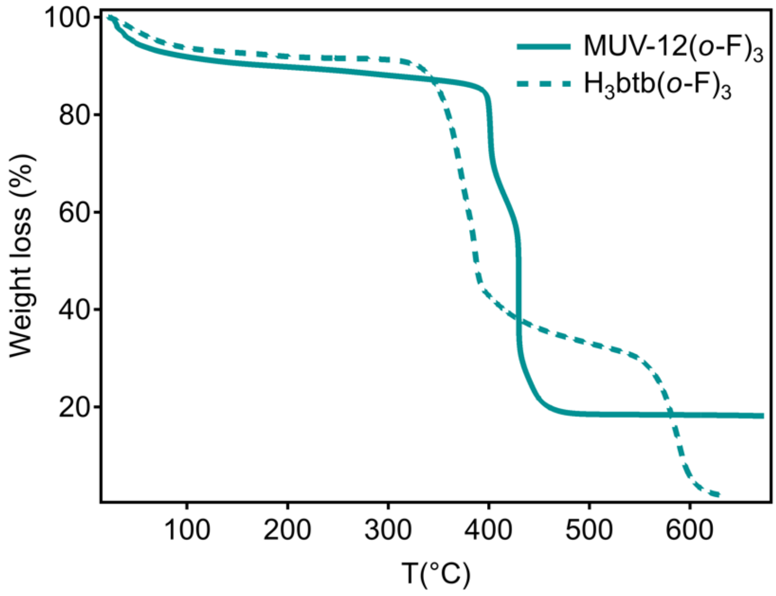

Figure S50. TGA analysis of the linker **H3btb(*o*-F)3** (dashed line) and **MUV-12(*o*-F)3** (solid line)

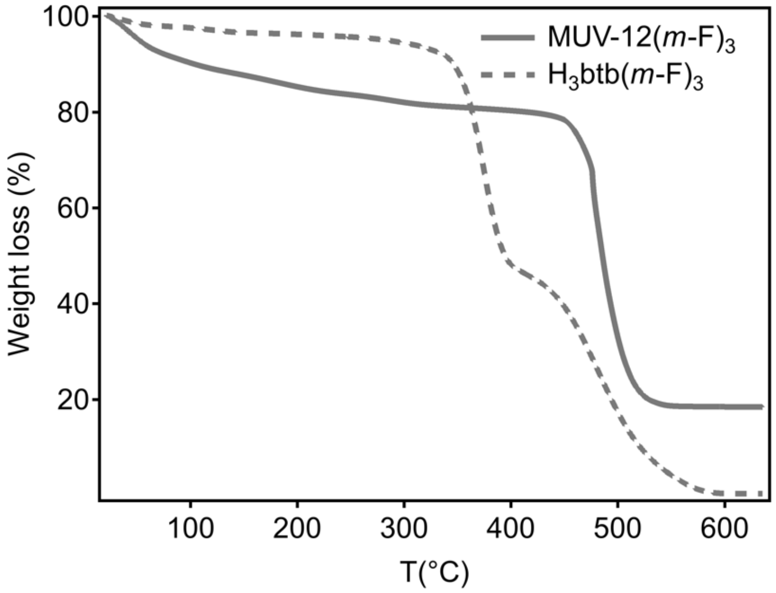

Figure S51. TGA analysis of the linker **H$_3$btb(*m*-F)$_3$** (dashed line) and **MUV-12(*m*-F)$_3$** (solid line)

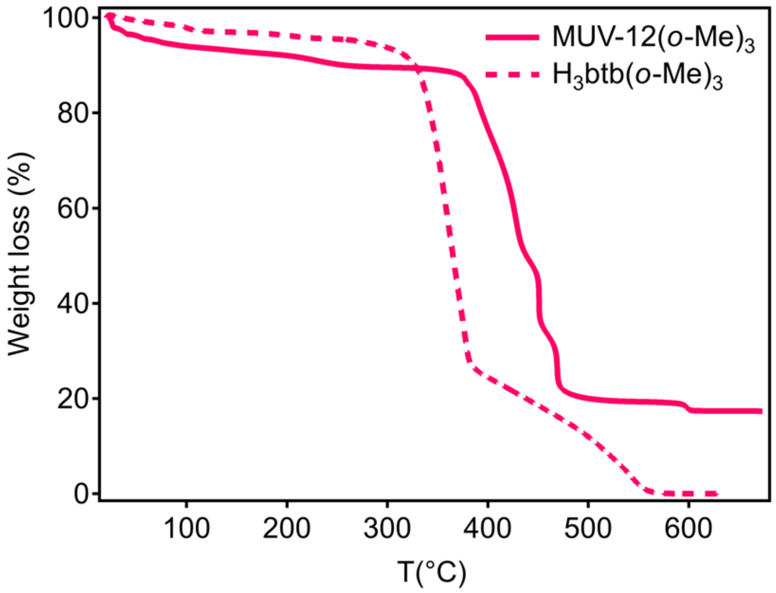

Figure S52. TGA analysis of the linker **H$_3$btb(*o*-Me)$_3$** (dashed line) and **MUV-12(*o*-Me)$_3$** (solid line)

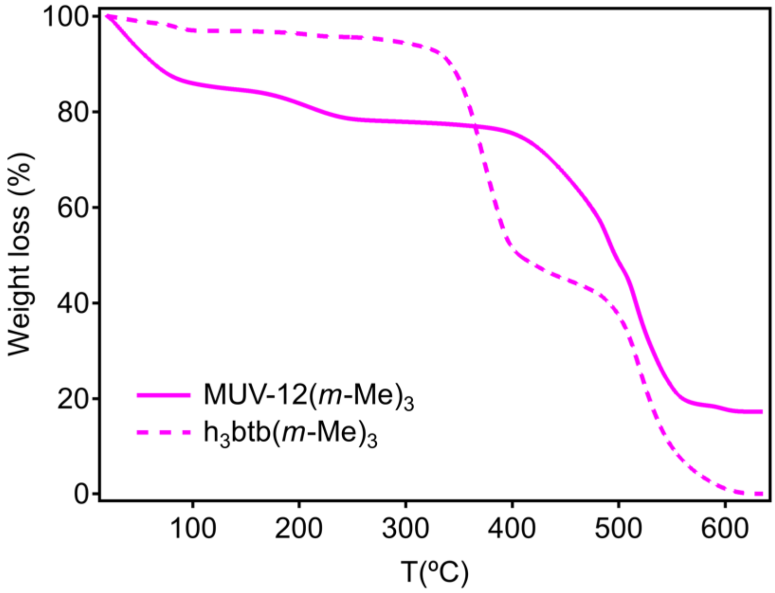

Figure S53. TGA analysis of the linker **H$_3$btb-(*m*-Me)$_3$** (dashed line) and **MUV-12(*m*-Me)$_3$** (solid line)

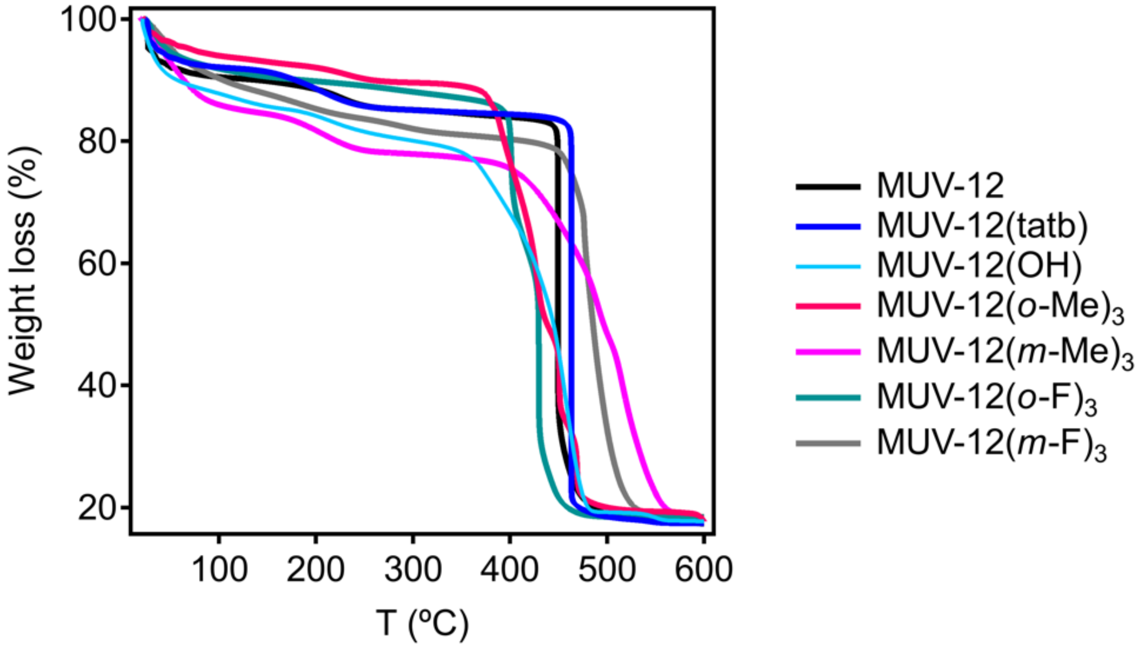

Figure S54. Comparison of the TGA analysis for **MUV-12(X)** materials

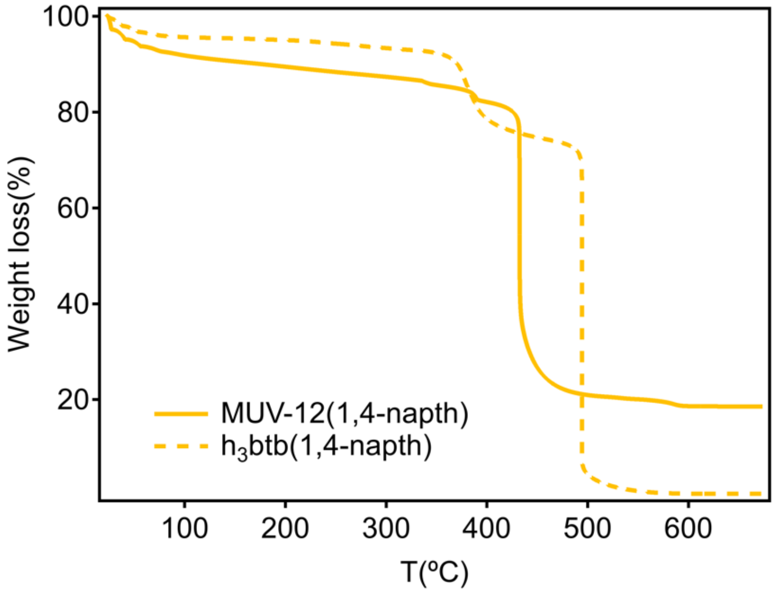

Figure S55. TGA analysis of the linker **H$_3$btb(1,4-naph)** (dashed line) and **MUV-12(1,4-naph)** (solid line)

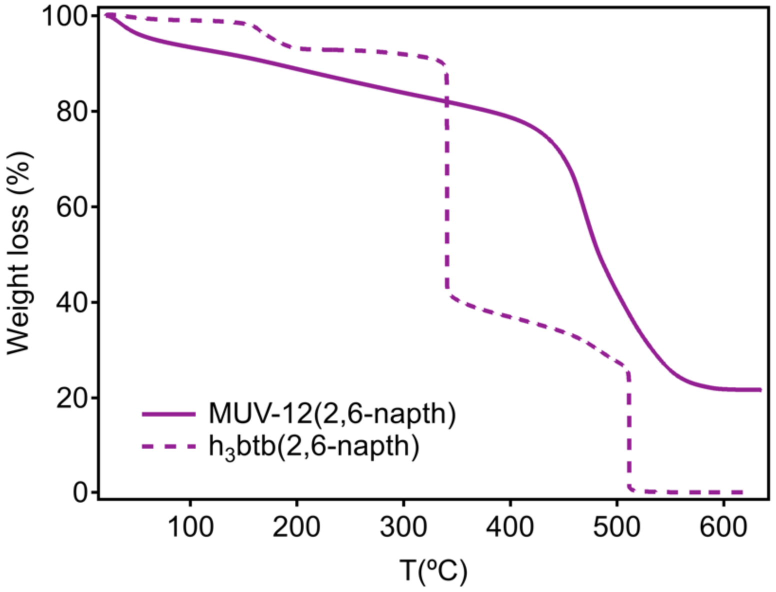

Figure S56. TGA analysis of the linker **H$_3$btb(2,6-naph)** (dashed line) and **MUV-12(2,6-naph)** (solid line).

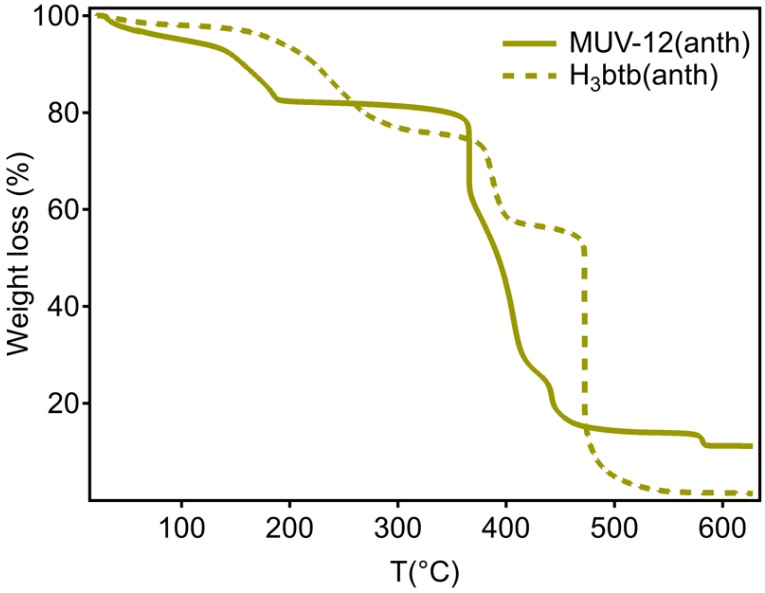

Figure S57. TGA analysis of the linker **H_3btb(anth)** (dashed line) and **MUV-12(anth)** (solid line)

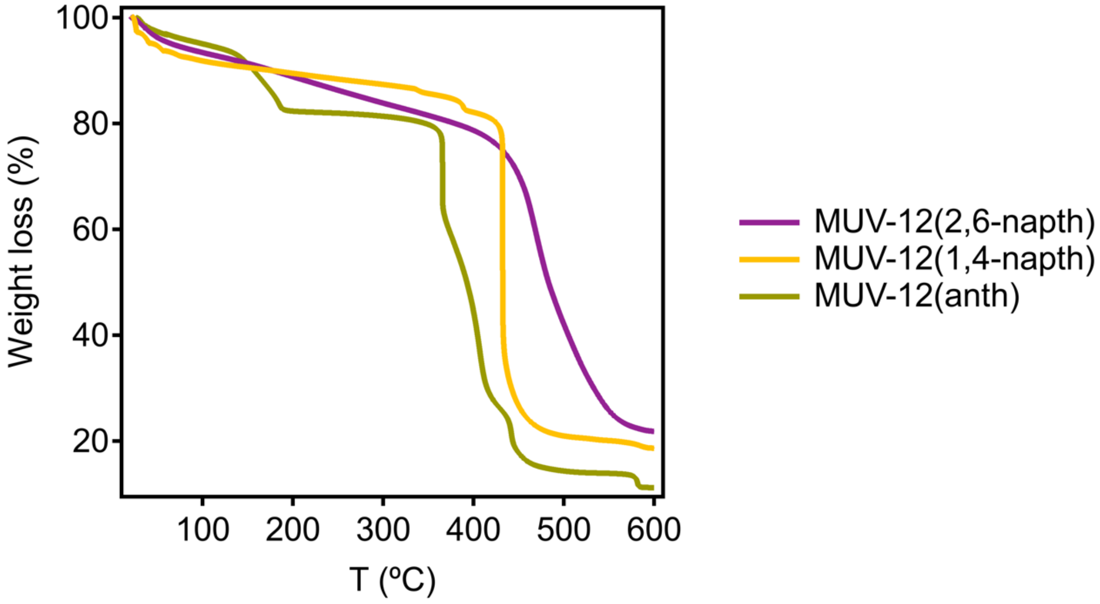

Figure S58. Comparison of the TGA analysis for **MUV-12(Y)** materials

S.6.6. UV-Vis Diffuse Reflectance Spectroscopy

UV-Vis diffuse reflectance spectroscopy (DRS) was performed on a Jasco V-670 spectrophotometer using an integrated Labsphere in the range 200-800 nm.

a) b)

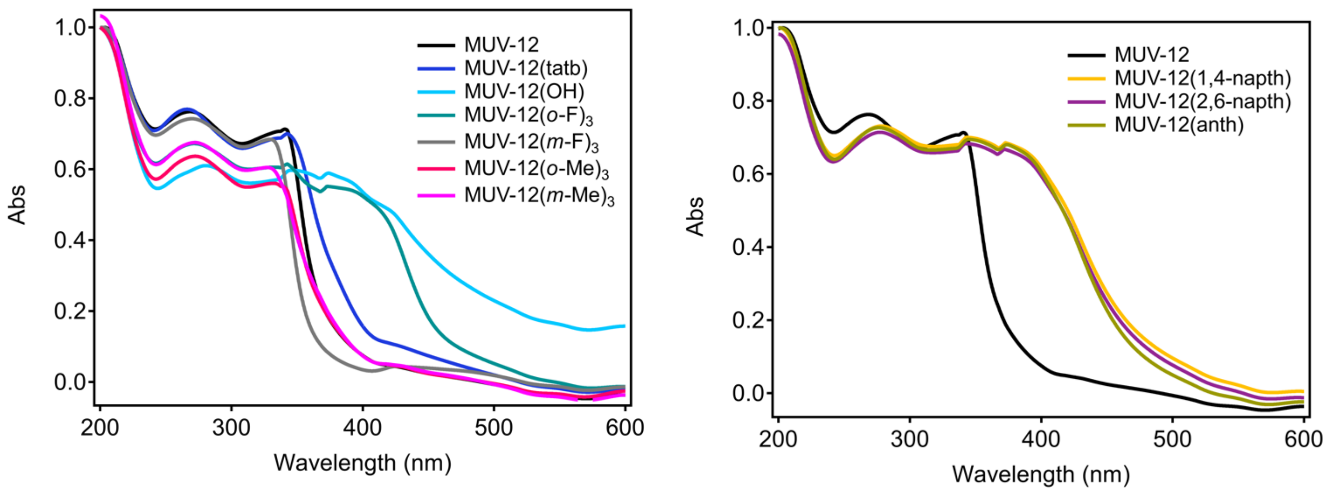

Figure S59. a) UV-Vis spectrum of all **MUV-12(X)** b) UV-Vis spectrum of all **MUV-12(Y)** and **MUV-12** as reference

<u>Experimental Optical Band-Gap Calculation</u>

a)

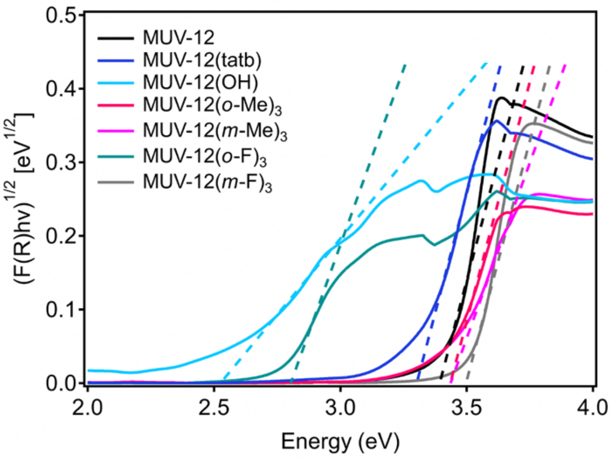

b)

Sample	Optical Band Gap (eV)
MUV-12	3.38
MUV-12(tatb)	3.30
MUV-12(OH)	2.51
MUV-12(*o*-Me)$_3$	3.46
MUV-12 (*m*-Me)$_3$	3.46
MUV-12(*o*-F)$_3$	2.80
MUV-12(*m*-F)$_3$	3.50

Figure S60. a) Tauc plot ([F(R) x $h\nu$]^2 vs $h\nu$]^2 for bandgap energy (E_g) for MUV-12(X) samples. The dotted line corresponds to the regression fitting of the linear part of the plot, b) Summary of experimental optical band-gap values calculated by Tauc method

a)

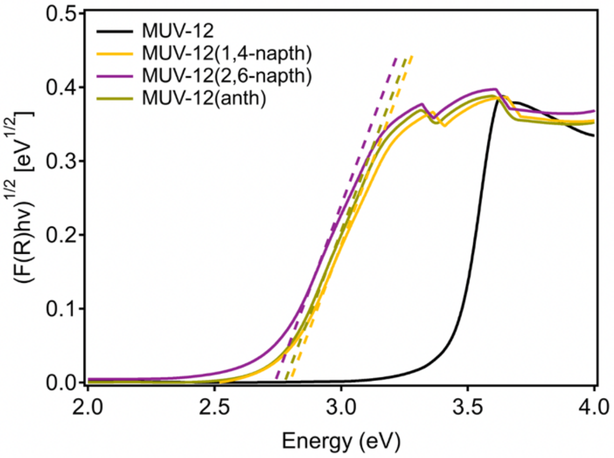

b)

Sample	Optical Band Gap (eV)
MUV-12(1,4-napth)	2.80
MUV-12(2,6-napth)	2.75
MUV-12(anth)	2.78

Figure S61. a) Tauc plot ([F(R) x hv]^2 vs hv]^2 for bandgap energy (E_g) for MUV-12(Y) samples and MUV-12 as reference. The dotted line corresponds to the regression fitting of the linear part of the plot. b) Summary of experimental optical band-gap values calculated by Tauc method

The band gap was determined from diffuse reflectance data using the Tauc Method,[14] based on the assumption that the energy-dependent absorption coefficient α can be expressed by the following equation ***(1)***:

$$(\alpha \cdot hv)^{1/\gamma} = B(hv - E_g) \qquad \textbf{\textit{Equation (1)}}$$

where h is the Planck constant, v is the photon's frequency, E_g is the band gap energy, and B is a constant. Figure S60 and S61 shows the corresponding reflectance spectrums transformed according to eq 1 plotted against the photon energy. The *x*-axis intersection point of the linear fit of the Tauc plot gives an estimate of the band gap energy.

S.7. CHEMICAL STABILITY

To test the chemical stability of these frameworks, we used ICP-MS to analyzed the supernatants after incubation of **MUV-12(X)** and **MUV-12(Y)** materials in water for 24 hours at pH = 7 under static conditions. For each material, exactly 10.0 mg have been suspended in 3.0 mL of ultrapure water. The leaching in all **MUV-12(X)** and **MUV-12(Y)** materials display negligible leaching confirming high chemical stability.

Table S10. Metal concentrations in solution of **MUV-12(X)** and **MUV-12(Y)** after soaking in neutral media after 24 hours

Framework	[Ti](mg/L)
MUV-12	0.0121 ± 0.002
MUV-12(tatb)	0.0189 ± 0.002
MUV-12(OH)	0.0197 ± 0.003
MUV-12(*o*-F)$_3$	0.0202 ± 0.002
MUV-12(*m*-F)$_3$	0.0214 ± 0.003
MUV-12(*o*-Me)$_3$	0.0231 ± 0.002
MUV-12(*m*-Me)$_3$	0.0269 ± 0.004
MUV-12(2,6-napth)	0.0463 ± 0.003
MUV-12(1,4-napth)	0.0689 ± 0.004
MUV-12(anth)	0.0743 ± 0.002

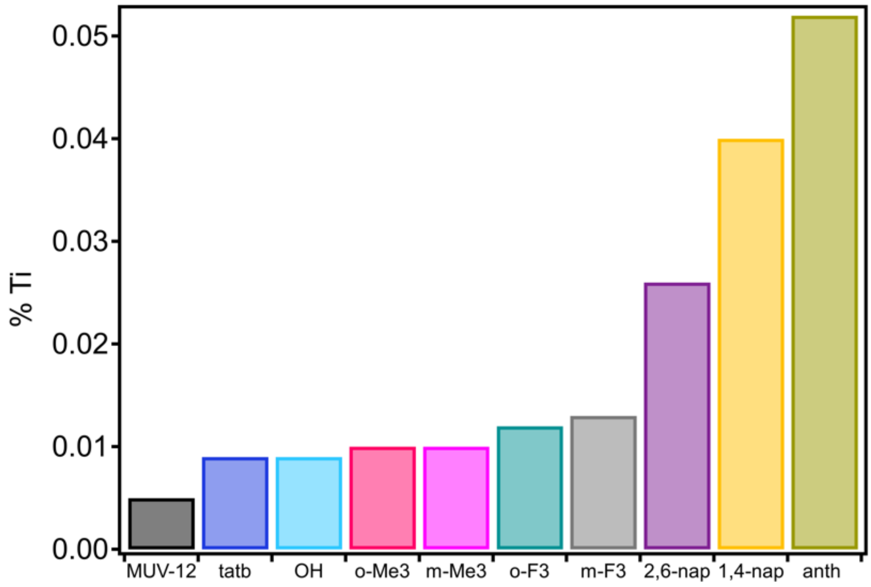

Figure S62. Percentage of Ti leached into the supernatants determined by ICP-MS after soaking **MUV-12(X)** and **MUV-12(Y)** materials at pH 7 for 24 hours

S.8. ELECTRON PARAMAGNETIC RESONANCE (EPR)

EPR measurements were performed on 20 mg of a solid suspended in freshly distilled deoxygenated MeCN. The samples were degassed prior to experiments by a freeze-pump-thaw procedure and the tube was subsequently flame-sealed. The samples were irradiated overnight with a Kessil lamp (PR-160L, 440 nm, 100% LED intensity) at 0.5-1 cm from the tube. EPR data was recorded in a Bruker ELEXYS E580 spectrometer under X-band irradiation (~9.3 GHz) at 77 K.

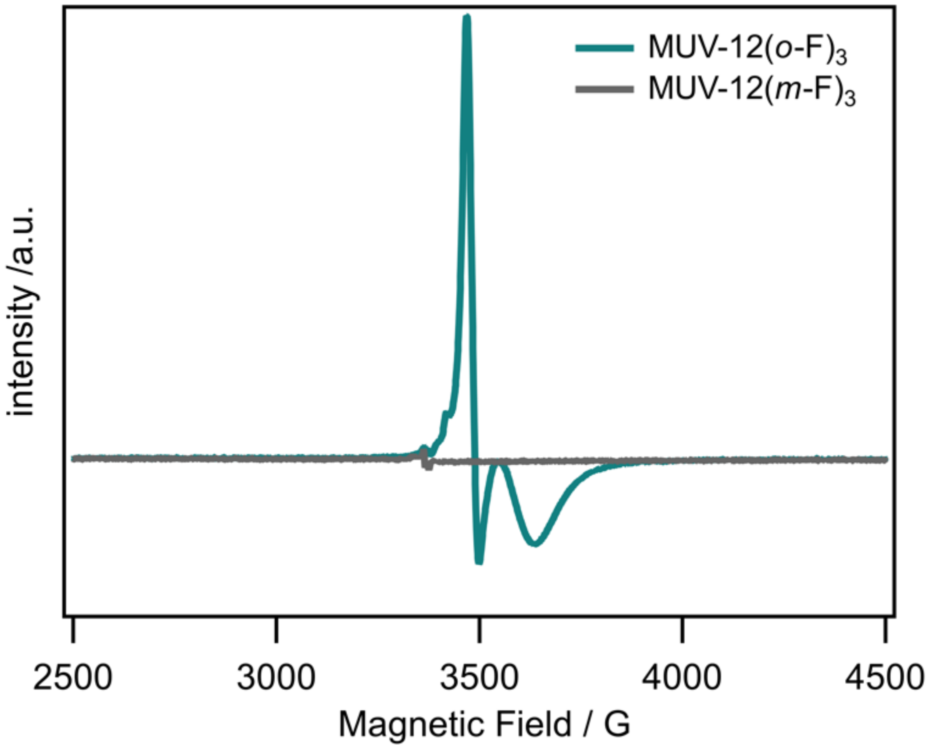

Figure S63. EPR measurements of **MUV-12(*o*-F)$_3$** and **MUV-12*(m*-F)$_3$**

S.9. COMPUTATIONAL METHODS

The reported structure of **MUV-12** (CCDC: 2018540) was used as initial structure and modified with the Materials Studio (MS) 2017 R2. The final models, corresponding to the interpenetrated (IP) and non-interpenetrated (INP) phases, were optimized with Vienna Ab initio Simulation Package (VASP).[15,16] For the geometry optimization and lattice constants we used the generalized gradient approximation (GGA) with the Perdew–Burke–Ernzerhof (PBE) functional,[17] and including van der Waals (vdW) corrections via the DFT-D3 method of Grimme.[18,19] The projector augmented wave (PAW) method[20] was used to describe the frozen core electrons and their interaction with the valence electrons. The kinetic energy cut-off for the plane-wave basis set expansion was chosen as 500 eV, and a Γ-points was used for integrations in the reciprocal space, due to the large size of the unit cell of the direct lattice.

Table S11. Cell parameters (Å) from Rietveld refinements and calculated DFT, $\Delta E_{IP\text{-}NIP}$ energy differences (kJ/mol) of the interpenetrated (IP) and non-interpenetrated (NIP) phases and the centroid-to-centroid distances (Å) separating the catenated net

Name	a(Exp) (IP/NIP)	NIP	IP	$\Delta E_{IP\text{-}NIP}$	centroid distance
		a(Cal)	a(Cal)		
**MUV-12**	26.401 **(IP)**	26.401	26.411	-628	3.61
**MUV-12(tatb)**	26.131 **(IP)**	26.105	26.105	-668	3.60
**MUV-12(OH)**	26.403 **(IP)**	26.419	26.403	-350	3.66
**MUV-12(o-Me)$_3$**	26.500 **(IP)**	26.265	26.258	-713	3.61
**MUV-12(m-Me)$_3$**	26.458 **(IP)**	26.376	26.389	-705	3.48
**MUV-12(o-F)$_3$**	26.532 **(IP)**	26.339	26.441	-674	3.60
**MUV-12(m-F)$_3$**	26.465 **(IP)**	26.399	26.353	-642	3.61
**MUV-12(2,6-naph)**	-	31.315	31.561	-511	3.88
**MUV-12(1,4-naph)**	26.448 **(NIP)**	26.398	26.516	53	3.97
**MUV-12(anth)**	26.127 **(NIP)**	26.158	26.173	367	4.10

S.10. NMR SPECTRA

Compound 1:

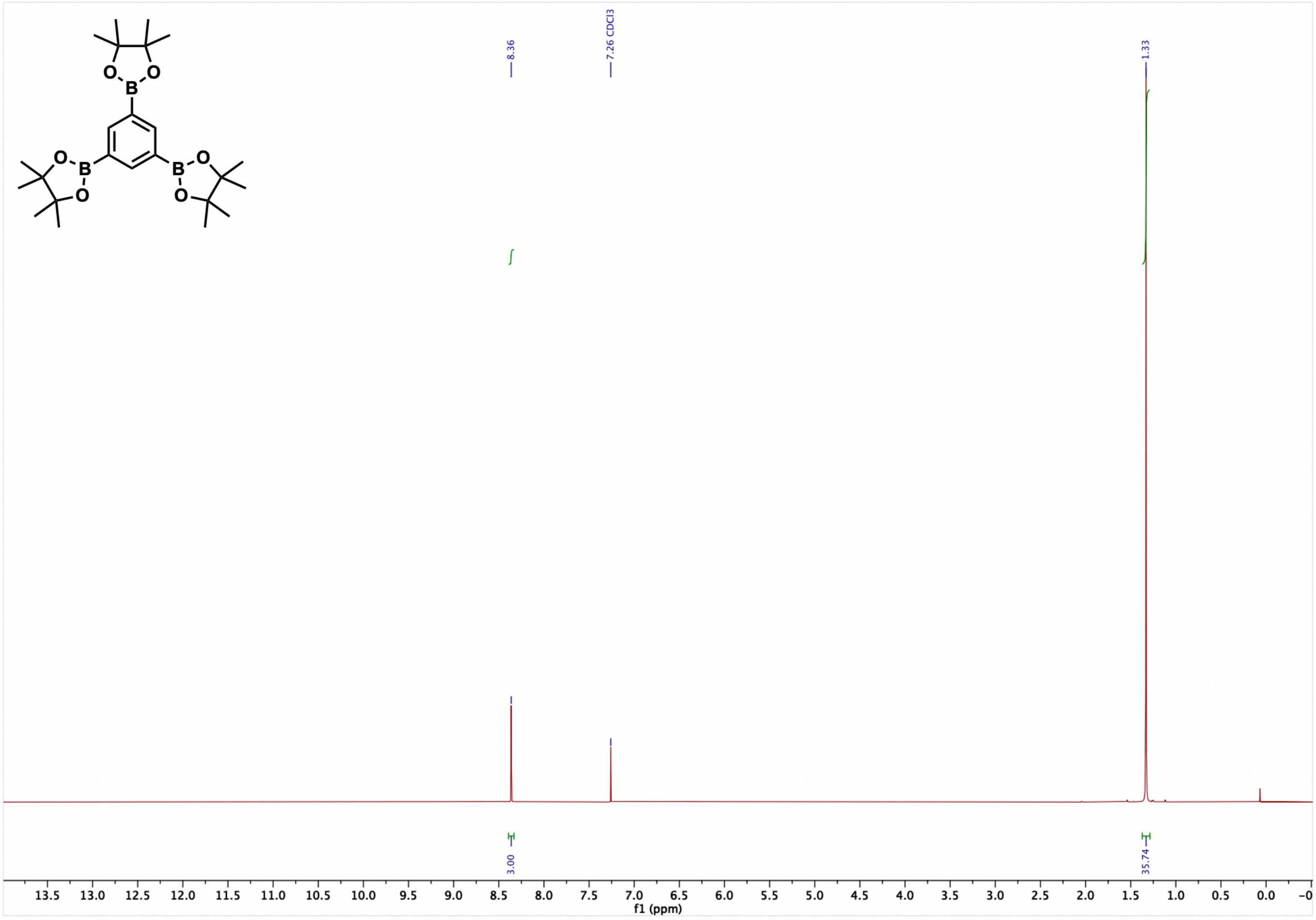

Figure 64: ^{1}H NMR spectrum of compound **1** in $CDCl_3$

Compound 2:

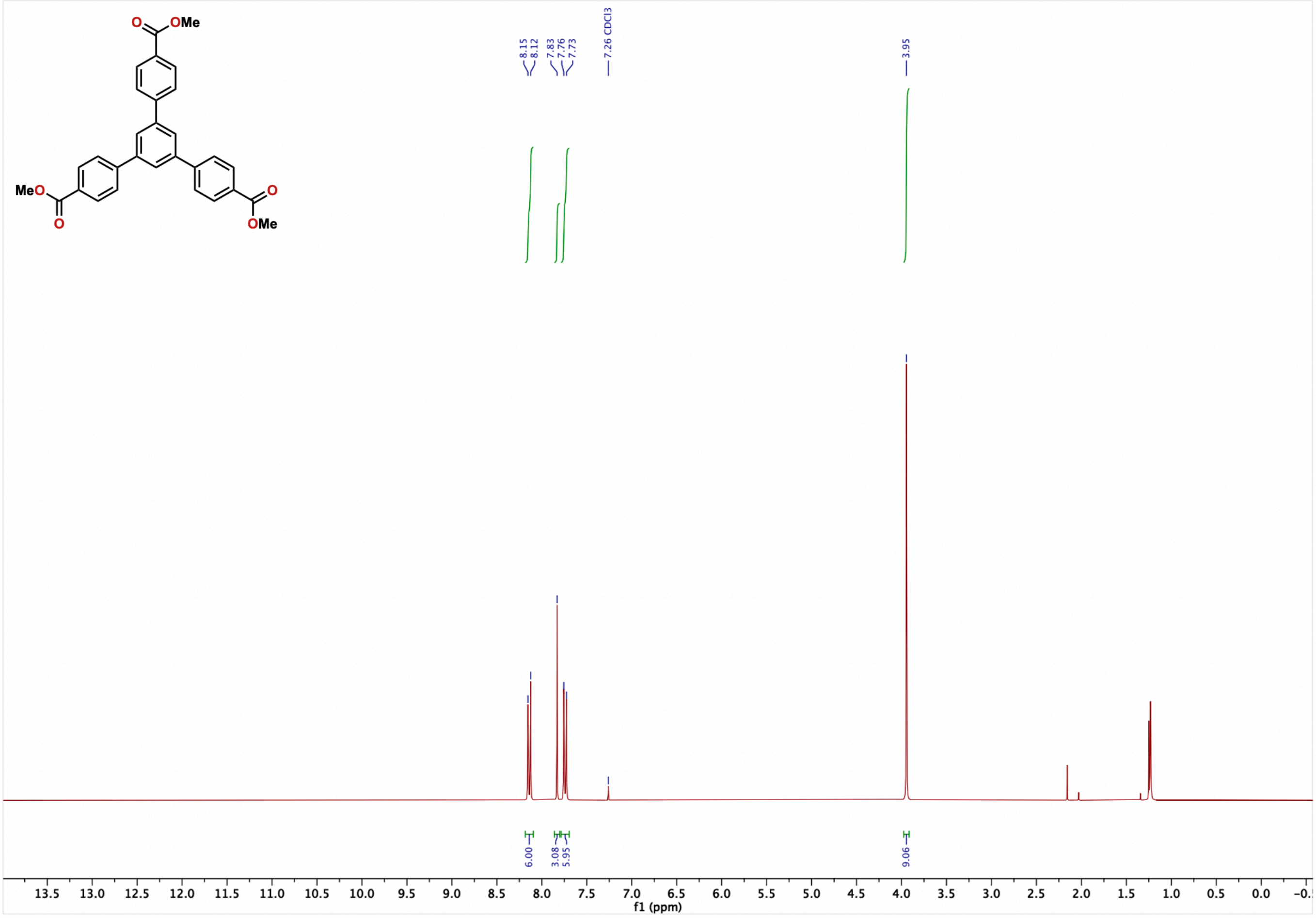

Figure 65: ^{1}H NMR spectrum of compound **2** in $CDCl_3$

Compound 3:

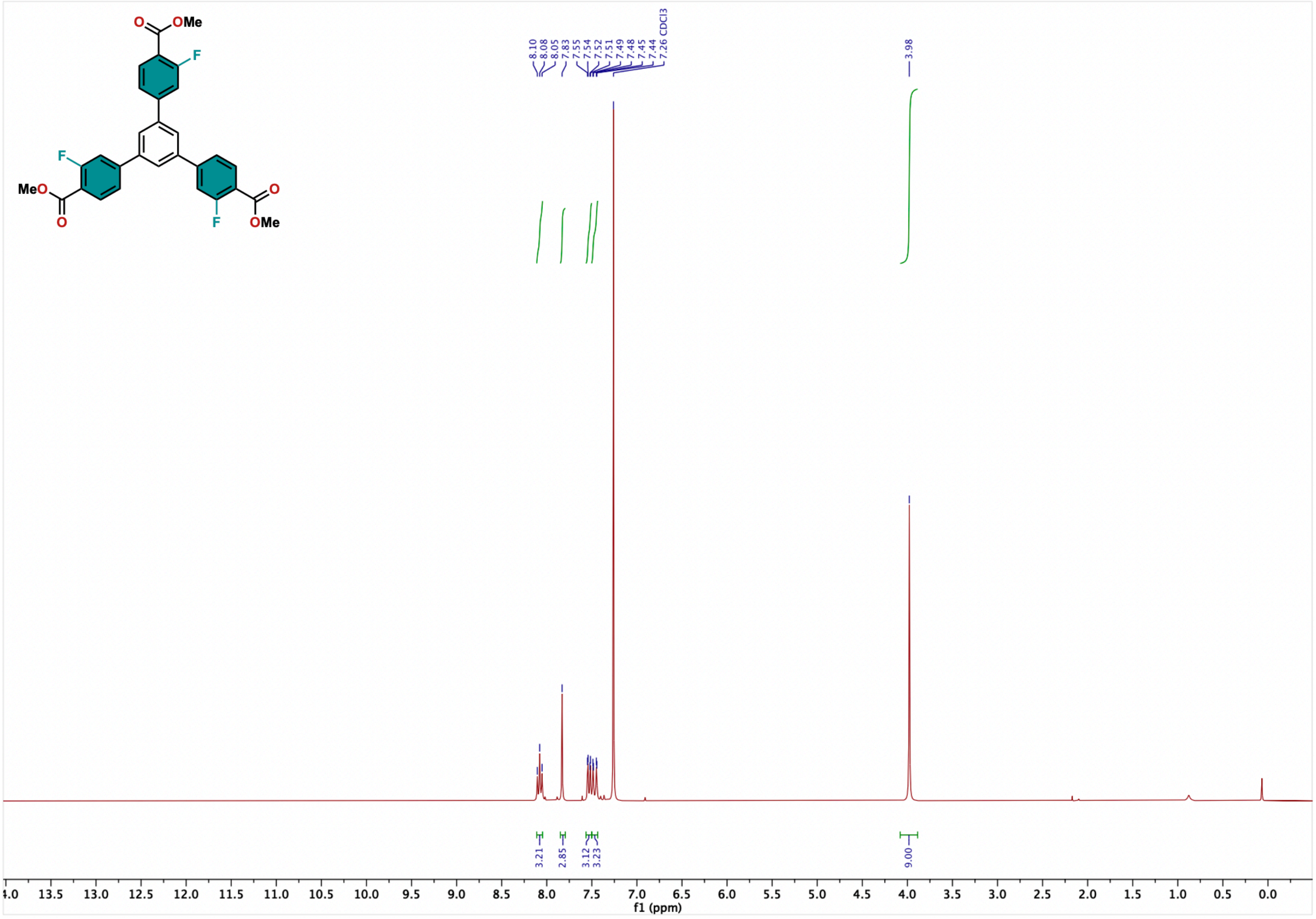

Figure 66: ^{1}H NMR spectrum of compound **3** in $CDCl_3$

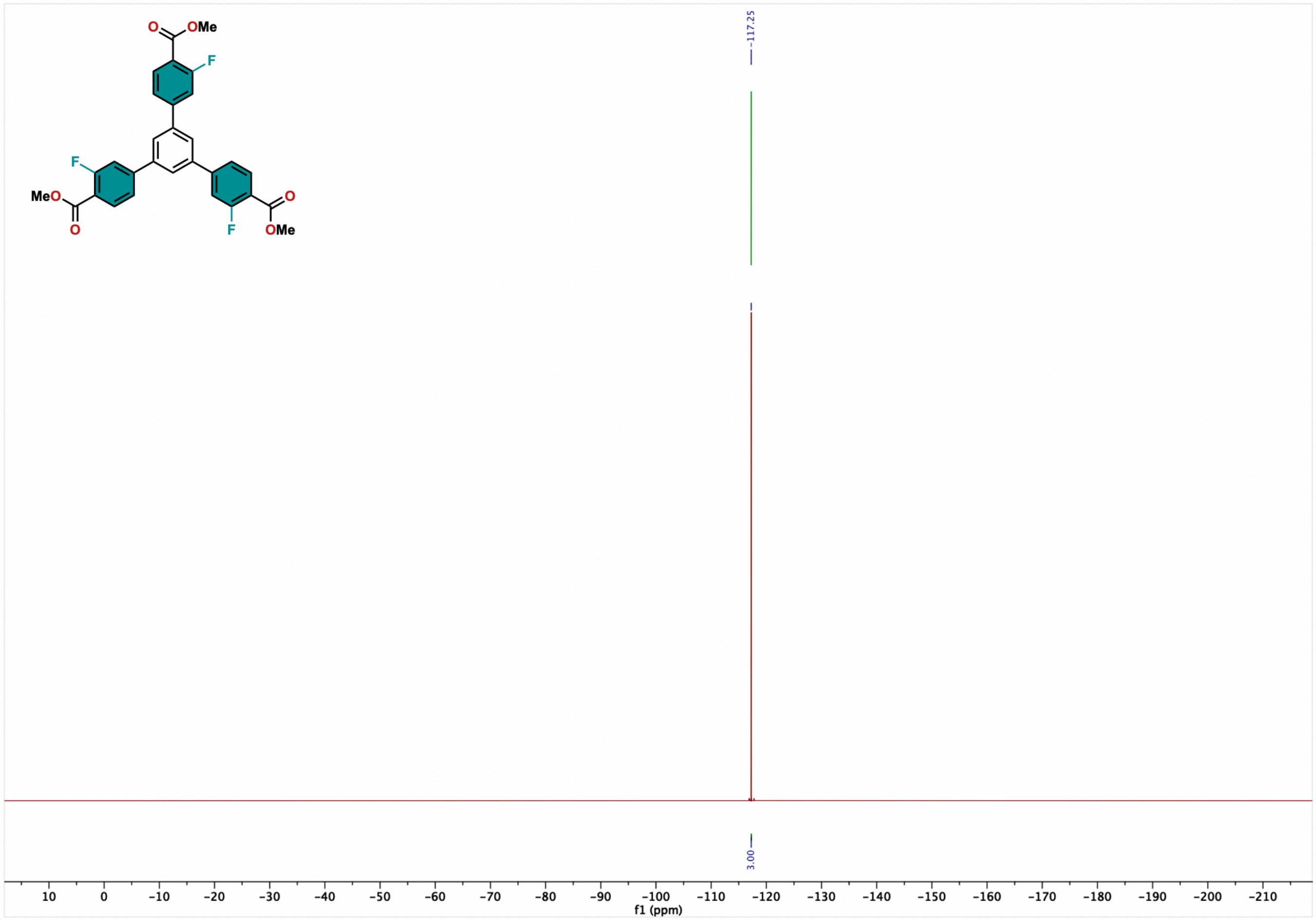

Figure 67: ^{19}F NMR spectrum of compound **3** in $CDCl_3$

Compound 4:

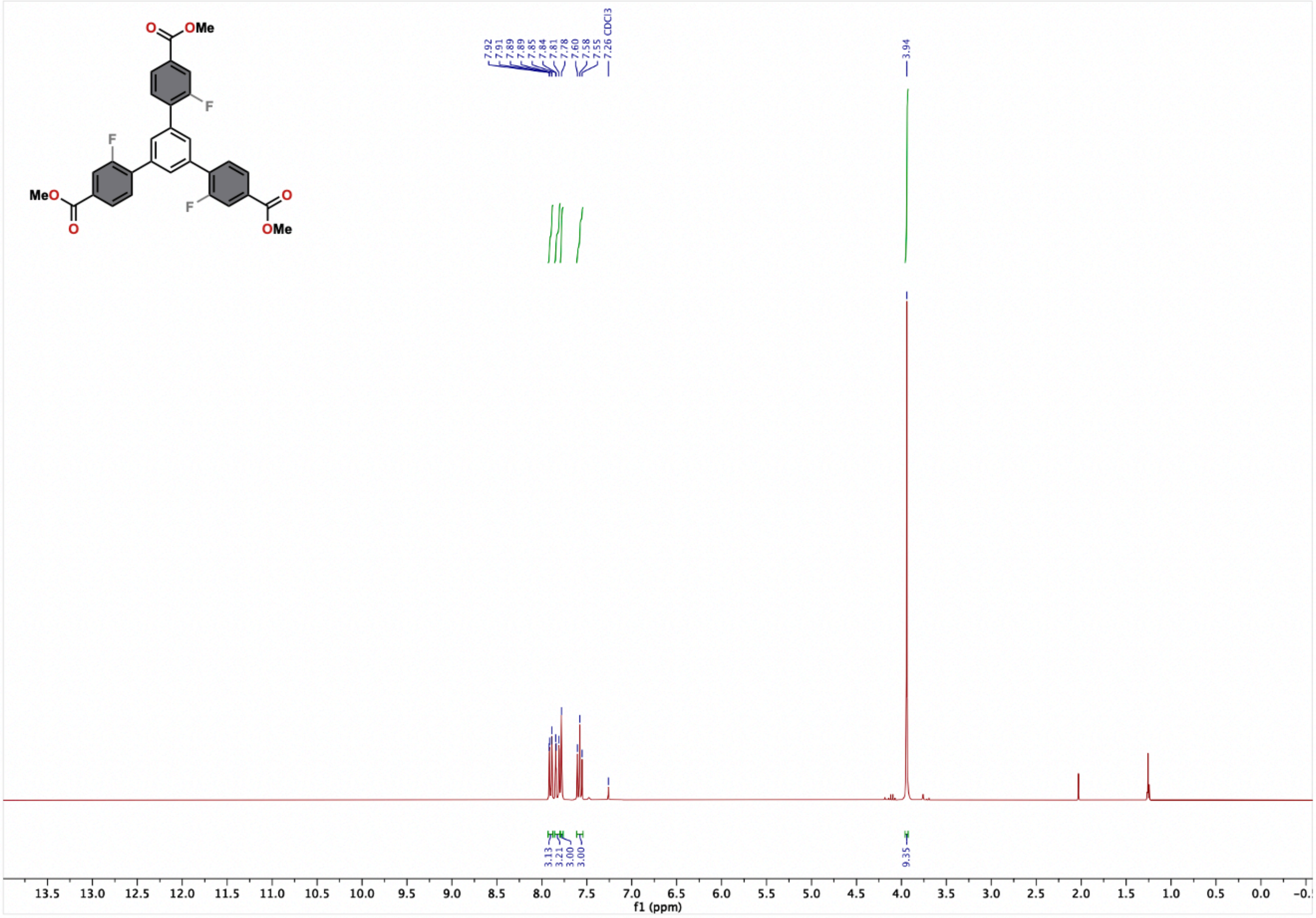

Figure 68: ^{1}H NMR spectrum of compound **4** in $CDCl_3$

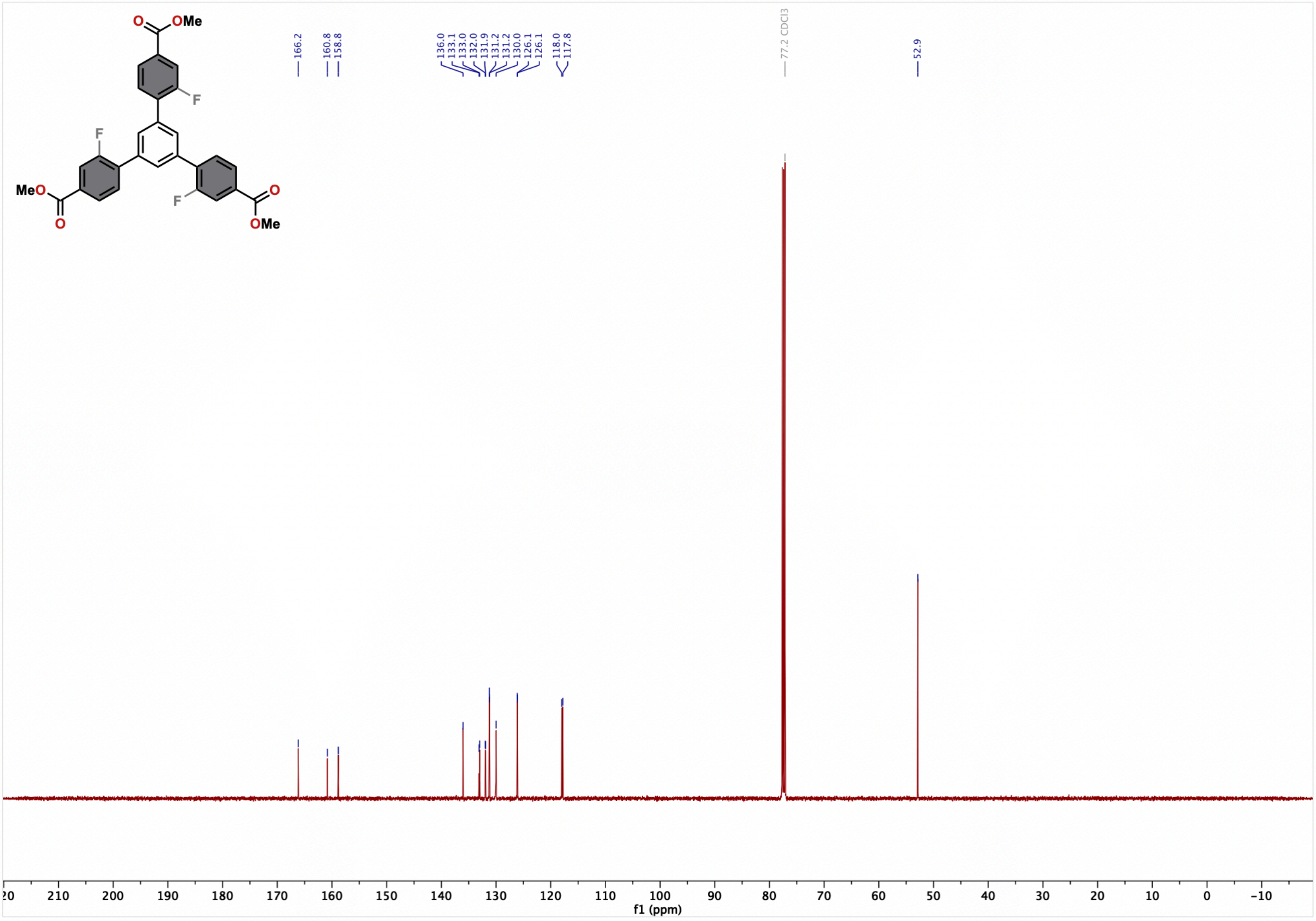

Figure 69: ^{13}C NMR spectrum of compound **4** in $CDCl_3$

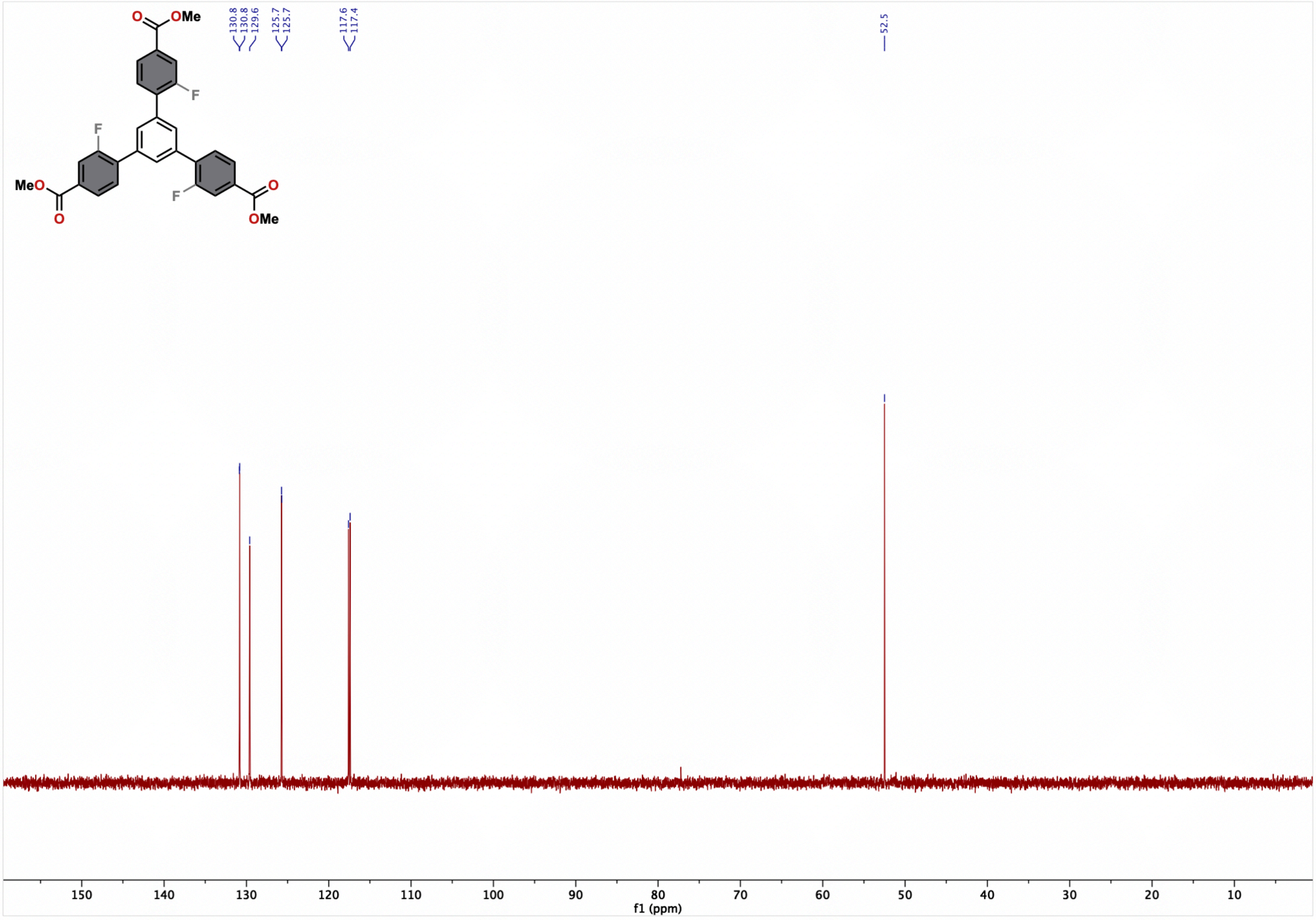

Figure 70: DEPT-135 NMR spectrum of compound **4** in $CDCl_3$

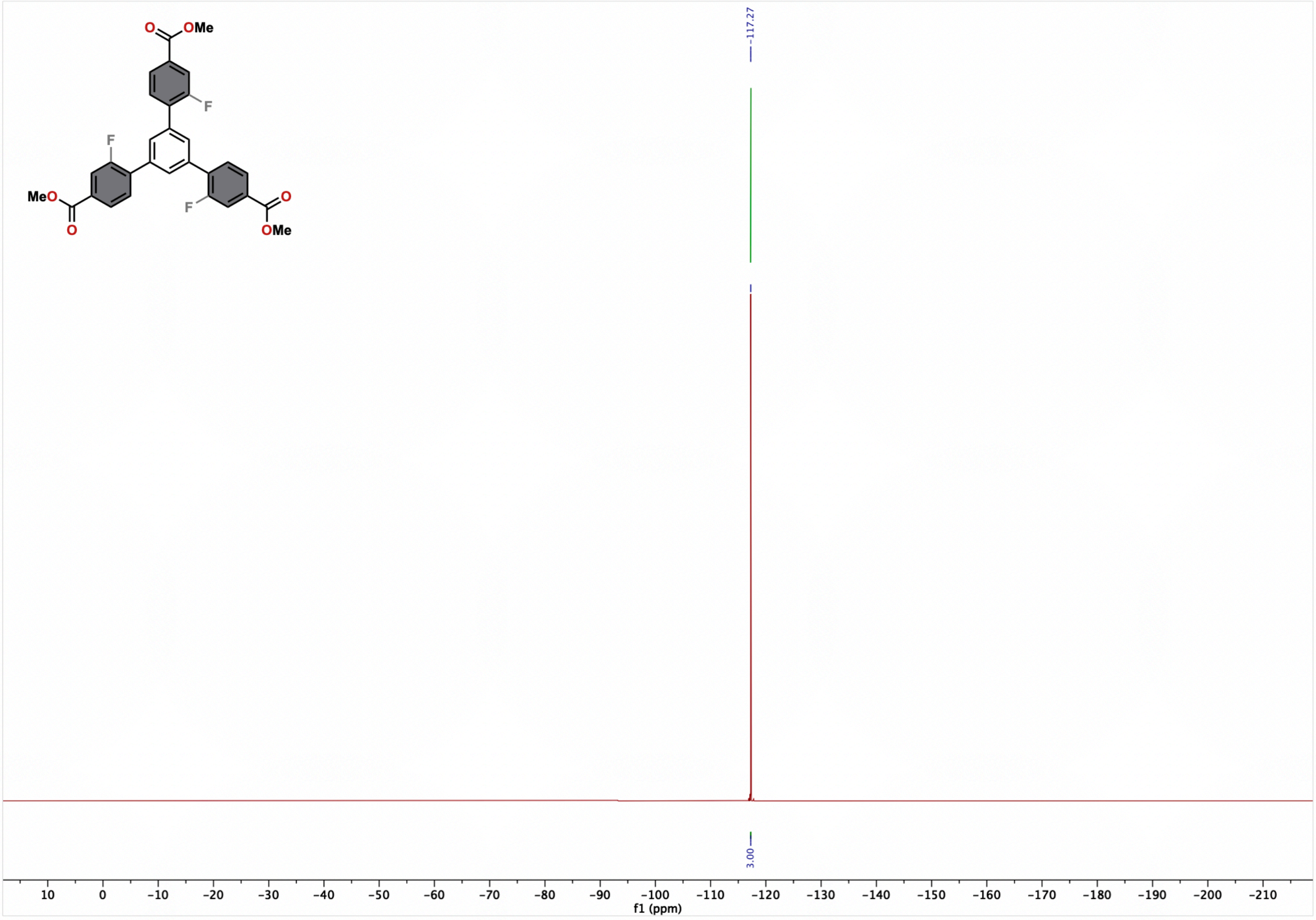

Figure 71: ^{19}F NMR spectrum of compound **4** in $CDCl_3$

Compound 5:

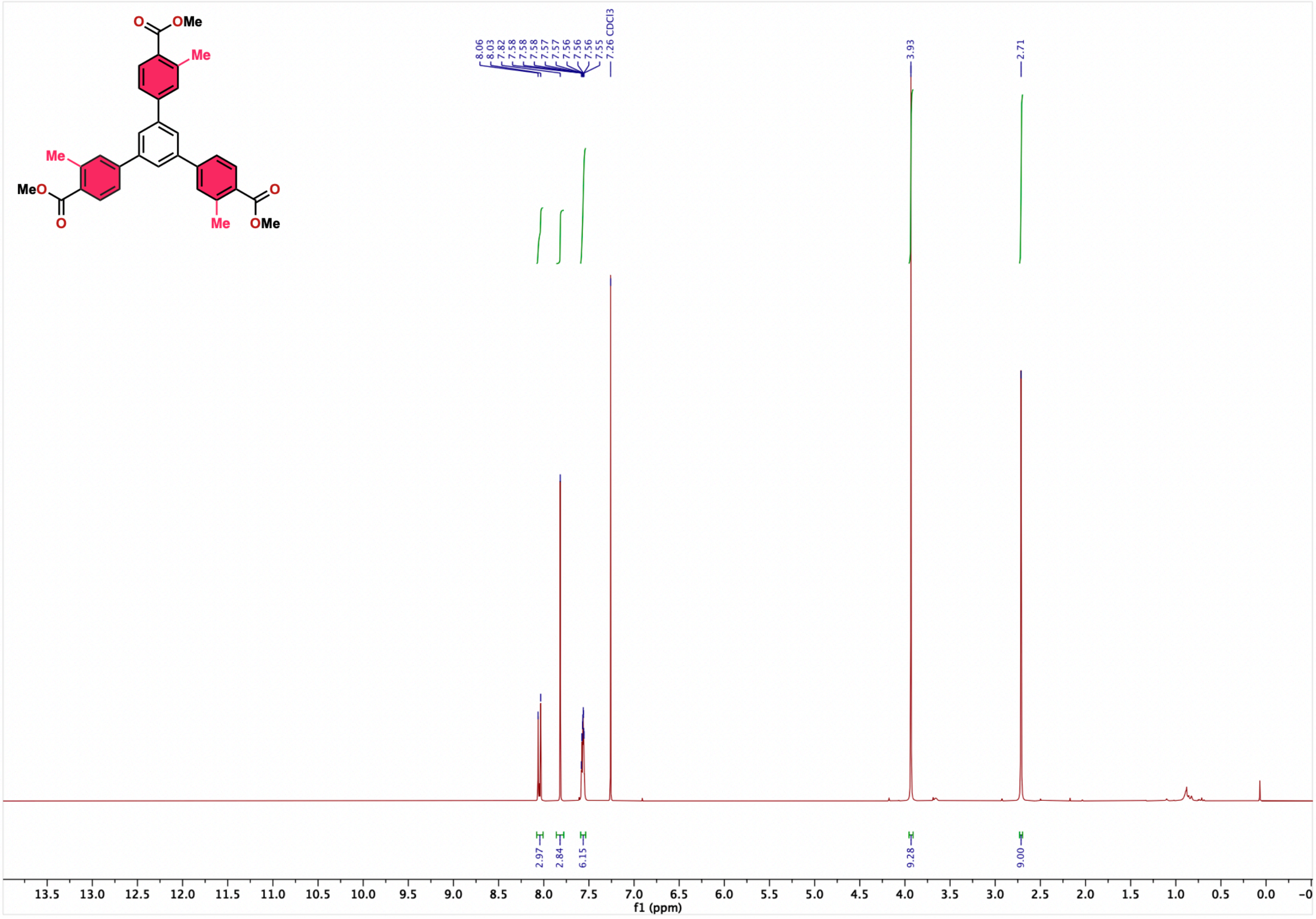

Figure 72: ^{1}H NMR spectrum of compound **5** in $CDCl_3$

Compound 6:

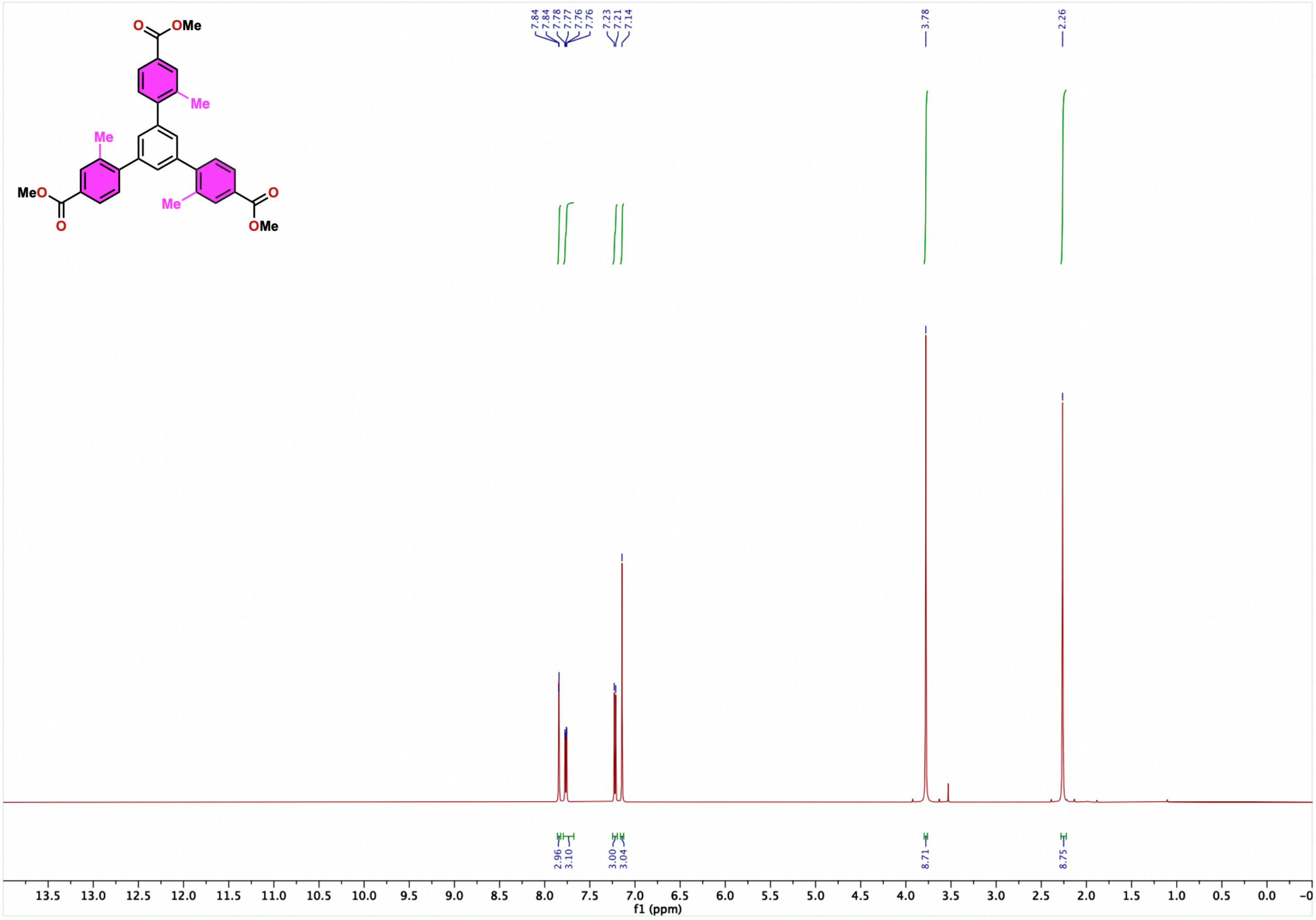

Figure 73: ^{1}H NMR spectrum of compound **6** in $CDCl_3$

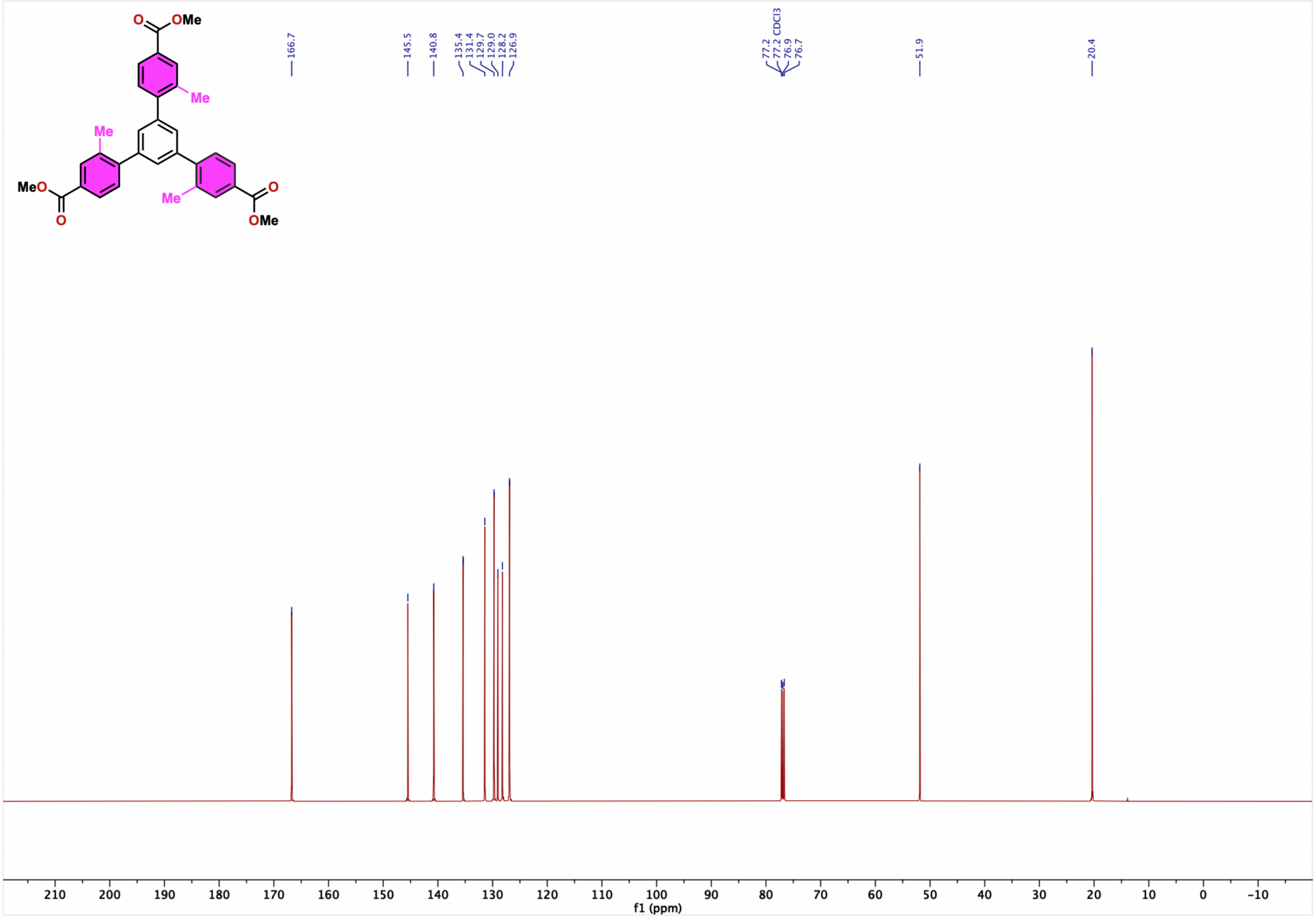

Figure 74: ^{13}C NMR spectrum of compound **6** in $CDCl_3$

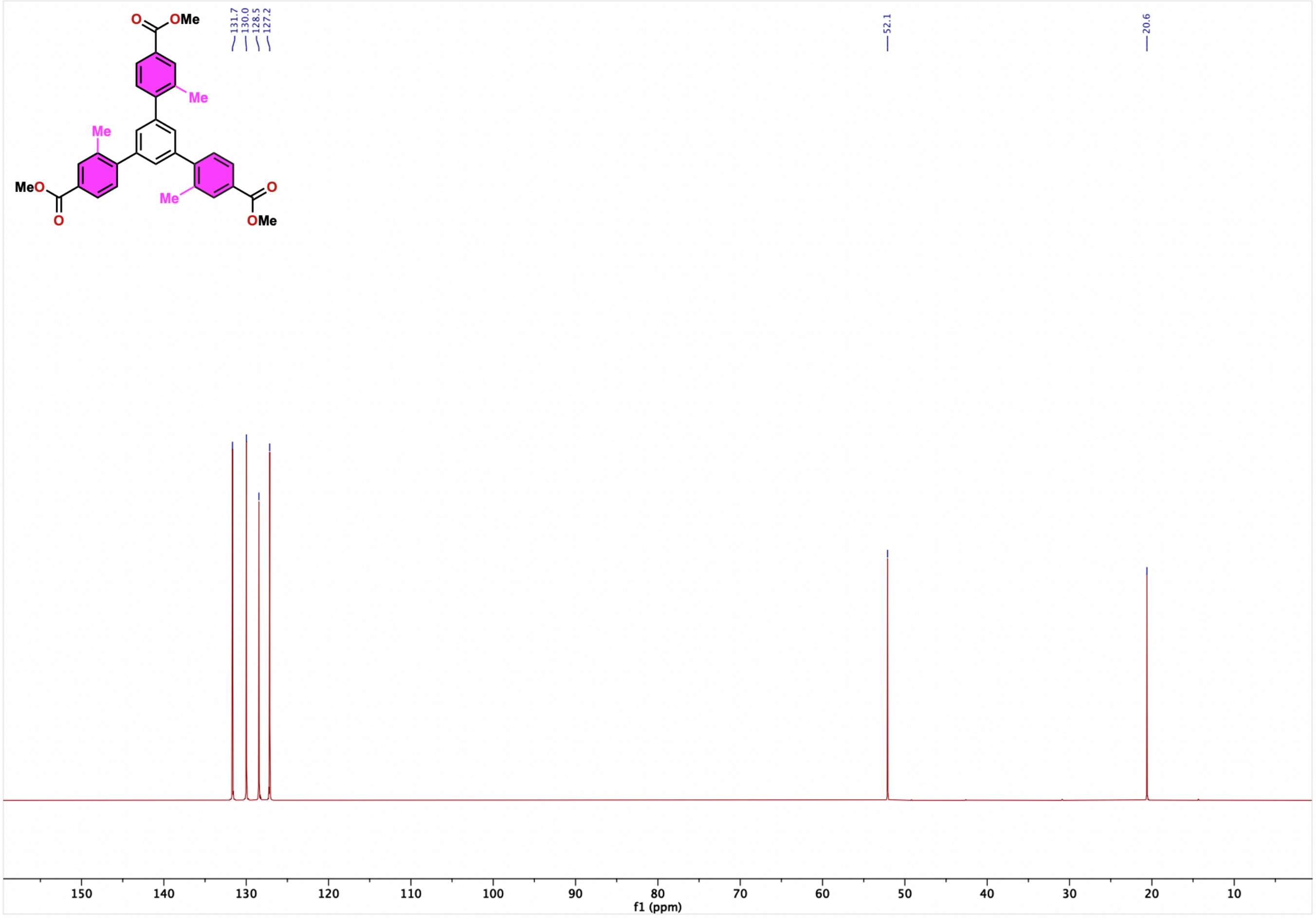

Figure 75: DEPT-135 NMR spectrum of compound **6** in $CDCl_3$

Compound 7:

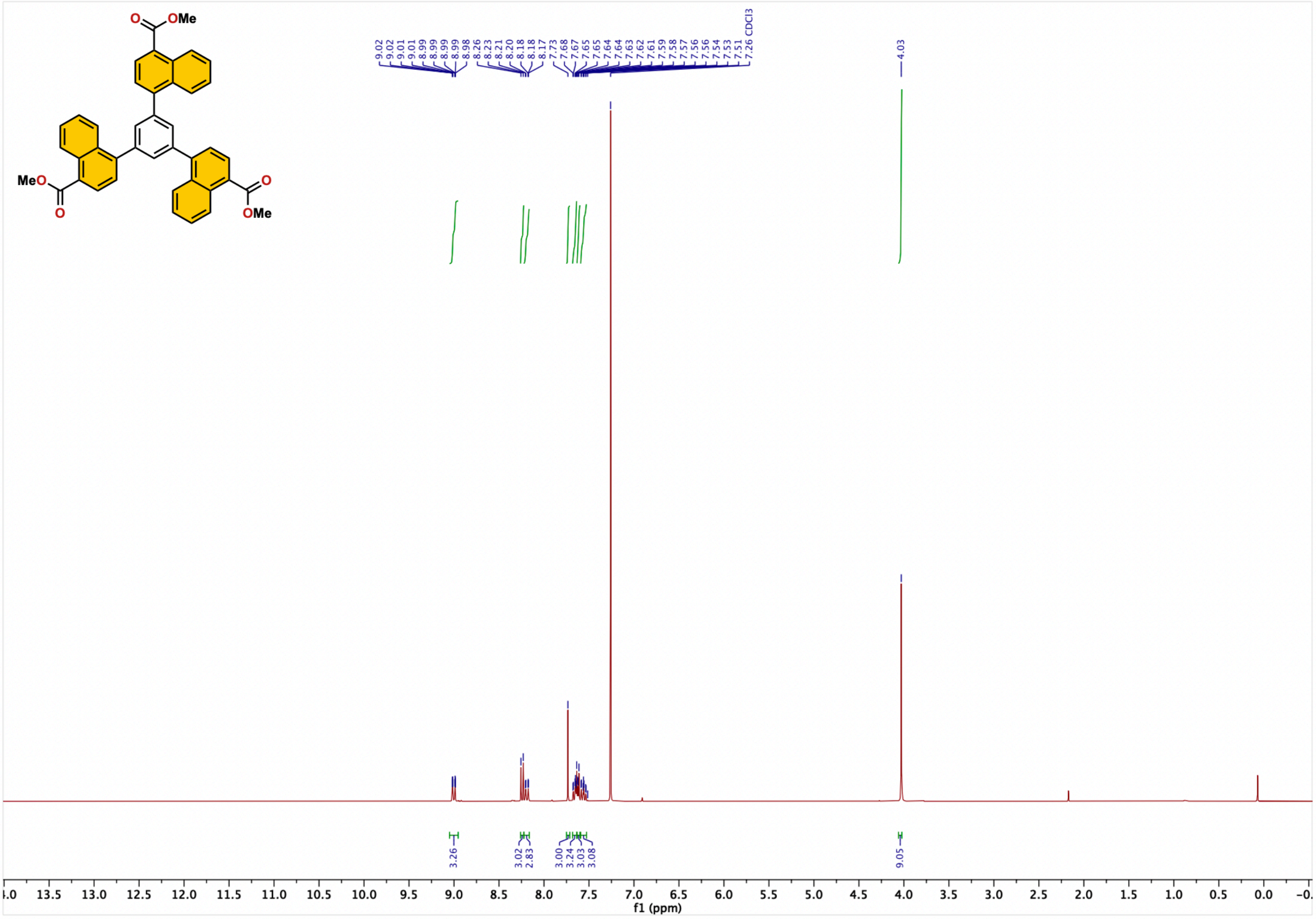

Figure 76: ^{1}H NMR spectrum of compound **7** in $CDCl_3$

Compound 8:

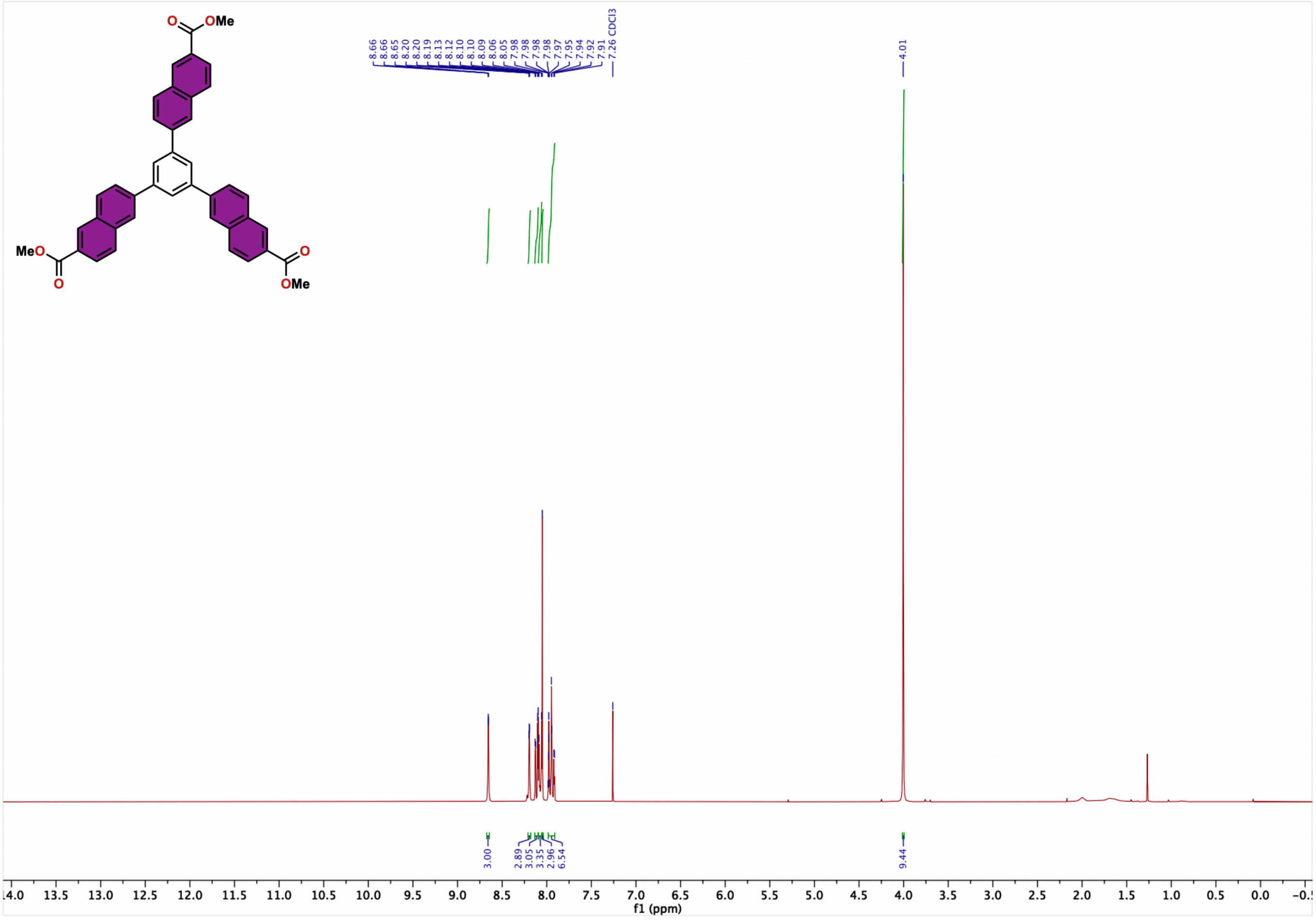

Figure 77: ^{1}H NMR spectrum of compound **8** in $CDCl_3$

Compound 9:

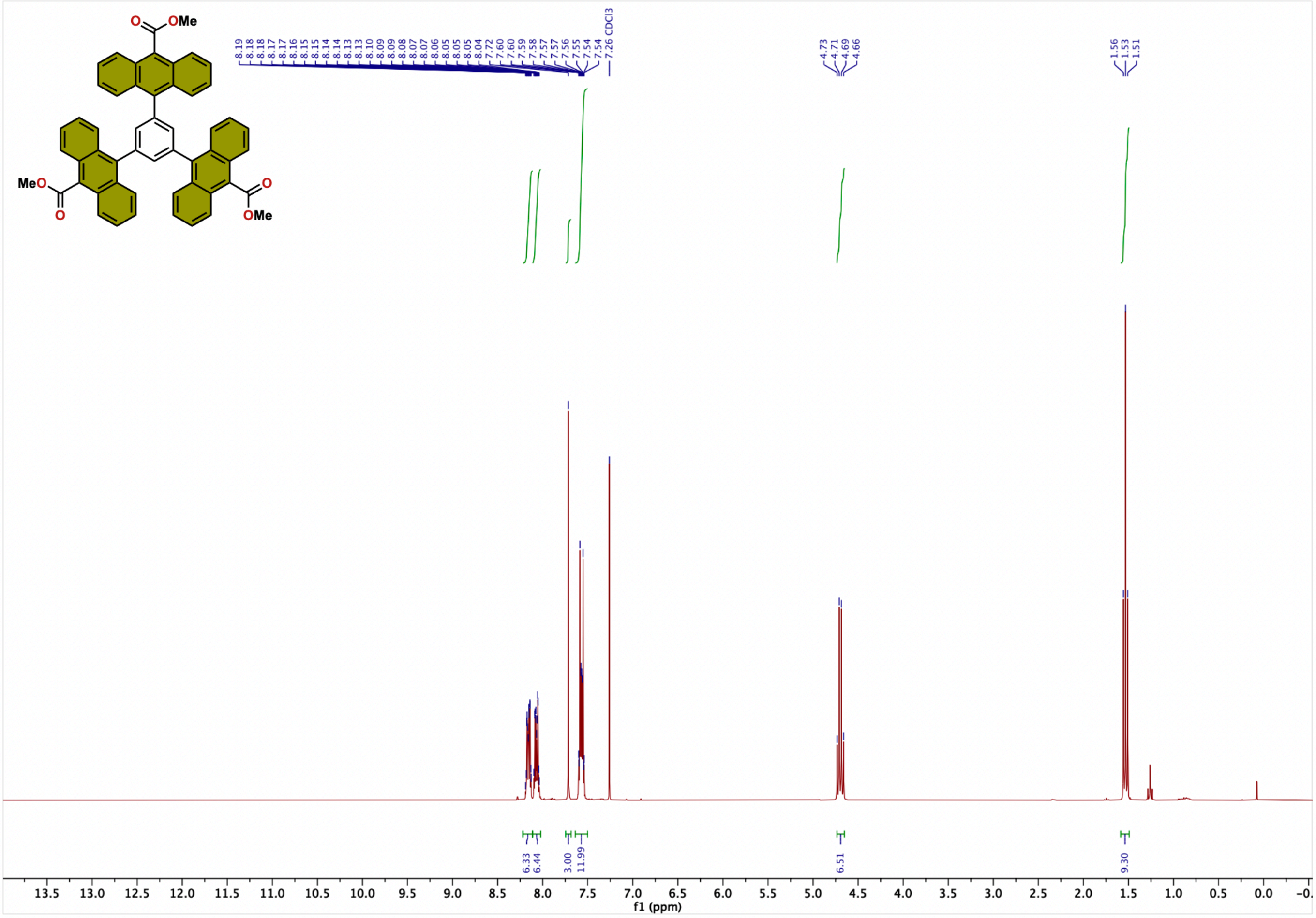

Figure 78: ^{1}H NMR spectrum of compound **9** in $CDCl_3$

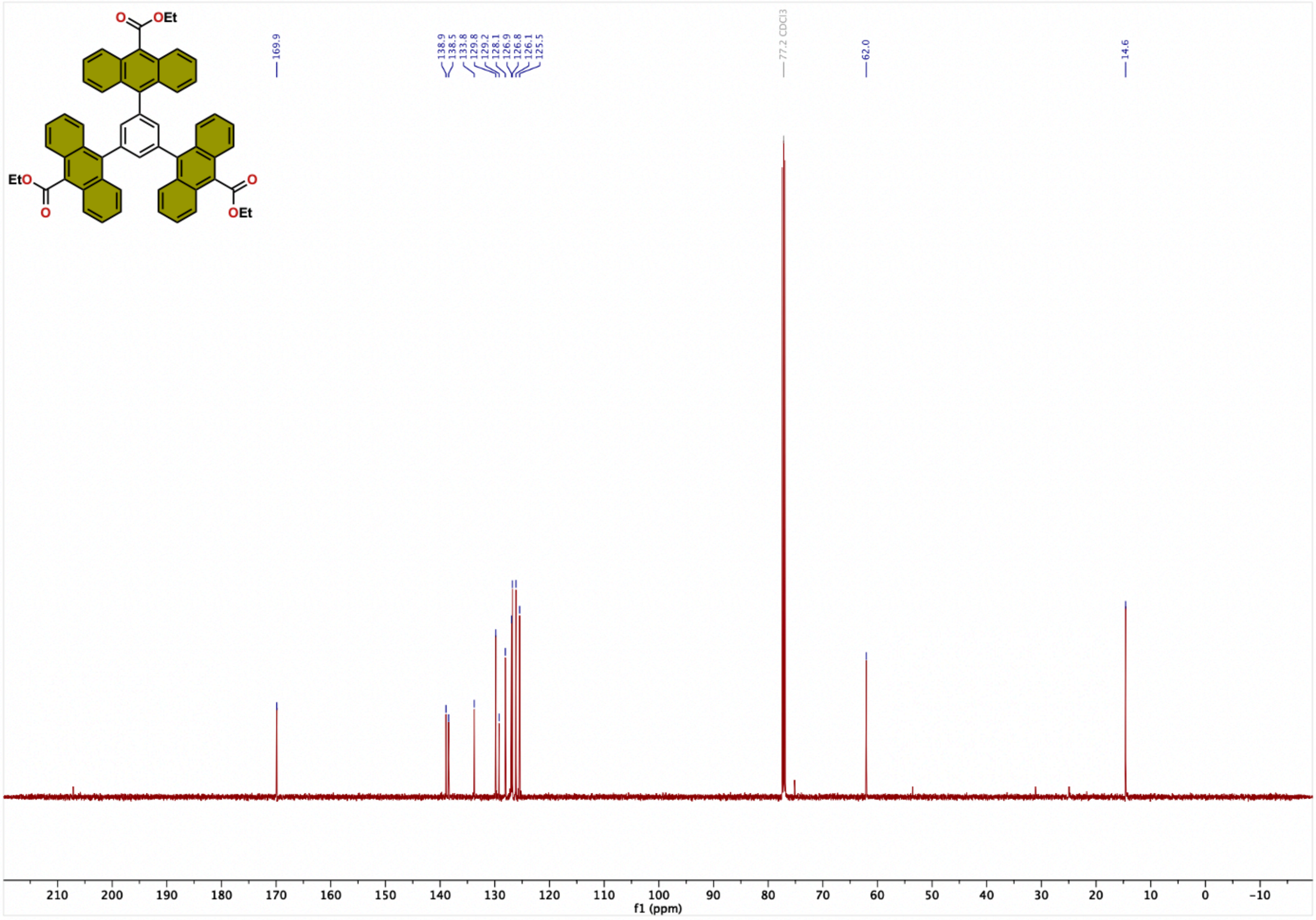

Figure 79: ^{13}C NMR spectrum of compound **9** in $CDCl_3$

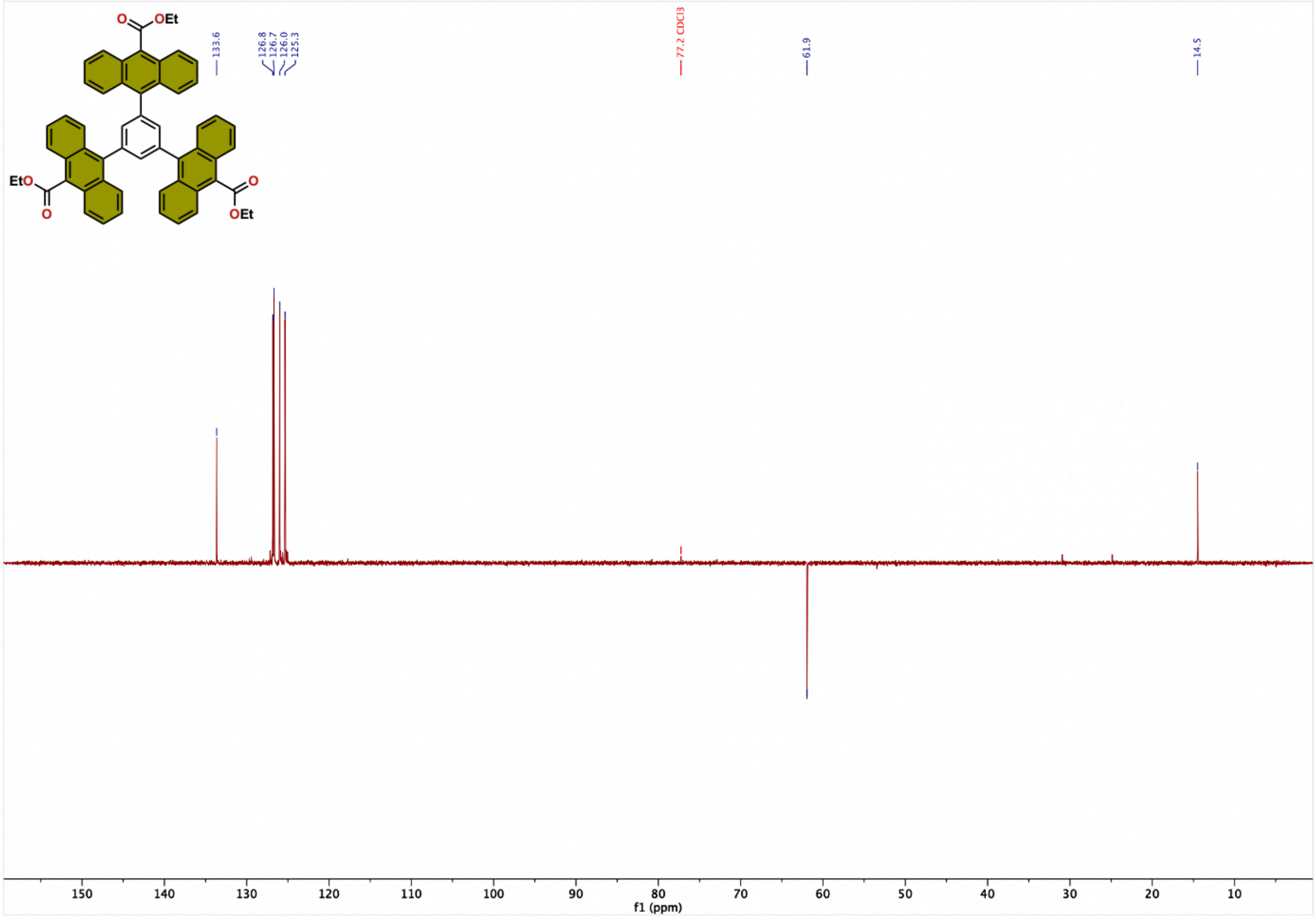

Figure 80: DEPT-135 NMR spectrum of compound **9** in $CDCl_3$

Compound 10:

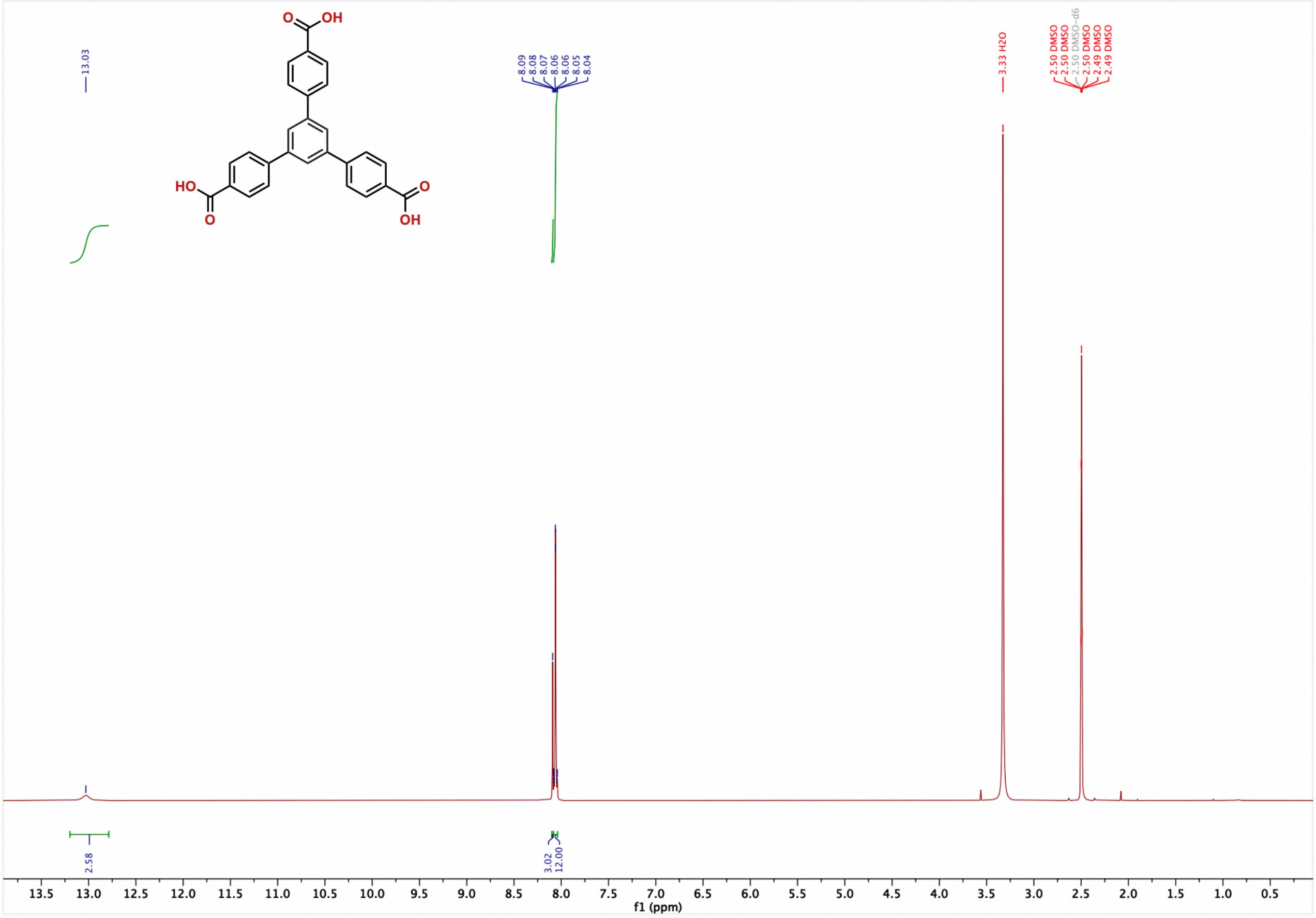

Figure 81: ^{1}H NMR spectrum of compound **10** in DMSO-d_6

Compound 11:

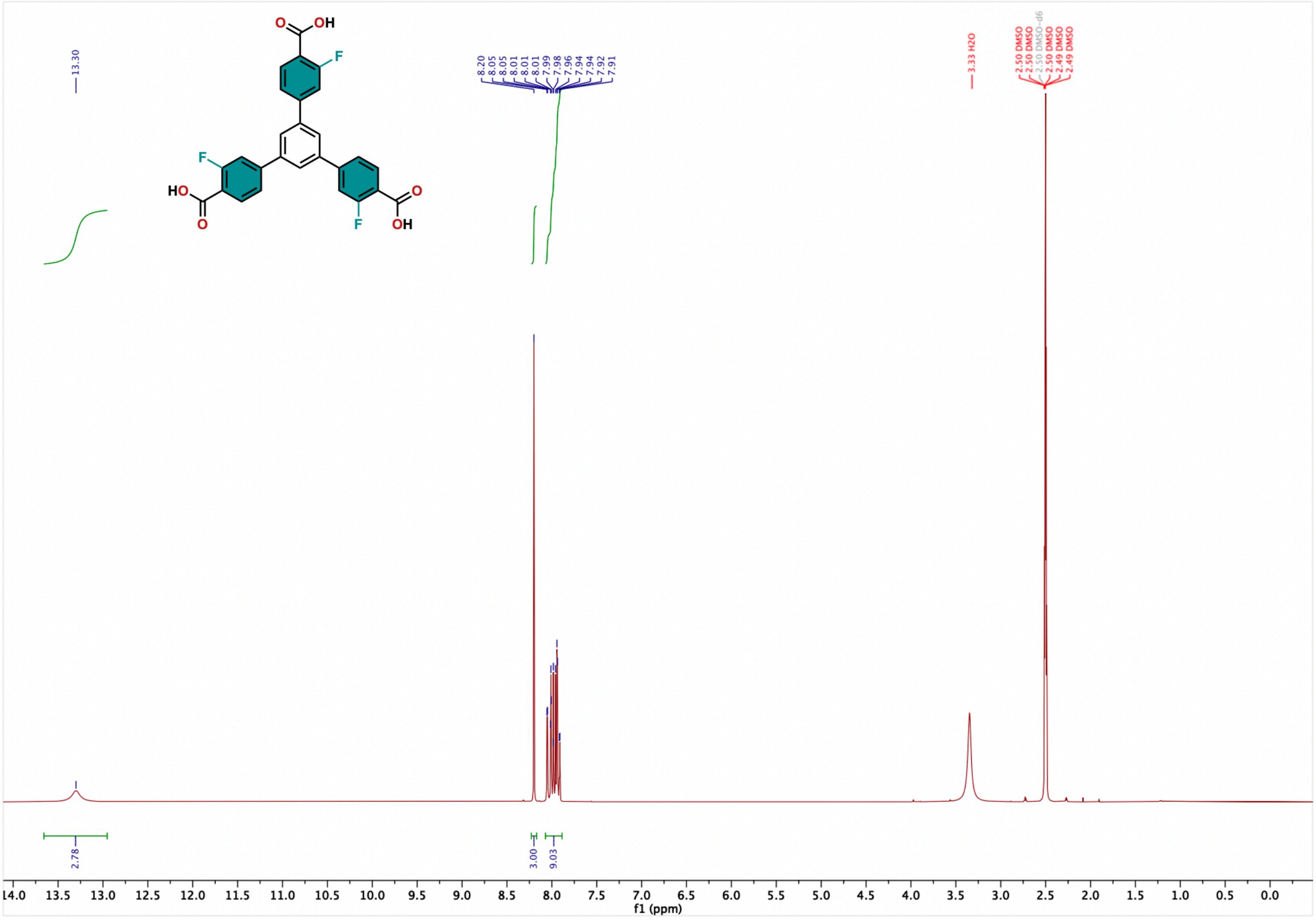

Figure 82: ^{1}H NMR spectrum of compound **11** in DMSO-d_6

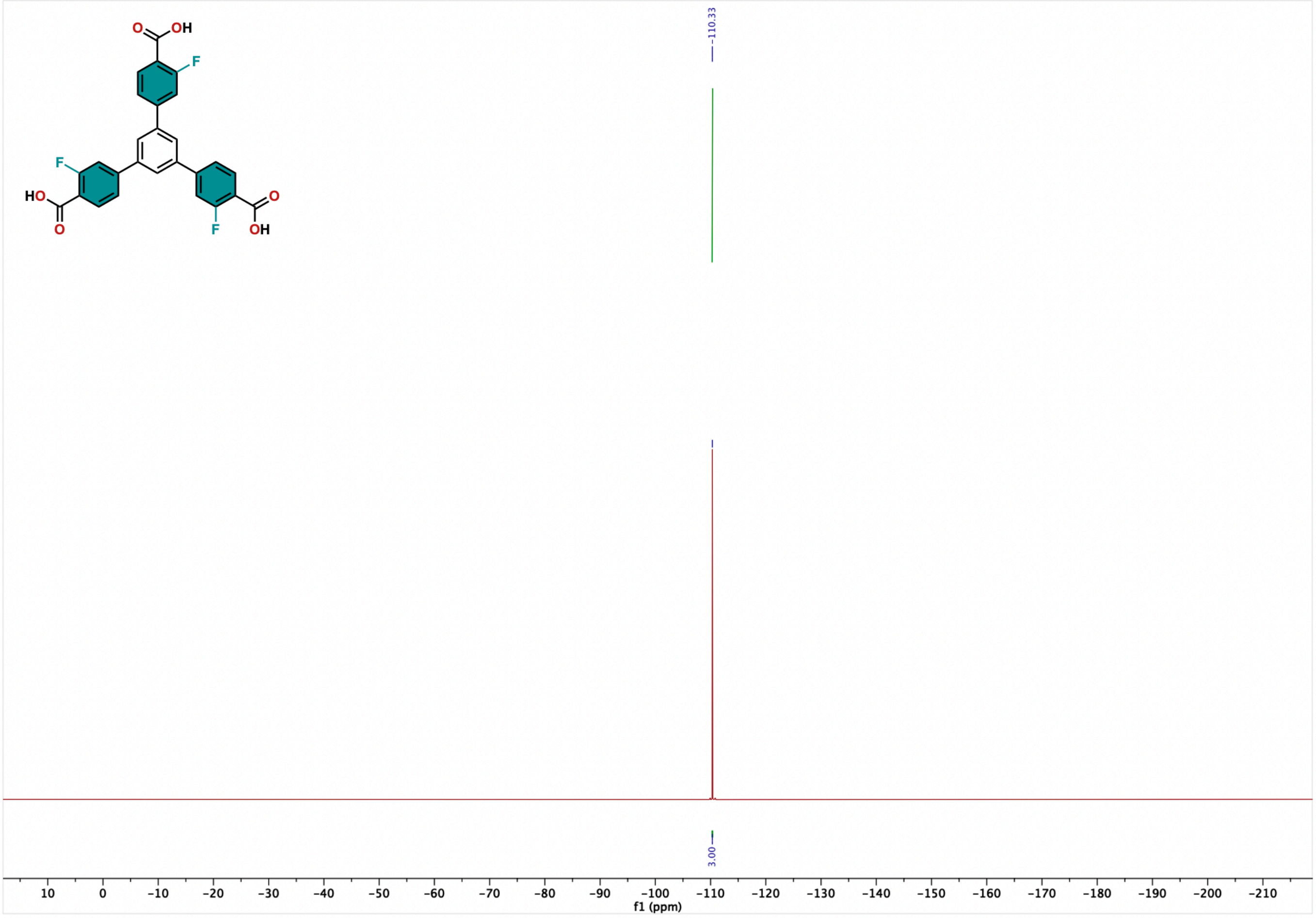

Figure 83: ^{19}F NMR spectrum of compound **11** in DMSO-d_6

Compound 12:

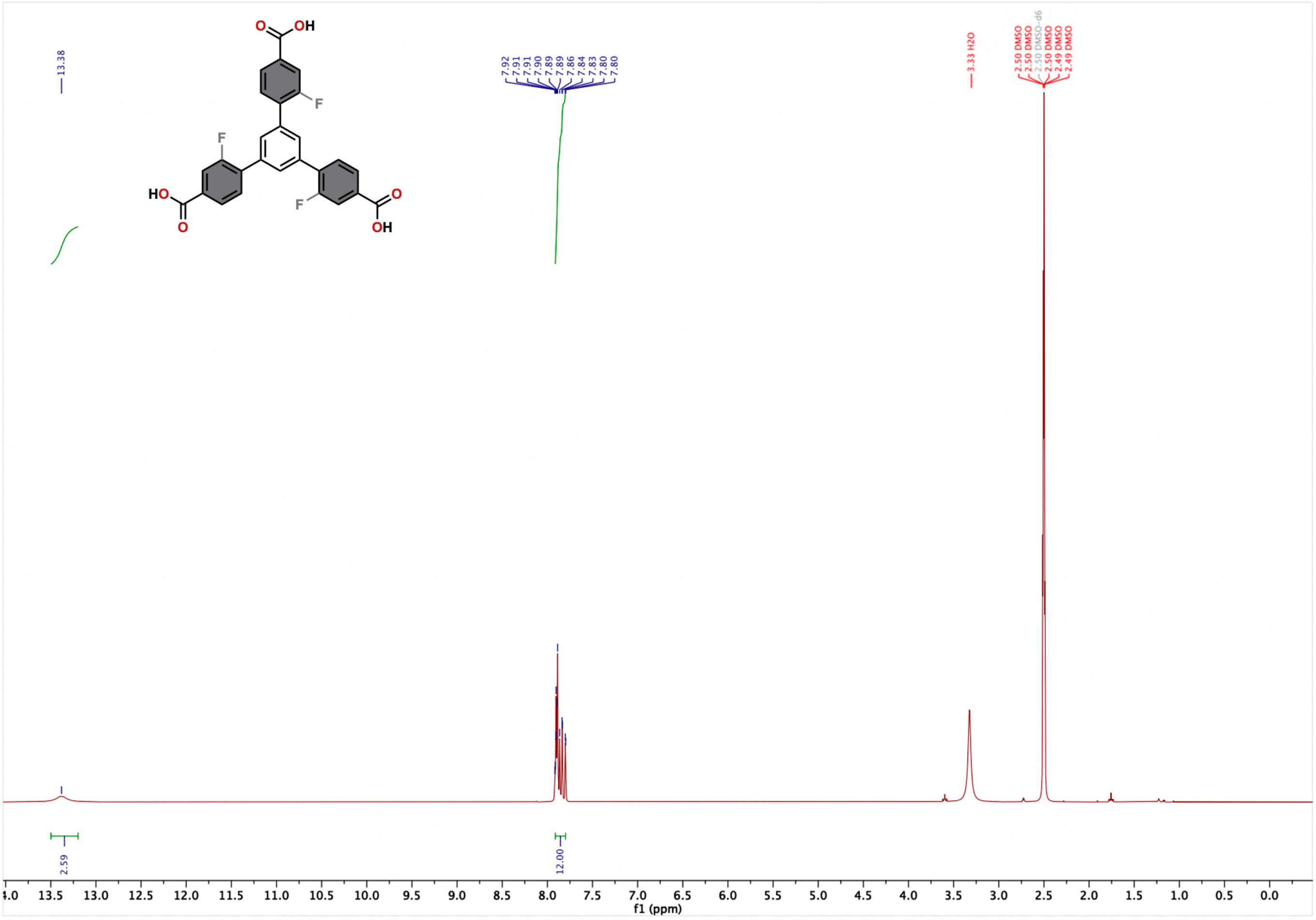

Figure 84: ^{1}H NMR spectrum of compound **12** in DMSO-d_6

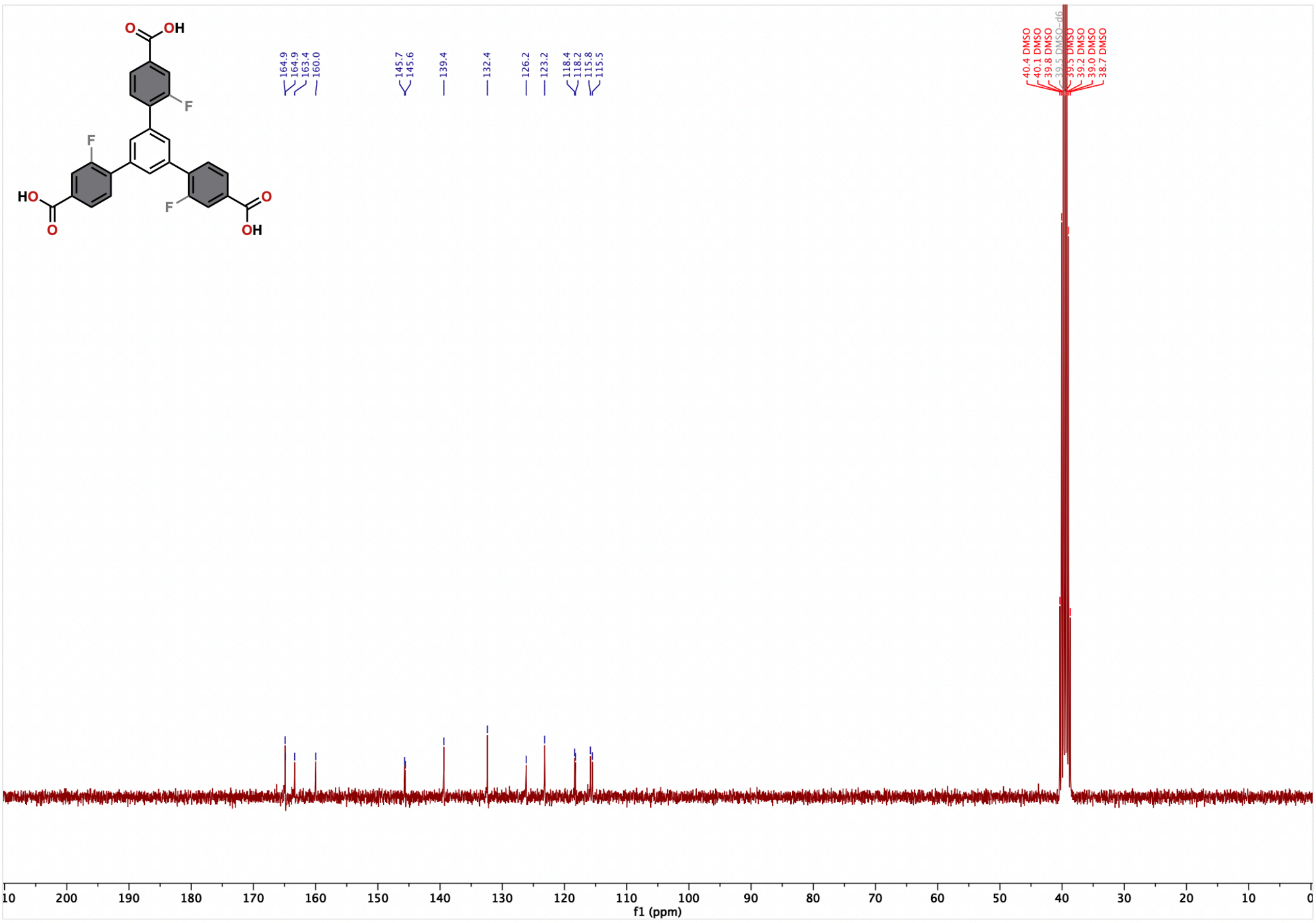

Figure 85: ^{13}C NMR spectrum of compound **12** in DMSO-d_6

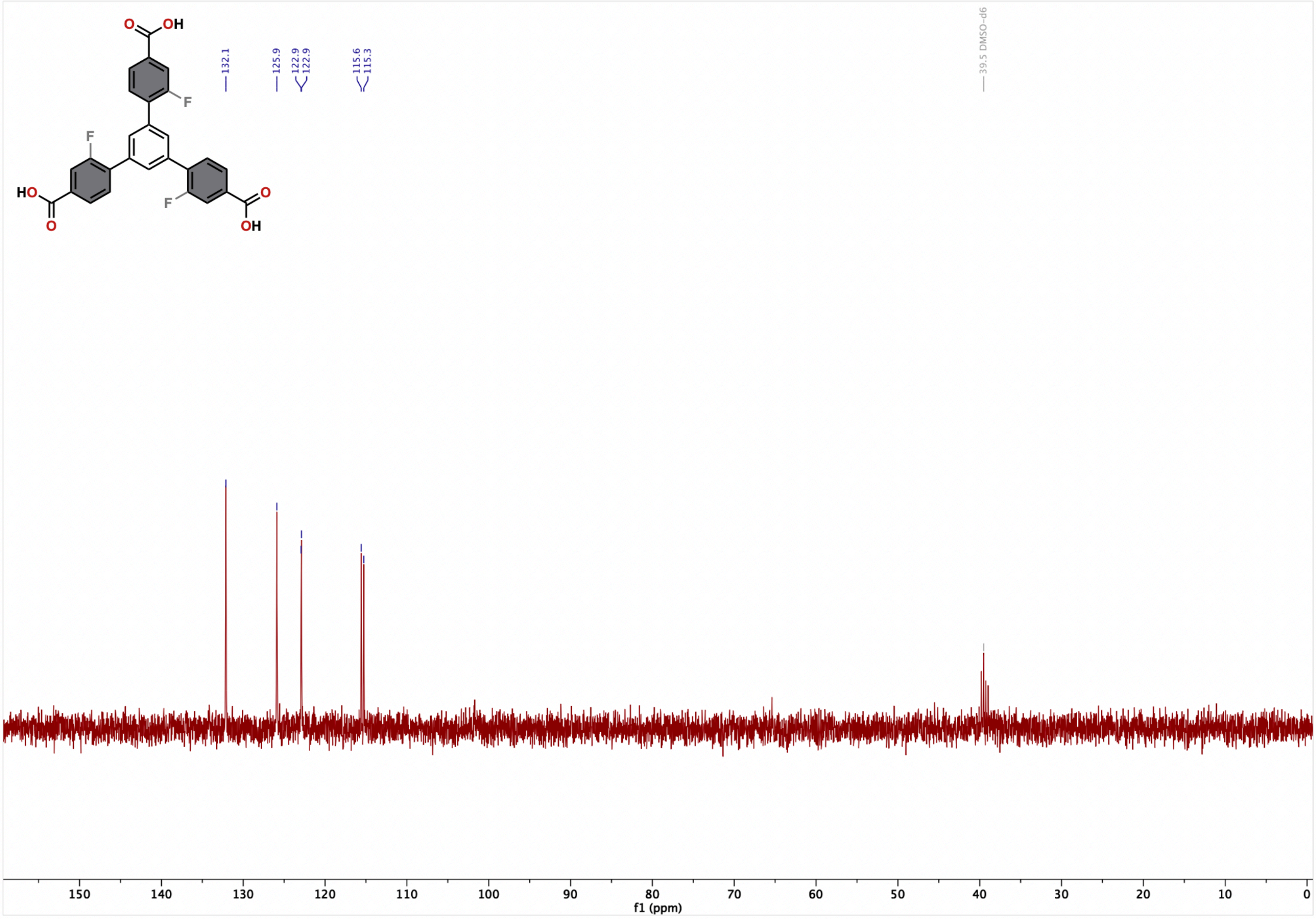

Figure 86: DEPT-135 NMR spectrum of compound **12** in DMSO-d_6

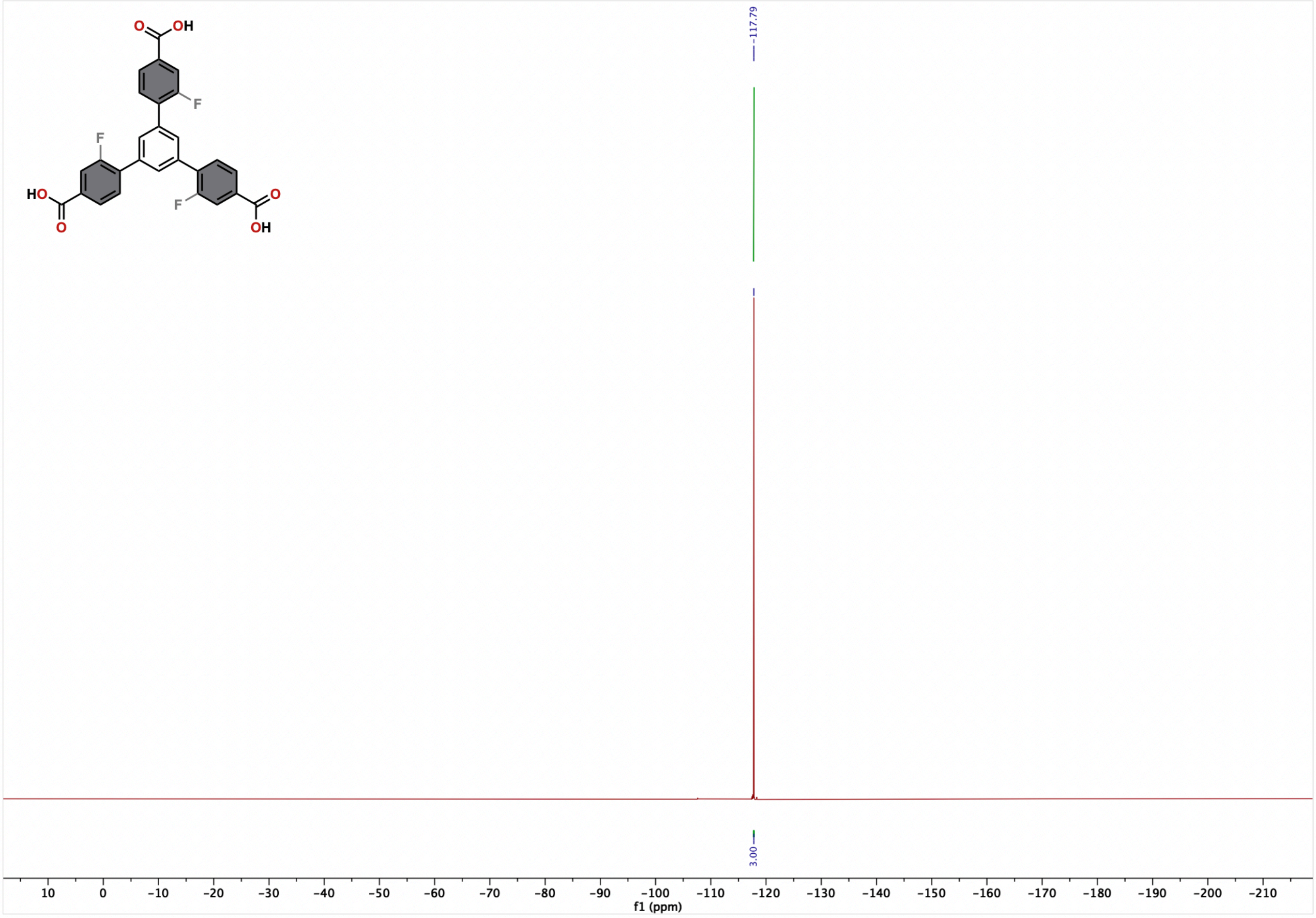

Figure 87: ^{19}F NMR spectrum of compound **12** in DMSO-d_6

Compound 13:

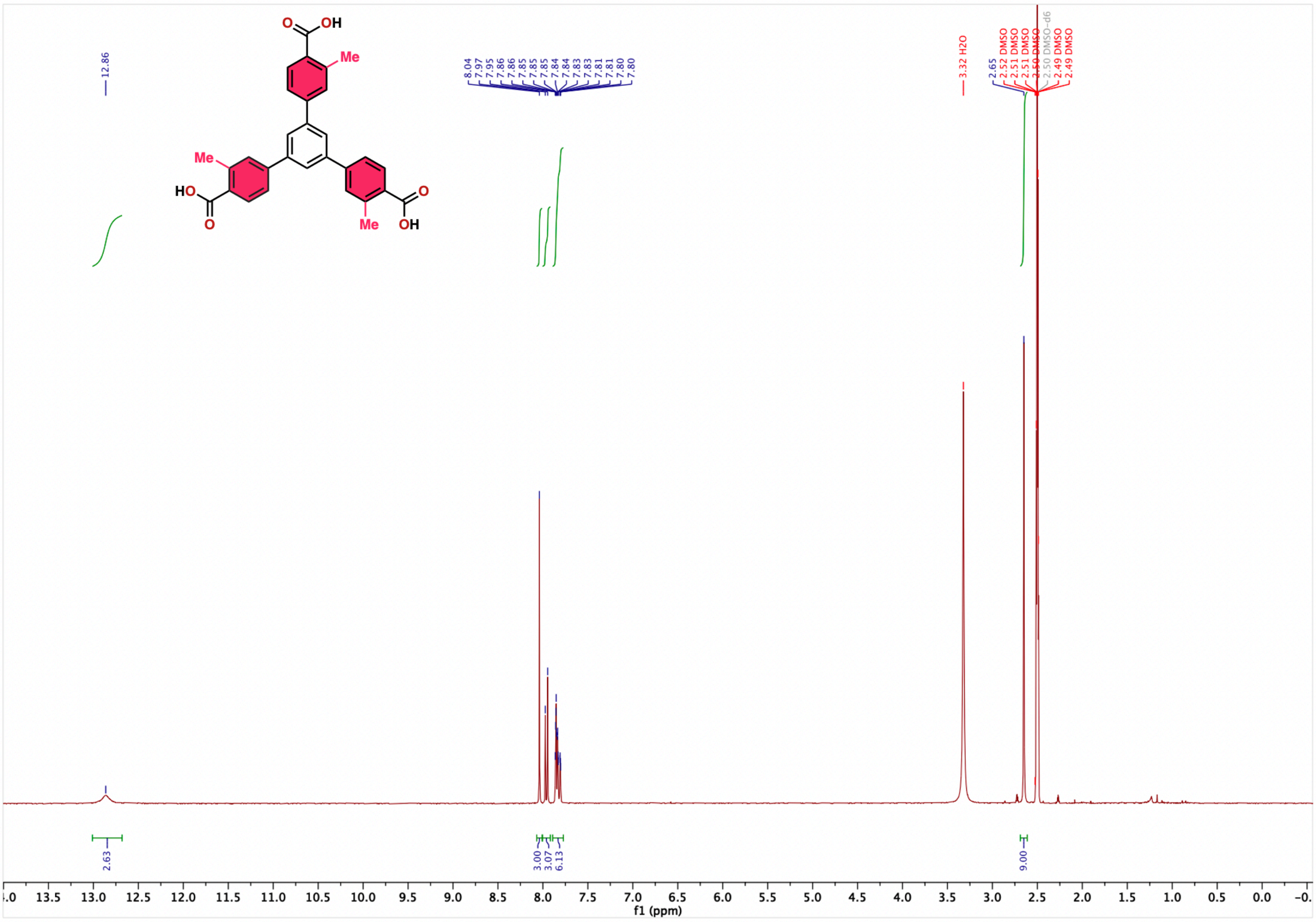

Figure 88: ^{1}H NMR spectrum of compound **13** in DMSO-d_6

Compound 14:

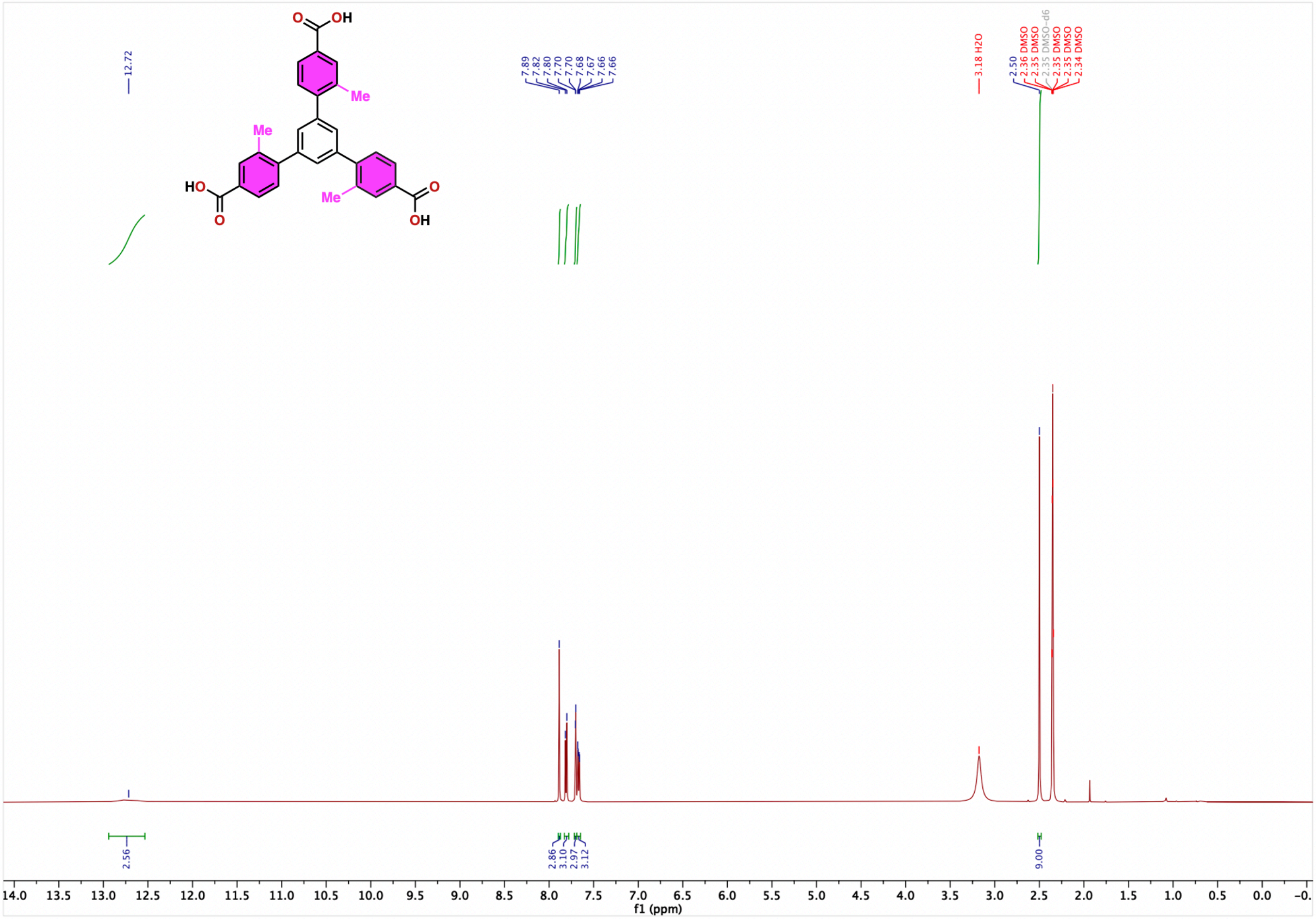

Figure 89: ^{1}H NMR spectrum of compound **14** in DMSO-d_6

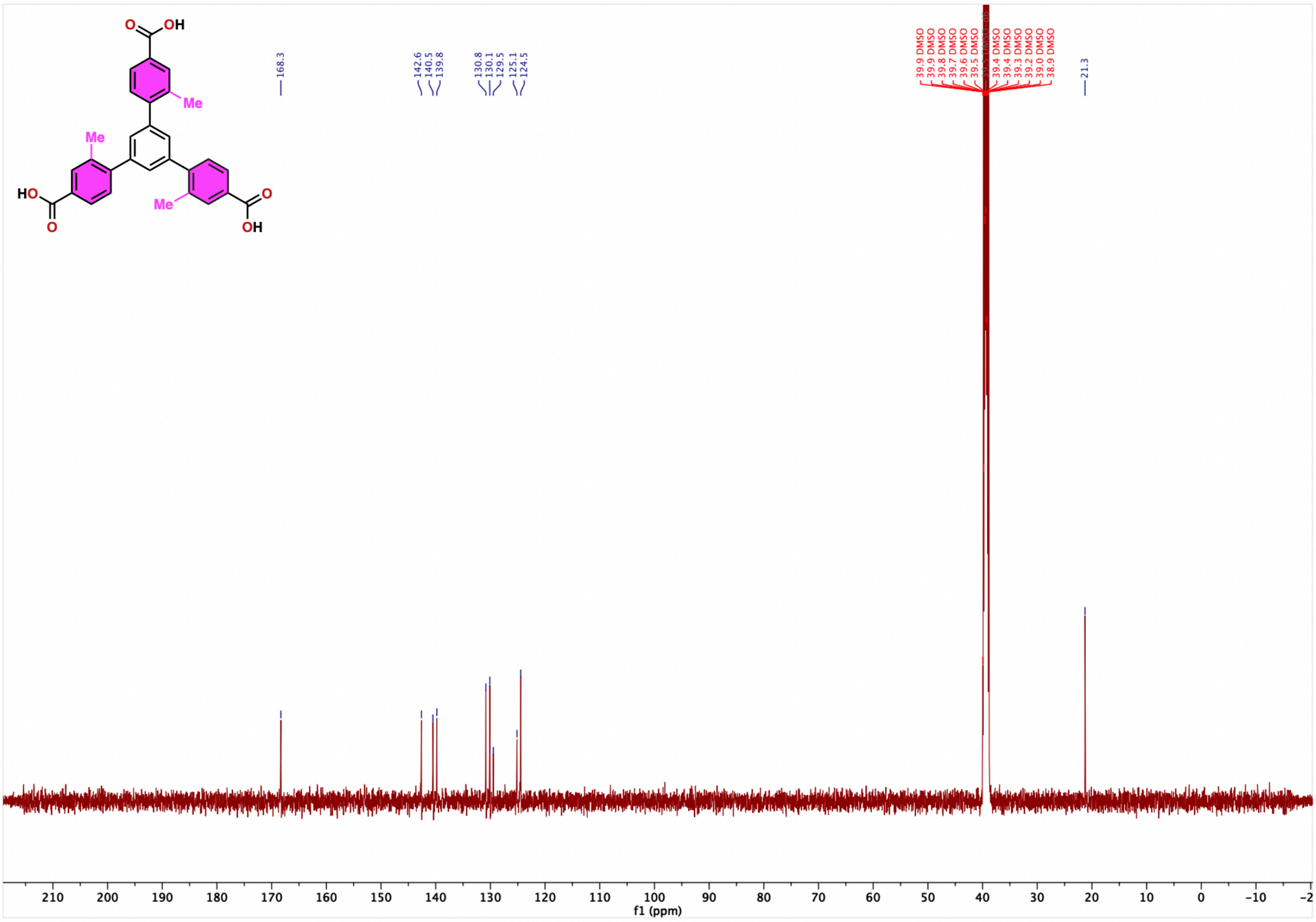

Figure 90: ^{13}C NMR spectrum of compound **14** in DMSO-d_6

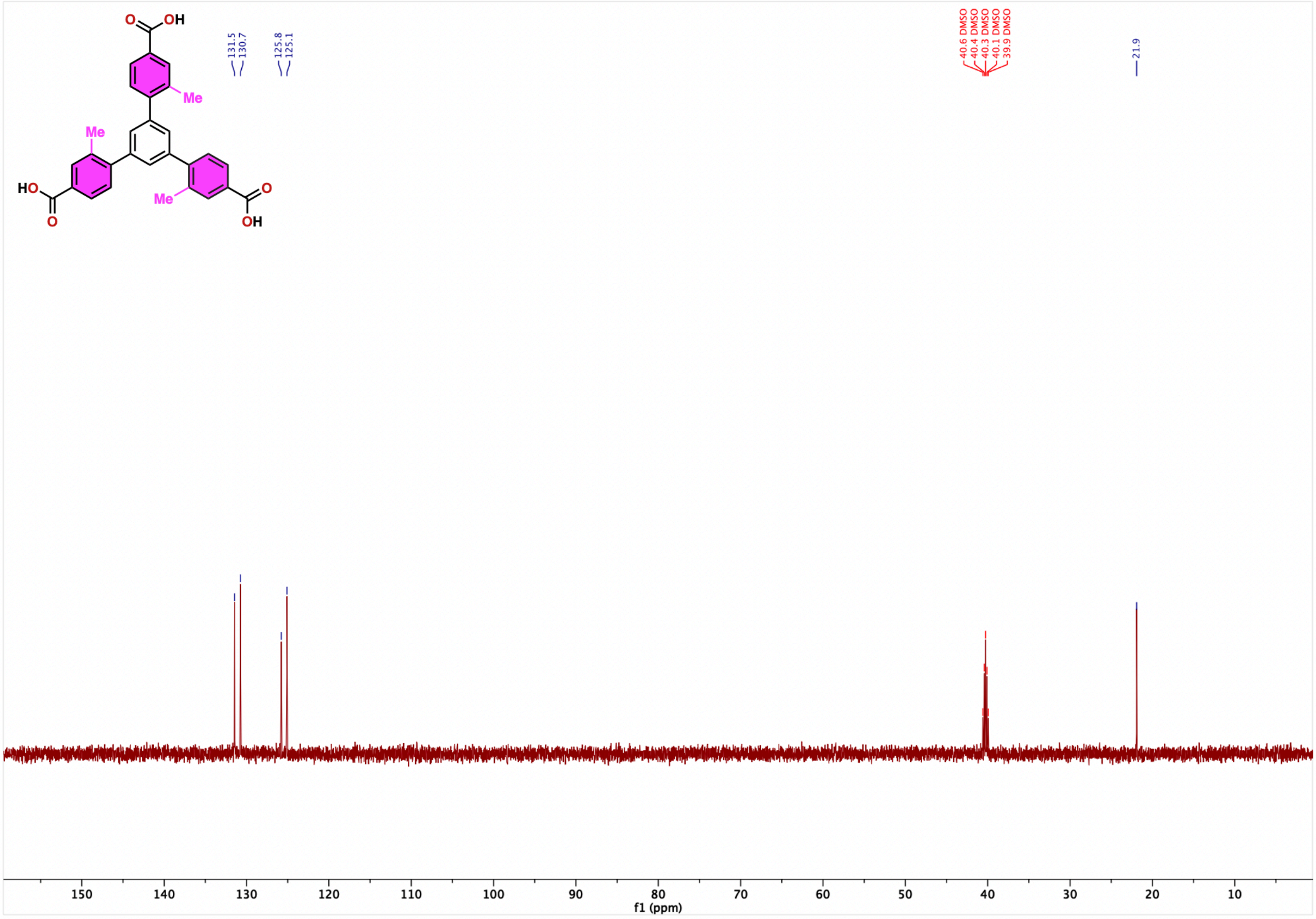

Figure 91: DEPT-135 NMR spectrum of compound **14** in DMSO-d_6

Compound 15:

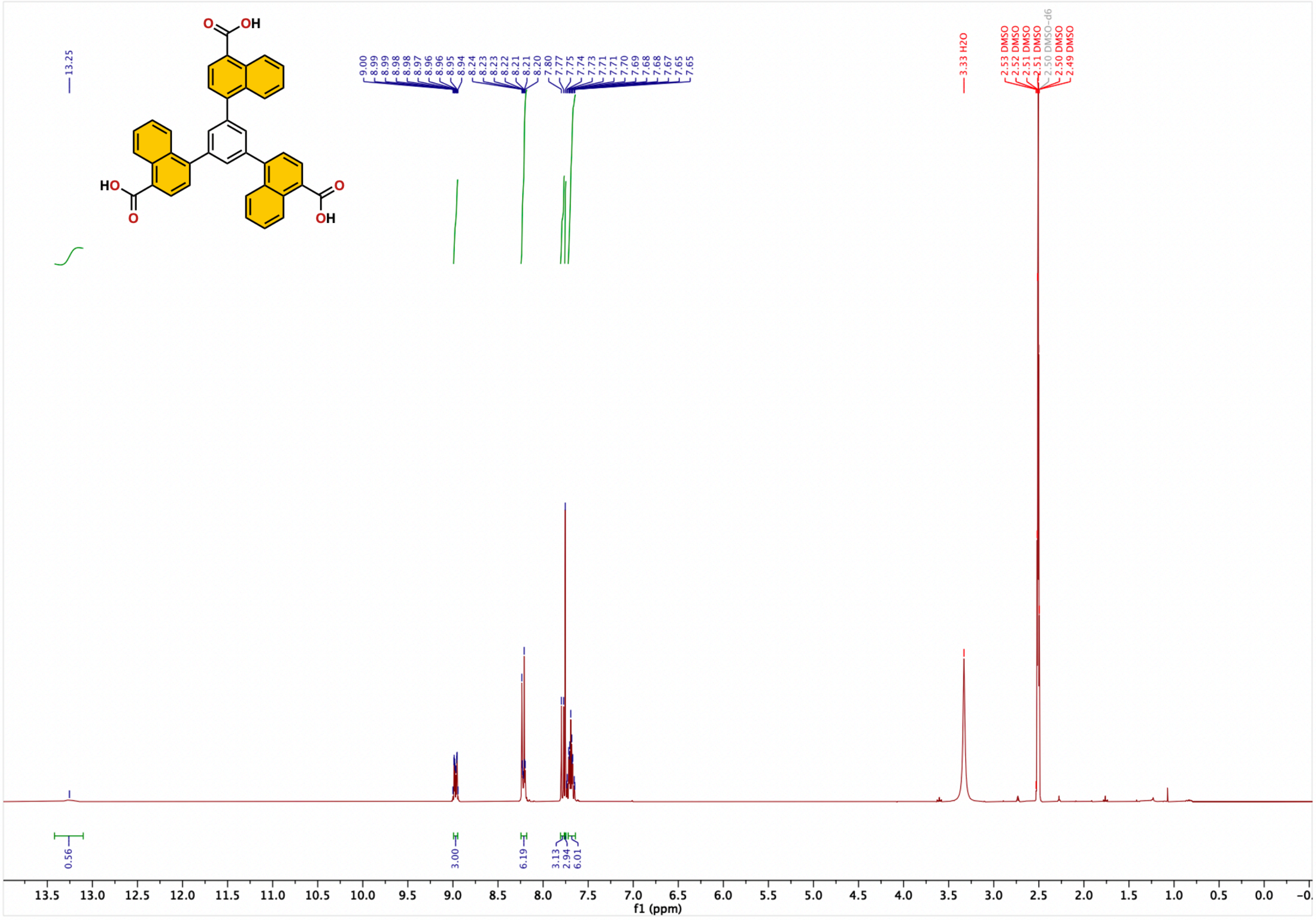

Figure 92: ^{1}H NMR spectrum of compound **15** in DMSO-d_6

Compound 16:

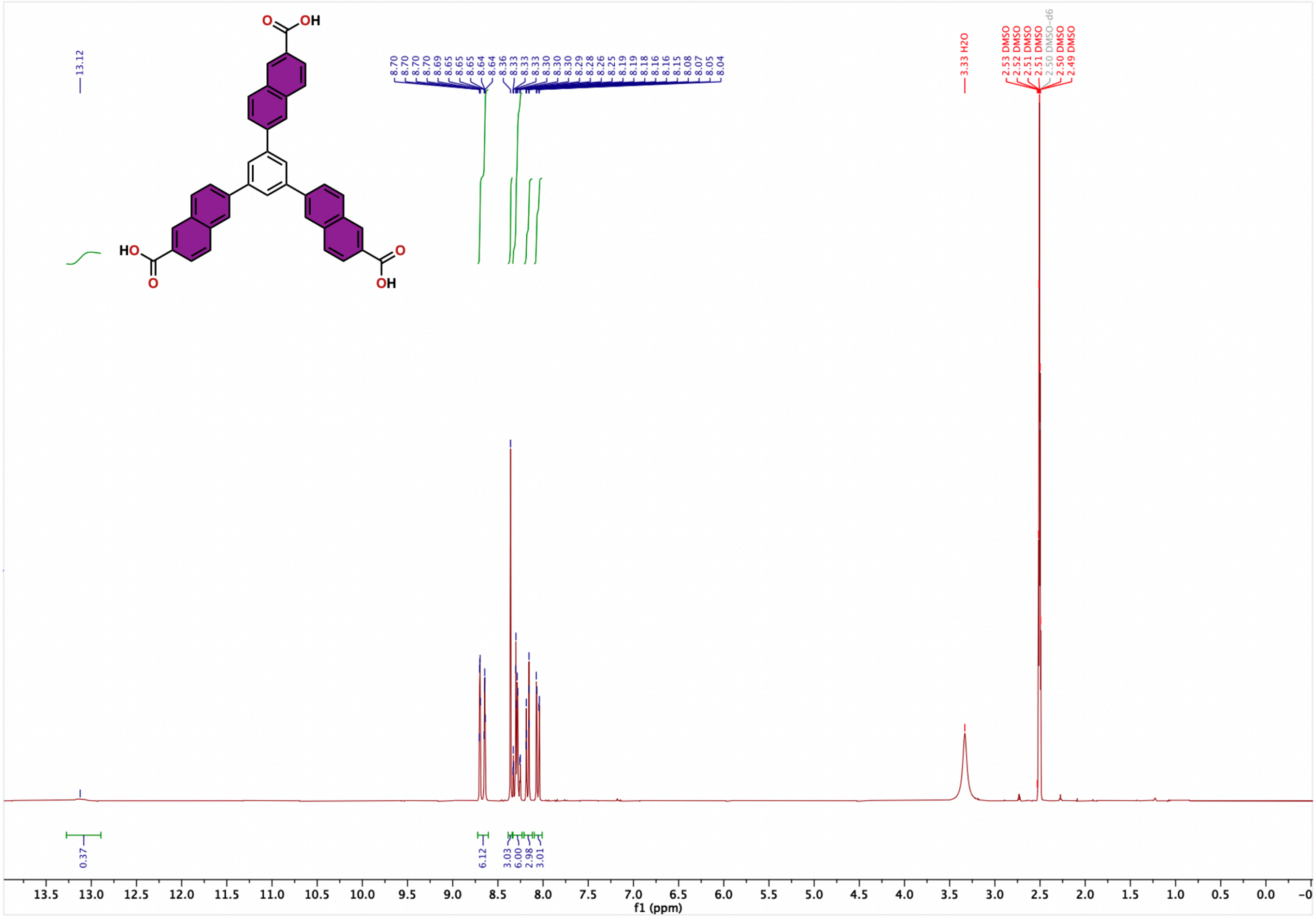

Figure 93: ^{1}H NMR spectrum of compound **16** in DMSO-d_6.

Compound 17:

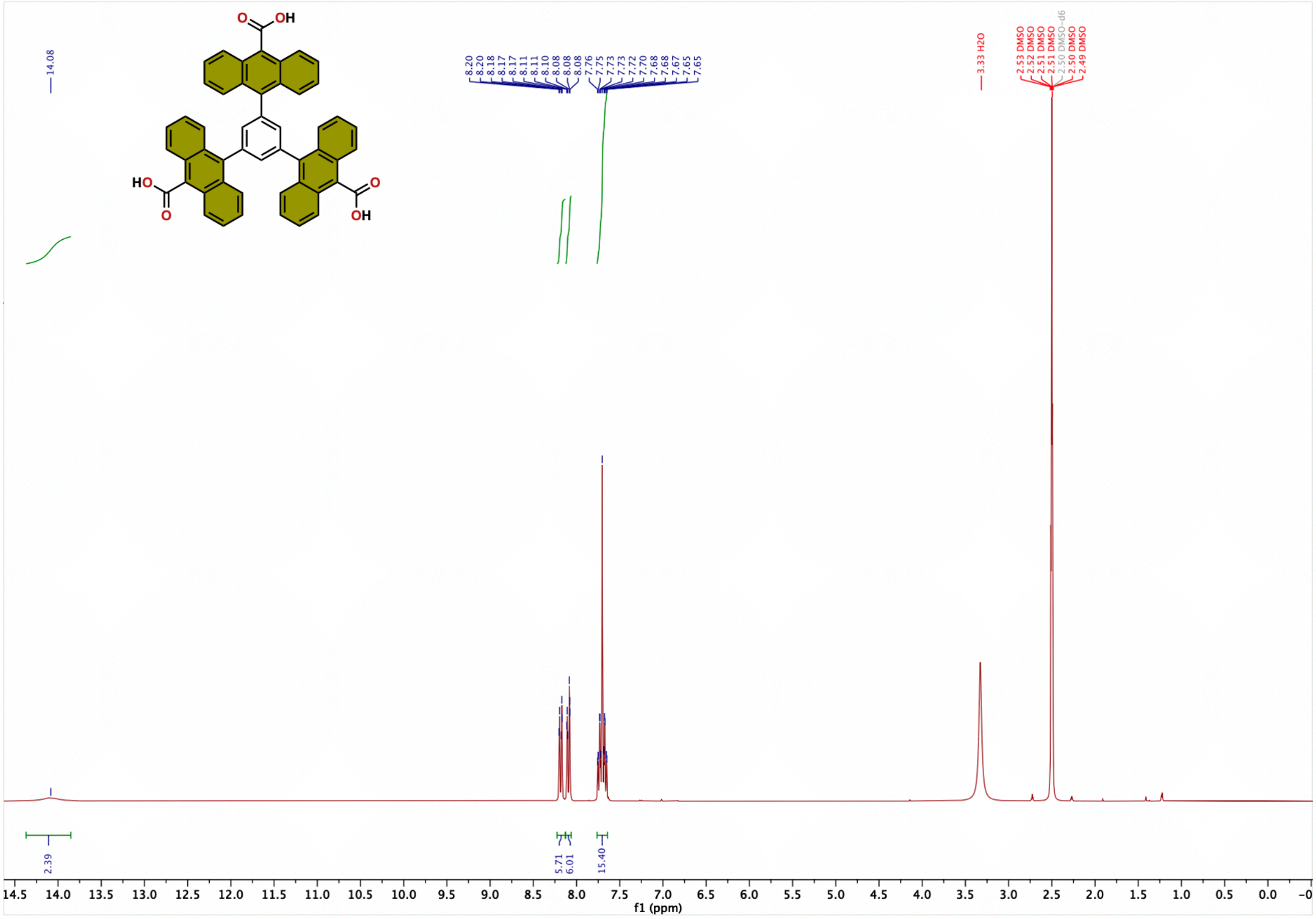

Figure 94: ^{1}H NMR spectrum of compound **17** in DMSO-d_6

.

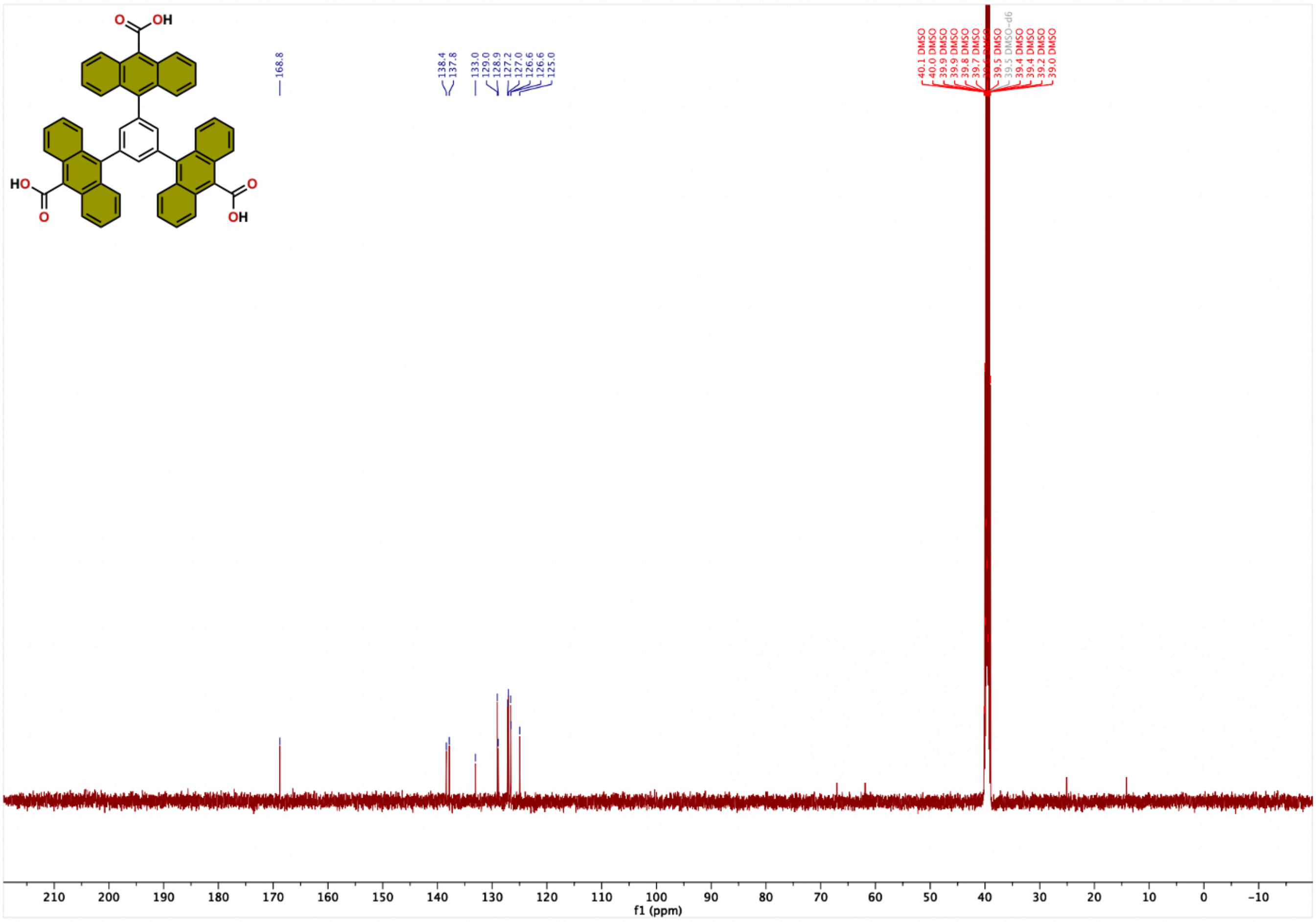

Figure 95: ^{13}C NMR spectrum of compound **17** in DMSO-d_6

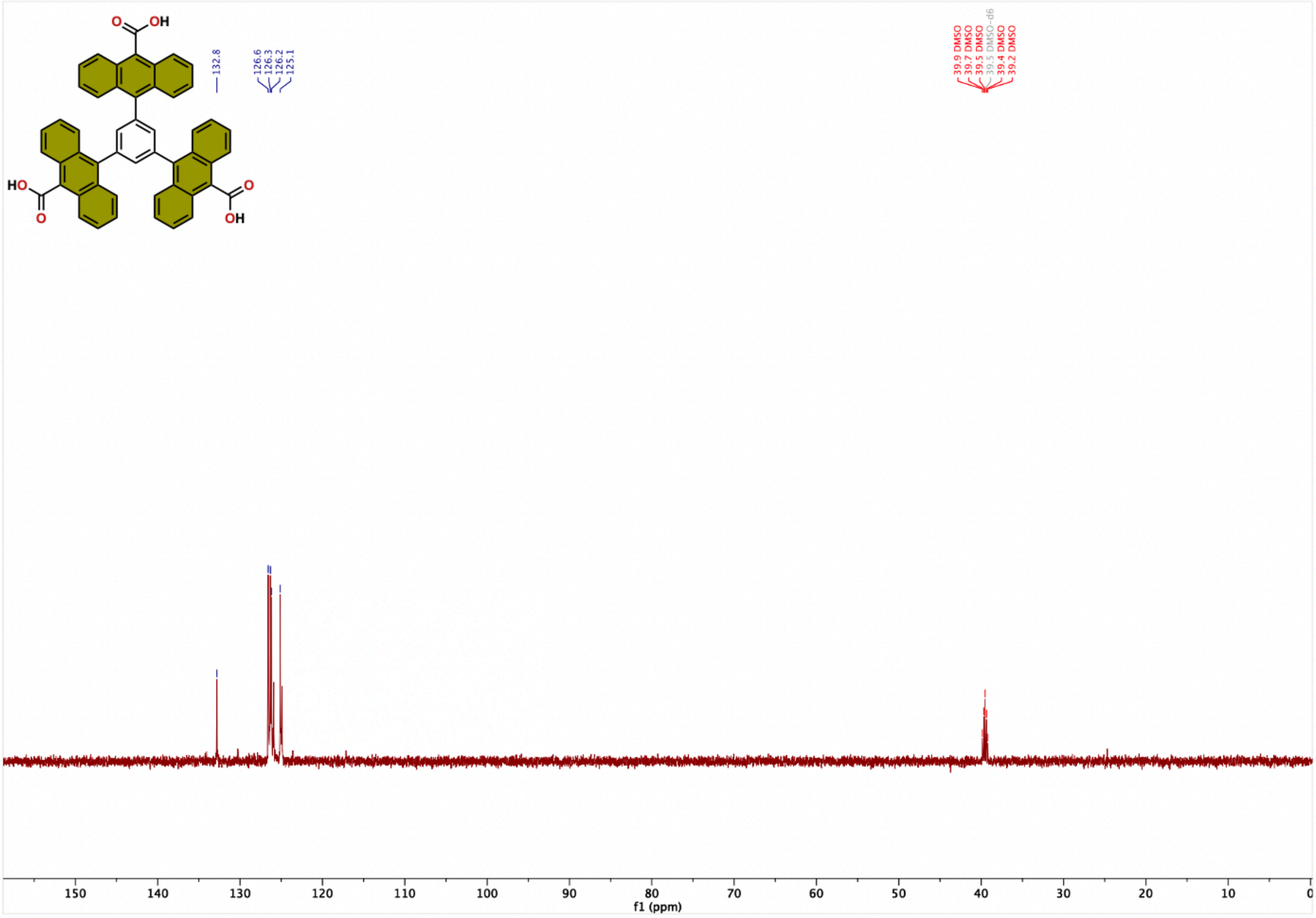

Figure 96: DEPT-135 NMR spectrum of compound **17** in DMSO-d_6

S.11. REFERENCES

(1) Hong, K.; Bak, W.; Chun, H. Robust Molecular Crystals of Titanium (IV)-Oxo-Carboxylate Clusters Showing Water Stability and CO_2 Sorption Capability. *Inorg. Chem.* **2014**, *53* (14), 7288–7293. https://doi.org/10.1021/ic500629y.

(2) Coelho, A. A. TOPAS and TOPAS-Academic: An Optimization Program Integrating Computer Algebra and Crystallographic Objects Written in C++. *J. Appl. Crystallogr.* **2018**, *51* (1), 210–218. https://doi.org/10.1107/s1600576718000183.

(3) Zhang, Y.-B.; Furukawa, H.; Ko, N.; Nie, W.; Park, H. J.; Okajima, S.; Cordova, K. E.; Deng, H.; Kim, J.; Yaghi, O. M. Introduction of Functionality, Selection of Topology, and Enhancement of Gas Adsorption in Multivariate Metal–Organic Framework-177. *J. Am. Chem. Soc.* **2015**, *137* (7), 2641–2650. https://doi.org/10.1021/ja512311a.

(4) Iannazzo, L.; Vollhardt, K. P. C.; Malacria, M.; Aubert, C.; Gandon, V. Alkynylboronates and ‐ boramides in CoI ‐ and RhI ‐ Catalyzed [2+2+2] Cycloadditions: Construction of Oligoaryls through Selective Suzuki Couplings. *Eur. J. Org. Chem.* **2011**, (18), 3283–3292. https://doi.org/10.1002/ejoc.201100371.

(5) Liu, H.; He, Y.; Jiao, J.; Bai, D.; Chen, D.; Krishna, R.; Chen, B. A Porous Zirconium ‐ Based Metal ‐ Organic Framework with the Potential for the Separation of Butene Isomers. *Chem. European. J.* **2016**, *22* (42), 14988–14997. https://doi.org/10.1002/chem.201602892.

(6) a) He, Y.; Zhang, Z.; Xiang, S.; Fronczek, F. R.; Krishna, R.; Chen, B. A Robust Doubly Interpenetrated Metal–Organic Framework Constructed from a Novel Aromatic Tricarboxylate for Highly Selective Separation of Small Hydrocarbons. *Chem. Commun.* **2012**, *48* (52), 6493–6495. https://doi.org/10.1039/c2cc31792c. b) High $CO_2/N_2/O_2/CO$ Separation in a Chemically Robus Porous Coordination Polymer with Low Binding Energy, Duan, J.; Higuchi, M; Krishna, R. ; Kiyonaga, T.; Tsutsumi, Y.; Sato, Y.; Kubota,Y.; Takata, M.; Kitagawa, S., *Chem. Sci.*, **2014**, *5* 660-666. https://doi.org/10.1039/C3SC52177J. c) A new mesoporous coordination polymer: synthesis, structure, and gas adsorption studies. Duan, J.; Li, Q.; Lu, Z., *CrystEngComm*, **2015**, *17*, 2087-2090. https://doi.org/10.1039/C5CE00015G

(7) Ibarra, I. A.; Lin, X.; Yang, S.; Blake, A. J.; Walker, G. S.; Barnett, S. A.; Allan, D. R.; Champness, N. R.; Hubberstey, P.; Schröder, M. Structures and H2 Adsorption Properties of Porous Scandium Metal–Organic Frameworks. *Chem. European. J.* **2010**, *16* (46), 13671–13679. https://doi.org/10.1002/chem.201000926.

(8) López ‐ Maya, E.; M. Padial, N.; Castells ‐ Gil, J.; Ganivet, C. R.; Rubio ‐ Gaspar, A.; Cirujano, F. G.; Almora ‐ Barrios, N.; Tatay, S.; Navalón, S.; Martí ‐ Gastaldo, C. Selective Implantation of Diamines for Cooperative Catalysis in Isoreticular Heterometallic Titanium–Organic Frameworks. *Angew. Chem. Inter. Ed.* **2021**, *133* (21), 11975–11980. https://doi.org/10.1002/ange.202100176.

(9) Bourhis, L. J.; Dolomanov, O. V.; Gildea, R. J.; Howard, J. A. K.; Puschmann, H. The Anatomy of a Comprehensive Constrained, Restrained Refinement Program for the Modern Computing Environment – Olex2 Dissected. *Acta. Crystallogr. A.* **2015**, *71* (1), 59–75. https://doi.org/10.1107/s2053273314022207.

(10) Sheldrick, G. M. SHELXT – Integrated Space-Group and Crystal-Structure Determination. *Acta Crystallogr. Sect. Found. Adv.* **2015**, *71* (1), 3–8. https://doi.org/10.1107/s2053273314026370.

(11) Dolomanov, O. V.; Bourhis, L. J.; Gildea, R. J.; Howard, J. A. K.; Puschmann, H. OLEX2: A Complete Structure Solution, Refinement and Analysis Program. *J. Appl. Crystallogr*. **2009**, *2* (2), 339–341. https://doi.org/10.1107/s0021889808042726.

(12) Howarth, A. J.; Peters, A. W.; Vermeulen, N. A.; Wang, T. C.; Hupp, J. T.; Farha, O. K. Best Practices for the Synthesis, Activation, and Characterization of Metal–Organic Frameworks. *Chem. Mater.* **2017**, *29* (1), 26–39. https://doi.org/10.1021/acs.chemmater.6b02626.

(13) Maglic, J. B.; Lavendomme, R. MoloVol: An Easy-to-Use Program for Analyzing Cavities, Volumes and Surface Areas of Chemical Structures. *J. Appl. Crystallogr.* **2022**, *55* (Pt 4), 1033–1044. https://doi.org/10.1107/s1600576722004988.

(14) Makuła, P.; Pacia, M.; Macyk, W. How To Correctly Determine the Band Gap Energy of Modified Semiconductor Photocatalysts Based on UV–Vis Spectra. *The J. Phys. Chem. Lett.* **2018**, *9* (23), 6814–6817. https://doi.org/10.1021/acs.jpclett.8b02892.

(15) Kresse, G.; Furthmüller, J. Efficient Iterative Schemes for Ab Initio Total-Energy Calculations Using a Plane-Wave Basis Set. *Phys. Rev. B* **1996**, *54* (16), 11169–11186. https://doi.org/10.1103/physrevb.54.11169.

(16) Perdew, J. P.; Burke, K.; Ernzerhof, M. Generalized Gradient Approximation Made Simple. *Phys. Rev. Lett*. **1996**, *78* (7), 1396–1396. https://doi.org/10.1103/physrevlett.78.1396.

(17) Perdew, J. P.; Burke, K.; Ernzerhof, M. Generalized Gradient Approximation Made Simple. *Phys. Rev. Lett.* **1996**, *77* (18), 3865–3868. https://doi.org/10.1103/physrevlett.77.3865.

(18) Grimme, S.; Antony, J.; Ehrlich, S.; Krieg, H. A Consistent and Accurate Ab Initio Parametrization of Density Functional Dispersion Correction (DFT-D) for the 94 Elements H-Pu. *J. Chem. Phys.* **2010**, *132* (15), 154104. https://doi.org/10.1063/1.3382344.

(19) Bučko, T.; Hafner, J.; Lebègue, S.; Ángyán, J. G. Improved Description of the Structure of Molecular and Layered Crystals: Ab Initio DFT Calculations with van Der Waals Corrections. *J. Phys. Chem.* **2010**, *114* (43), 11814–11824. https://doi.org/10.1021/jp106469x.

(20) Kresse, G.; Joubert, D. From Ultrasoft Pseudopotentials to the Projector Augmented-Wave Method. *Phys. Rev. B.* **1998**, *59* (3), 1758–1775. https://doi.org/10.1103/physrevb.59.1758.
